# Supplementary material for: Phenotypic pleiotropy of missense variants in human B cell confinement receptor P2RY8
Source: Cell Genom. 2025 Sep 9;5(11):100981. doi: 10.1016/j.xgen.2025.100981 (PMC12648108; doi:10.1016/j.xgen.2025.100981)
Supplement: Document S1. Figures S1–S10 and Table S3 [file mmc1.pdf]

Cell Genomics, Volume *5*

Supplemental information

Phenotypic pleiotropy of missense variants

in human B cell confinement receptor P2RY8

Taylor N. LaFlam, Christian B. Billesbølle, Tuan Dinh, Finn D. Wolfreys, Erick Lu, Tomas Matteson, Jinping An, Ying Xu, Arushi Singhal, Nadav Brandes, Vasilis Ntranos, Aashish Manglik, Jason G. Cyster, and Chun Jimmie Ye

Figure S1

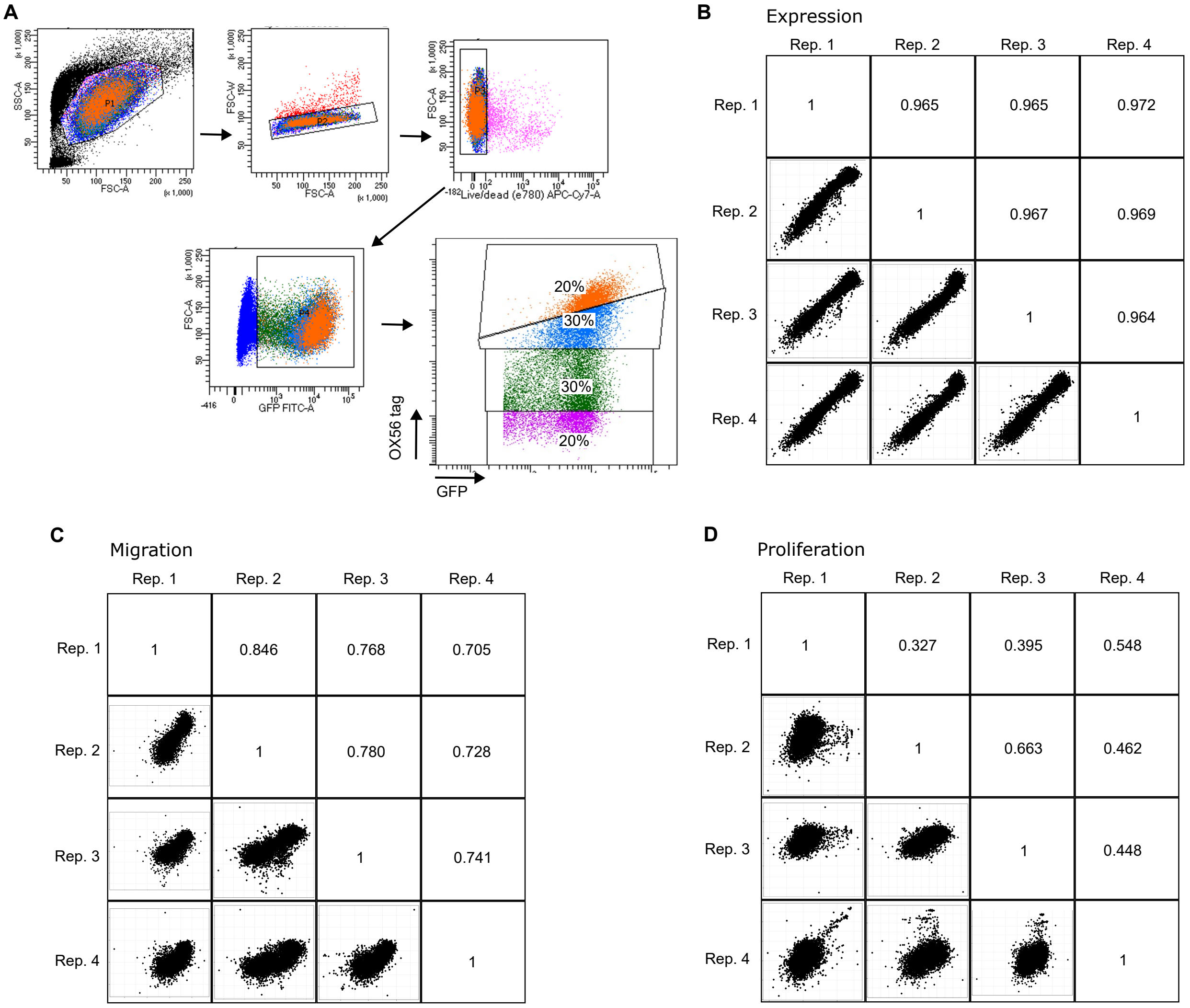

Figure S1: Comparison of DMS replicates, related to Figure 1. (A) Representative flow-assisted cell sorting gating used for surface expression portion of screen. **(B-D)** Dot plots and Pearson correlations of Enrich2 variant scores for each combination of the four replicates for **(B)** surface expression, **(C)** migration, and **(D)** proliferation.

Figure S2

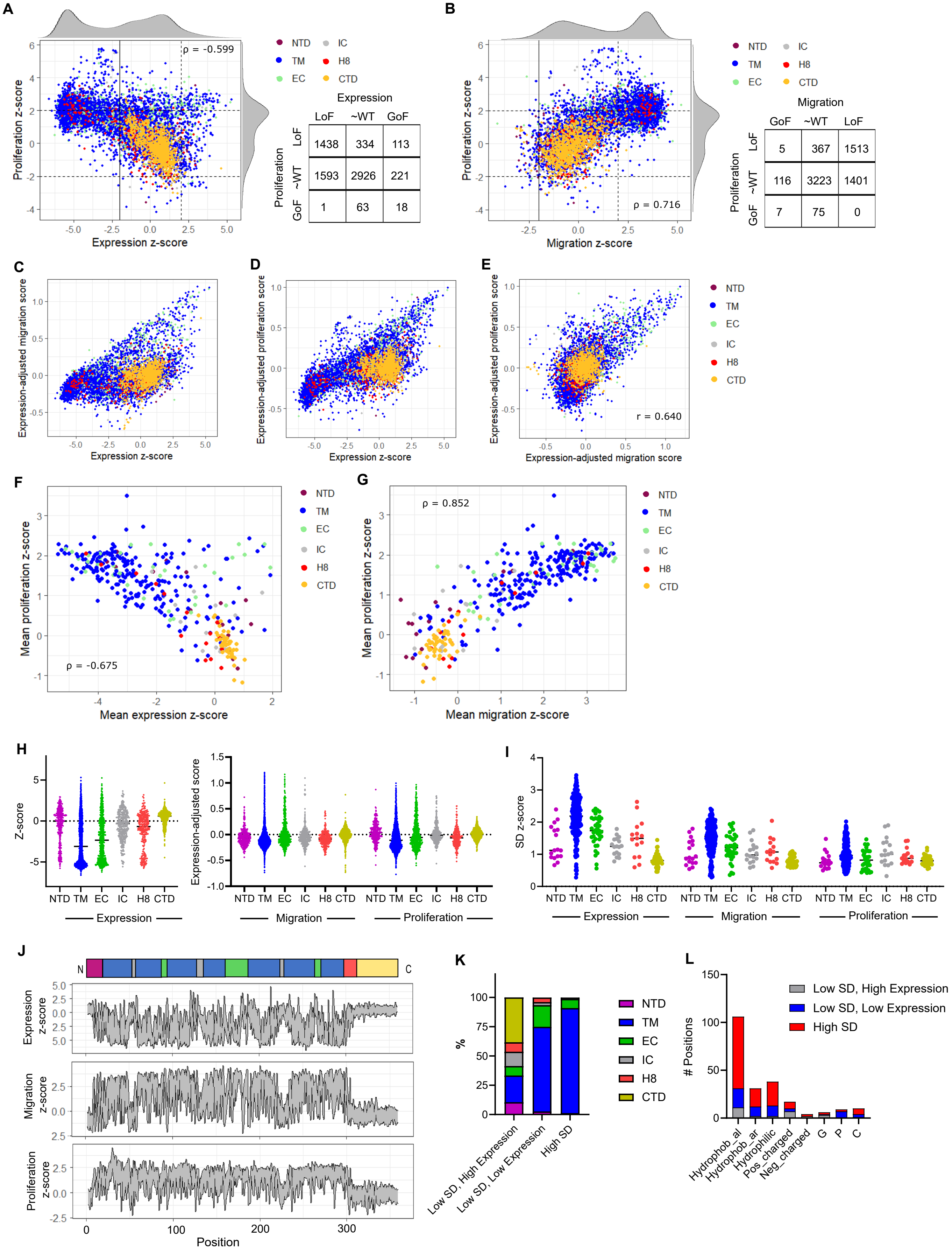
A
ρ = -0.599
Proliferation z-score
Expression z-score
NTD
TM
EC
IC
H8
CTD
Expression
LoF ~WT GoF
Proliferation
LoF 1438 334 113
~WT 1593 2926 221
GoF 1 63 18
B
ρ = 0.716
Proliferation z-score
Migration z-score
Migration
GoF ~WT LoF
Proliferation
LoF 5 367 1513
~WT 116 3223 1401
GoF 7 75 0
C
Expression-adjusted migration score
Expression z-score
D
Expression-adjusted proliferation score
Expression z-score
E
Expression-adjusted proliferation score
Expression-adjusted migration score
r = 0.640
F
ρ = -0.675
Mean proliferation z-score
Mean expression z-score
G
ρ = 0.852
Mean proliferation z-score
Mean migration z-score
H
Z-score
Expression-adjusted score
NTD TM EC IC H8 CTD
Expression
Migration
Proliferation
I
SD z-score
Expression
Migration
Proliferation
J
N
C
Expression z-score
Migration z-score
Proliferation z-score
Position
K
%
Low SD, High Expression
Low SD, Low Expression
High SD
L
Positions
Low SD, High Expression
Low SD, Low Expression
High SD
Hydrophob_al
Hydrophob_ar
Hydrophilic
Pos_charged
Neg_charged
G
P
C

Figure S2: Heterogeneity of P2RY8 DMS results, related to Figure 1. (A) Plot comparing expression and proliferation z-scores of missense variants, colored by protein domain, with table showing variant count by section, boundaries at z-scores of 2 and -2. **(B)** Plot comparing migration and proliferation z-scores of missense variants, colored by protein domain, with table showing variant count by section, boundaries at z-scores of 2 and -2. **(C)** Plot comparing expression z-scores and expression-adjusted migration scores of missense variants, colored by protein domain**. (D)** Plot comparing expression z-scores and expression-adjusted proliferation scores of missense variants, colored by protein domain**. (E)** Plot comparing expression-adjusted migration and expression-adjusted proliferation scores of missense variants, colored by protein domain**. (F)** Plot comparing mean expression and mean proliferation z-scores for each position (averaged across all missense variants at given position), colored by protein domain. **(G)** Plot comparing mean migration z-scores and mean proliferation z-scores for position (averaged across all missense variants at given position), colored by protein domain. **(H)** Variant expression z-scores, expression-adjusted migration scores, and expression-adjusted proliferation scores, partitioned by protein domain; lines mark medians; negative scores are LoF for expression, GoF for migration and proliferation. **(I)** Standard deviation of variant expression, migration, and proliferation z-scores within each position, partitioned by protein domain; lines mark medians. **(J)** Line plots depicting maximum and minimum variant z-scores at each position for expression, migration, and proliferation. **(K)** Distribution across protein domains of positions partitioned by missense variant expression mean and SD; threshold between low and high SD being SD of 2; threshold between low and high expression being mean expression z-score of -2. **(L)** Graph of number of TM positions with each expression score pattern, partitioned by WT amino acid; threshold between low and high SD being SD of 2; threshold between low and high expression being mean expression z-score of -2. Hydrophob_al = A, I, L, M, or V; Hydrophob_ar = F, W, or Y; Hydrophilic = N, S, T, or Y; Pos-charged = H, K, or R; Neg_charged = D or E; CTD, C-terminal domain; EC, extracellular loop; GoF, gain-of-function; H8, helix 8; IC, intracellular loop; LoF, loss-of-function; N, N-terminus; NTD, N-terminal domain; r, Pearson correlation coefficient; TM, transmembrane helices; ρ, Spearman correlation coefficient.

Figure S3

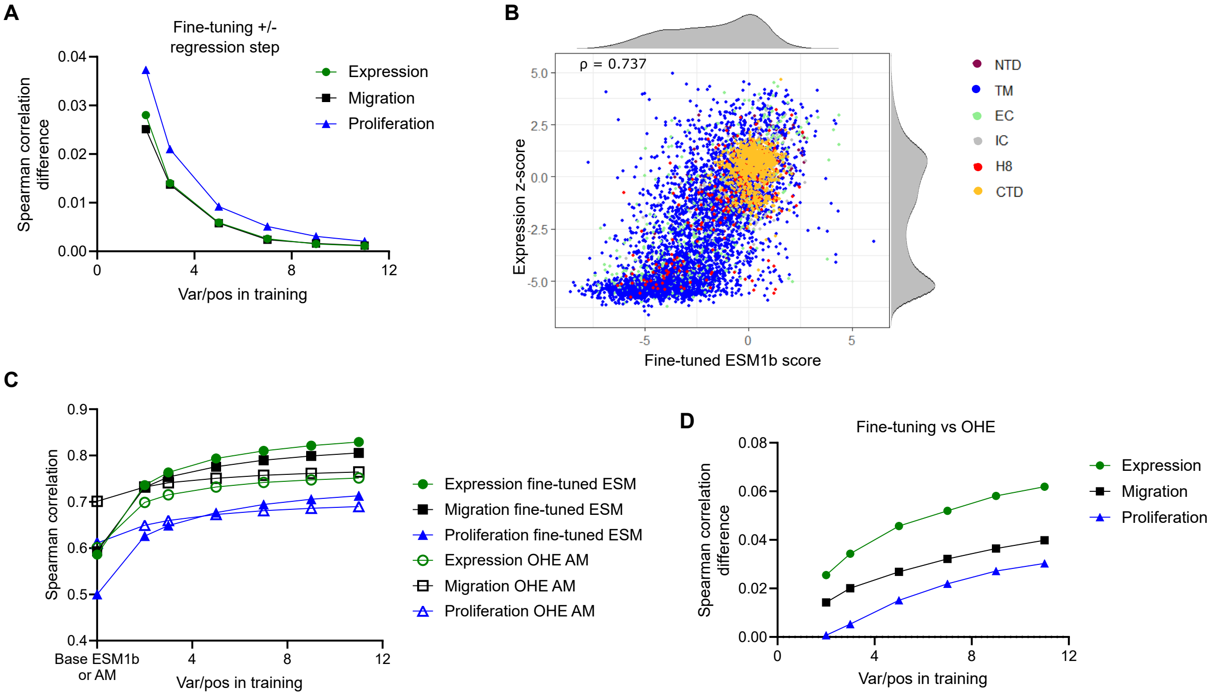

Figure S3: Supplemental ESM1b fine-tuning analyses, related to Figure 2. (A) Plot comparing mean Spearman correlations between DMS effect sizes and fine-tuned ESM1b with various training set sizes, with or without final ridge regression step (value > 0 indicates higher correlation with ridge regression). **(B)** Plot comparing representative 2 variant/position fine-tuned ESM1b and DMS expression z-score, variants colored by protein domain. **(C)** Plot comparing Spearman correlation between DMS z-scores and fine-tuned ESM1b or OHE AM scores as training set size varies. Shows mean and SD (k = 50). **(D)** Plot comparing difference in mean Spearman correlation at given training set size between DMS z-scores and fine-tuned ESM1b or OHE ESM1b (values > 0 indicate higher correlation with fine-tuning). CTD, C-terminal domain; EC, extracellular loop; GoF, gain-of-function; H8, helix 8; IC, intracellular loop; LoF, loss-of-function; NTD, N-terminal domain; OHE, one-hot encoded regression; TM, transmembrane helices; ρ, Spearman correlation coefficient.

Figure S4

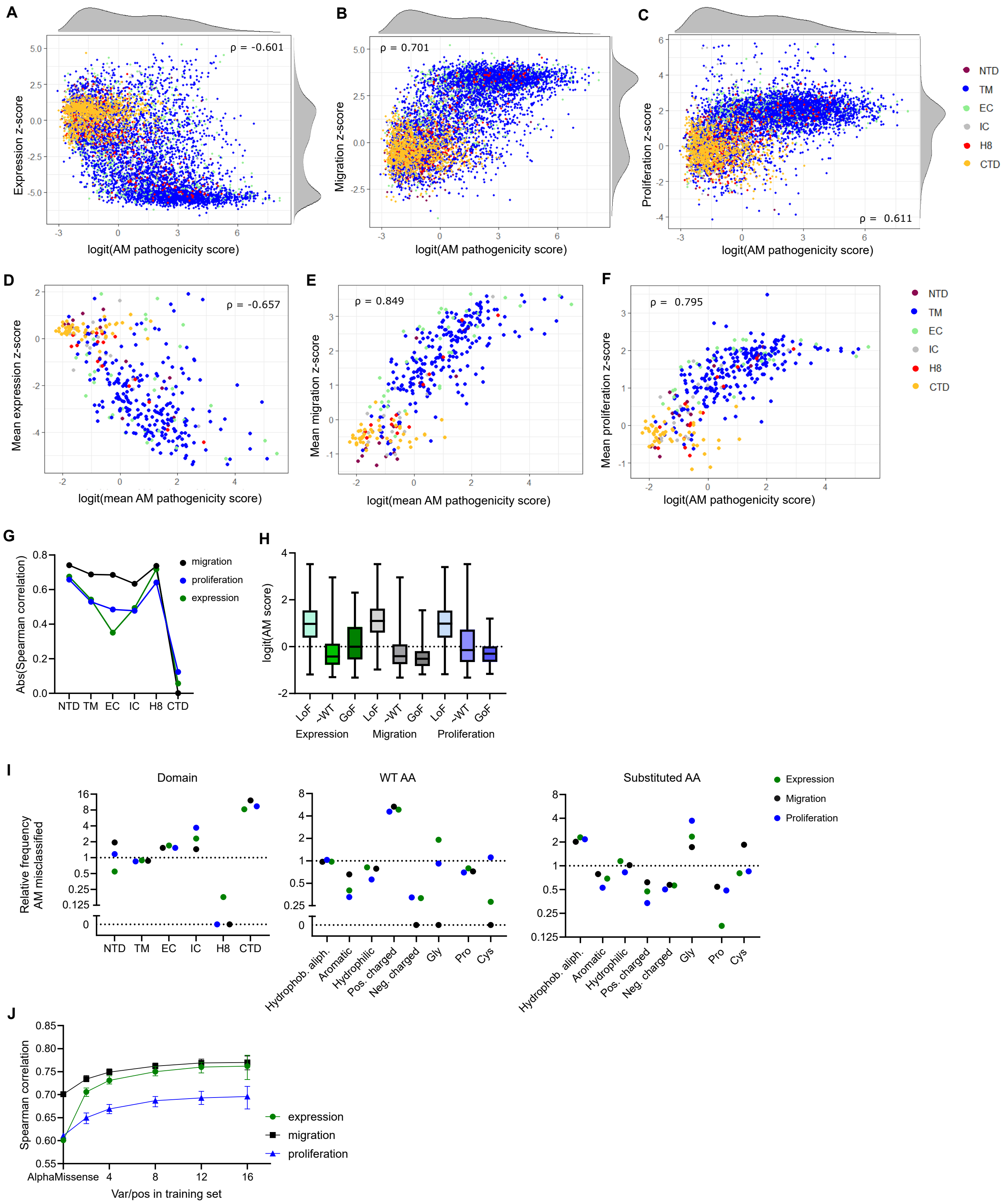

A
Expression z-score
logit(AM pathogenicity score)
ρ = -0.601
B
Migration z-score
logit(AM pathogenicity score)
ρ = 0.701
C
Proliferation z-score
logit(AM pathogenicity score)
ρ = 0.611
NTD
TM
EC
IC
H8
CTD
D
Mean expression z-score
logit(mean AM pathogenicity score)
ρ = -0.657
E
Mean migration z-score
logit(mean AM pathogenicity score)
ρ = 0.849
F
Mean proliferation z-score
logit(AM pathogenicity score)
ρ = 0.795
G
Abs(Spearman correlation)
migration
proliferation
expression
NTD TM EC IC H8 CTD
H
logit(AM score)
LoF ~WT GoF LoF ~WT GoF LoF ~WT GoF
Expression
Migration
Proliferation
I
Relative frequency AM misclassified
Domain
WT AA
Substituted AA
Expression
Migration
Proliferation
NTD TM EC IC H8 CTD
Hydrophob. aliph.
Aromatic
Hydrophilic
Pos. charged
Neg. charged
Gly
Pro
Cys
J
Spearman correlation
AlphaMissense
Var/pos in training set
expression
migration
proliferation

Figure S4: Improving AlphaMissense variant effect prediction using limited experimental data, related to Figure 2. (A-C) Plots comparing AM pathogenicity score and **(A)** expression z-score, **(B)** migration z-score, or **(C)** proliferation z-score for each variant, colored by protein domain. **(D-F)** Plots comparing mean AM pathogenicity score and **(D)** expression mean z-score, **(E)** migration mean z-score, or **(F)** proliferation mean z-score for each position, colored by protein domain. **(G)** Plot of Spearman correlation between logit-transformed AM pathogenicity score and variant expression, migration, and proliferation z-scores, variants partitioned by protein domain. **(H)** Plot comparing distribution of AM pathogenicity scores for each variant, partitioned by DMS phenotypes. Whiskers extend to maximum and minimum, boxes shows 25th percentile, median, and 75th percentile. **(I)** Plots showing proportion of deleterious variants (z-score < -2 for expression, > 2 for migration and proliferation) with benign AM pathogenicity scores (< 0.34) relative to proportion of deleterious variants with non-benign AM scores, as partitioned by protein domain (left), WT amino acid class (center), and substituted (variant) amino acid class (right). Hydrophobic aliphatic residues are A, I, L, M, and V; aromatic residues are F, W, and Y; hydrophilic residues are N, Q, S, and T; positively charged residues are H, K, and R; negatively charged residues are D and E. (**J)** Plot of Spearman correlation between OHE AM scores and DMS z-scores as training set size varies. Shows mean and SD (k = 50). AM, AlphaMissense; CTD, C-terminal domain; EC, extracellular loop; GoF, GoF, gain-of-function, z-score > 2 for expression, < -2 for migration, proliferation; LoF, loss-of-function, z-score < -2 for expression, > 2 for migration, proliferation; H8, helix 8; IC, intracellular loop; NTD, N-terminal domain; OHE, one-hot encoded regression, ρ, Spearman correlation coefficient.

Figure S5

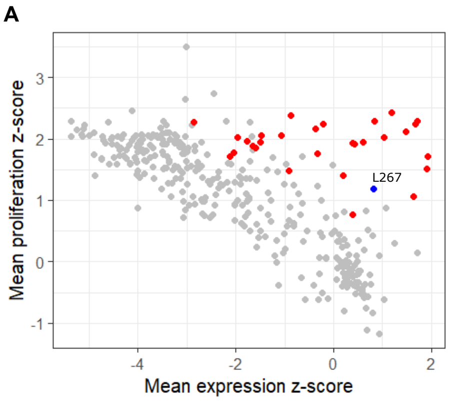

Figure S5: Proliferation phenotype of high expression-adjusted migration score positions, related to Figure 3. (A) Plot comparing expression and proliferation mean z-scores for each position, with those with high expression-adjusted migration scores (Fig. 3a) colored red; additional position with high expression-adjusted proliferation but not also expression-adjusted migration score colored blue.

Figure S6

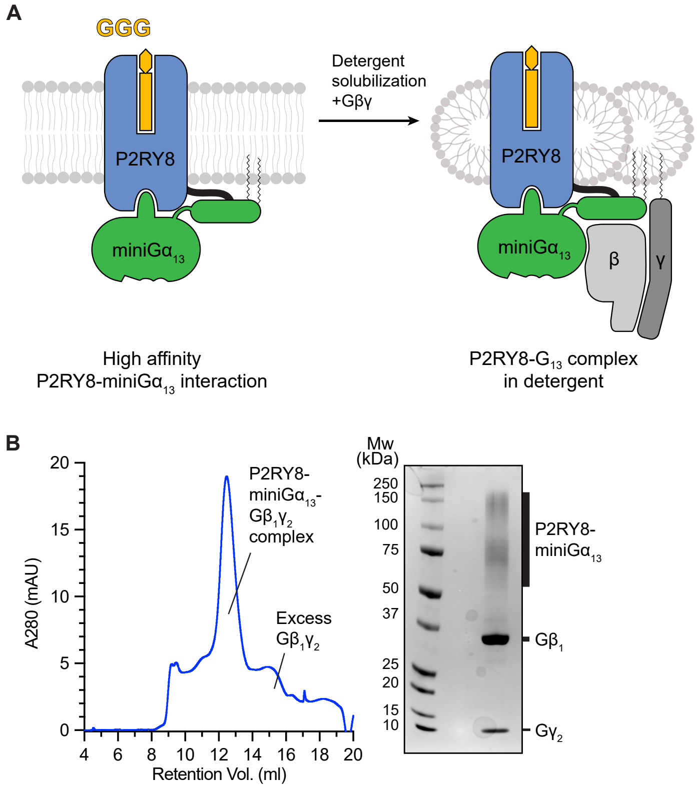

Figure S6: Biochemical preparation of P2RY8-G_{13} complex bound to GGG, related to Figure 4. (A) Schematic outlining strategy for stabilization and purification of P2RY8 bound to G_{13} and GGG. This diagram is modified from one previously published [S1]. **(B)** Size-exclusion chromatogram of purified P2RY8-G_{13} complex used for structure determination together with representative SDS-PAGE gel analysis of the collected fraction containing the P2RY8-G_{13} complex.

Figure S7

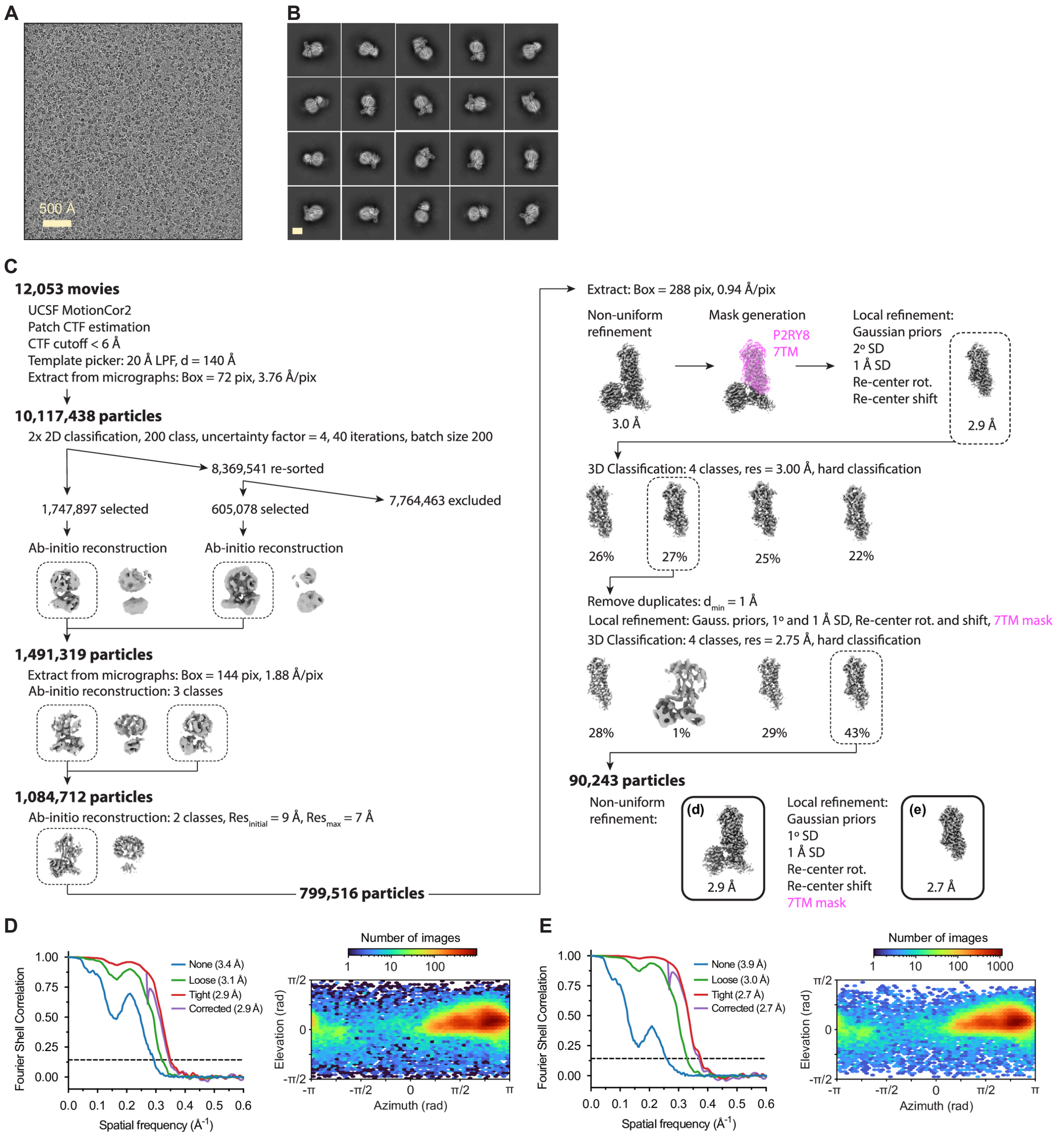

Figure S7: Cryo-EM data processing for P2RY8-G13, related to Figure 4. (A) Representative cryo-electron micrograph from the curated P2RY8-G13 data set (n = 12,053 obtained from a Titan Krios microscope). **(B)** A subset of highly populated, reference-free 2D-class averages are shown. Scale bar is 50 Å. **(C)** Schematic showing the image processing workflow for P2RY8-G13. Initial processing performed using UCSF MotionCor2 and cryoSPARC, where particles were sorted using a combination of 2D classification, ab-inition reconstruction, and 3D classification. Finally, EM maps were obtained in cryoSPARC using the non-uniform and local refinement tools. Dashed boxes indicated selected classes, and 3D volumes of classes and refinements are shown along with the global gold-standard Fourier shell correlation (GSFSC) resolutions. **(D,E)** Map validation for the P2RY8-G13, **(D)** globally refined, and **(E)** locally refined cryo-EM maps. GSFSC curves are calculated in cryoSPARC. Euler angle distributions calculated in cryoSPARC are also provided for each map.

Figure S8

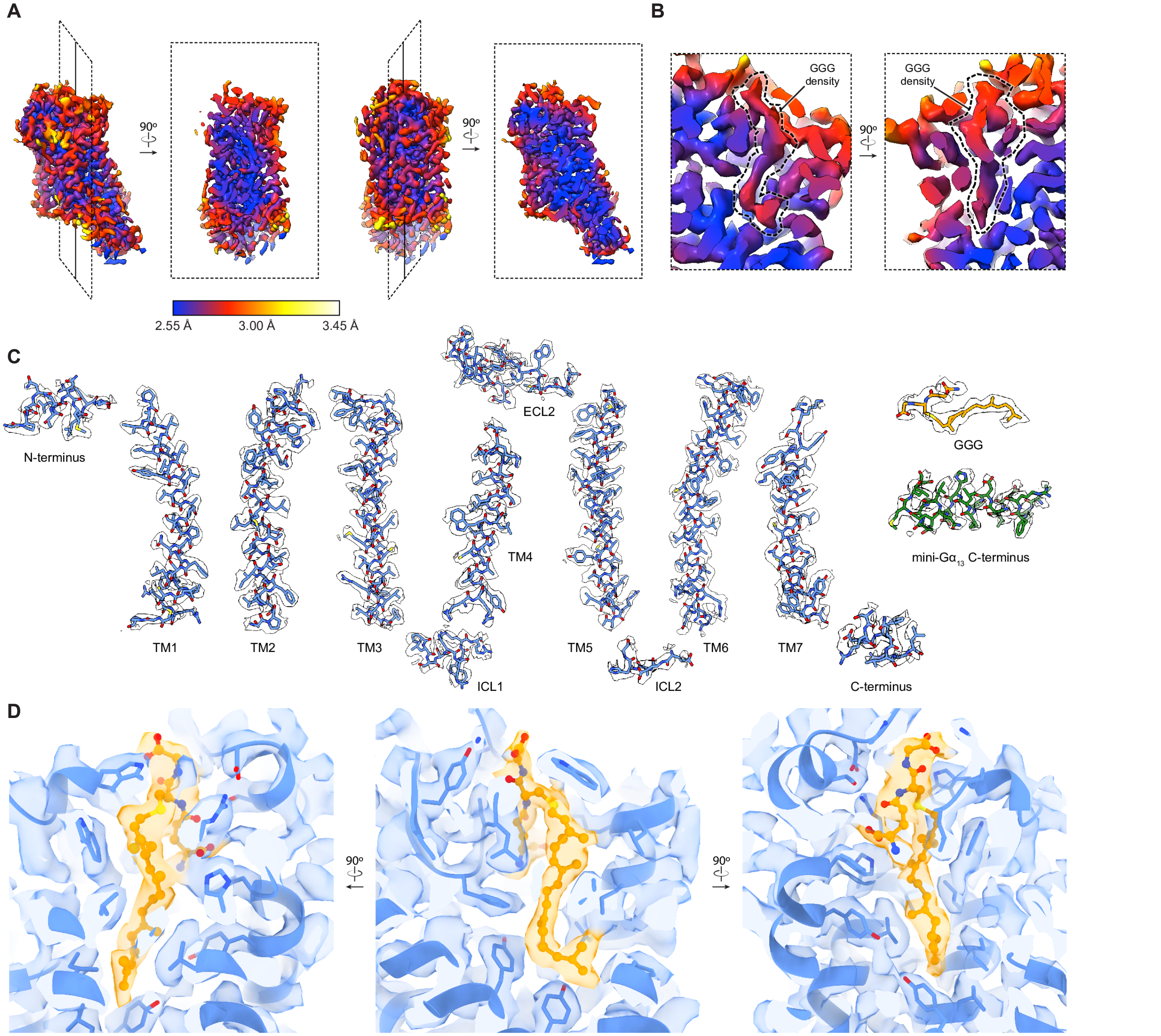

Figure S8: Cryo-EM density and atomic model, related to Figures 4 and 5. (A) Orthogonal views of local resolution for the locally refined map covering the 7TM domain of the P2RY8-G13 complex, calculated with the local resolution estimation tool in cryoSPARC. **(B)** Close-up view showing the local resolution of the GGG binding site. **(C)** Representative cryo-EM densities from the 3D reconstruction of P2RY8 from a sharpened, locally refined map of P2RY8-G13 at a map threshold of 1.02. Shown are the transmembrane helices and loop regions of P2RY8, the C-terminal helix of mini-Gα13, as well as GGG. **(D)** Close-up views of cryo-EM density supporting GGG binding pose (orange sticks and density) using a sharpened, locally refined map of P2RY8-G13 at a map threshold of 1.02.

Figure S9

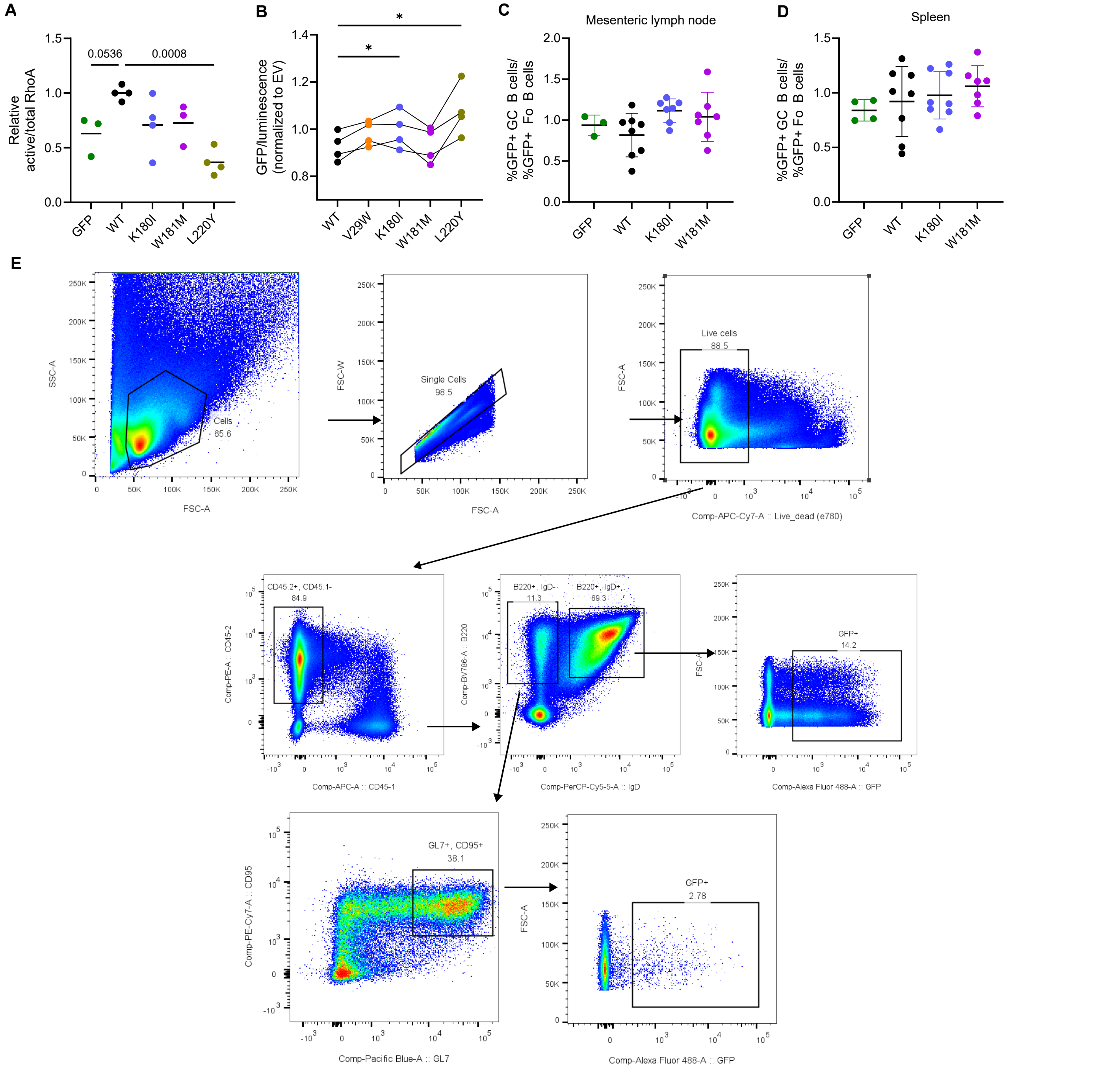

Figure S9: In vivo variant analysis in spleen and mesenteric lymph node, related to Figure 6. (A) Transduced Ly8 cells treated with GGG, lysed, and aliquots assayed for total RhoA and active (GTP-bound) RhoA by ELISA, plot depicts ratio of active to total RhoA signal, normalized within each experiment to WT P2RY8. Pooled results from 3 experiments, each point is a biological replicate. One-way ANOVA with Dunnett's multiple comparison's test with comparisons to WT. **(B)** BRET assay: 293T cells, co-transfected with P2RY8 variant, RLuc-Gα13 fusion, Gβ, and Gγ-GFP fusion, baseline ratio of luminescence and GFP fluorescence determined, ratio normalized to GFP only (EV). Each point is a biological replicate. Results from 4 experiments with line connecting variant results from same experiment. RM one-way ANOVA withnGeisser-Greenhouse correction, comparison to WT with Dunnett's multiple comparisons adjustment. * adjusted p-value of 0.01-0.05. **(C,D)** Irradiated CD45.1 mice were reconstituted with bone marrow transduced with EV-GFP, WT-P2RY8-GFP, K180I-GFP, or W181M-GFP. After reconstitution, mesenteric lymph nodes **(C)** and spleen **(D)** were analyzed for the frequency of GFP+ cells among GC and follicular B cells and the ratio plotted. Pooled from 3 experiments, each point is a mouse. Graphs show means and SDs. **(E)** Representative flow cytometry gating; this depicts this depicts a Peyer's patch sample. GC B cells identified as singlet live cells, B220+, IgD-, GL7+, CD95+; follicular B cells identified as singlet live cells, B220+, IgD+.

Figure S10

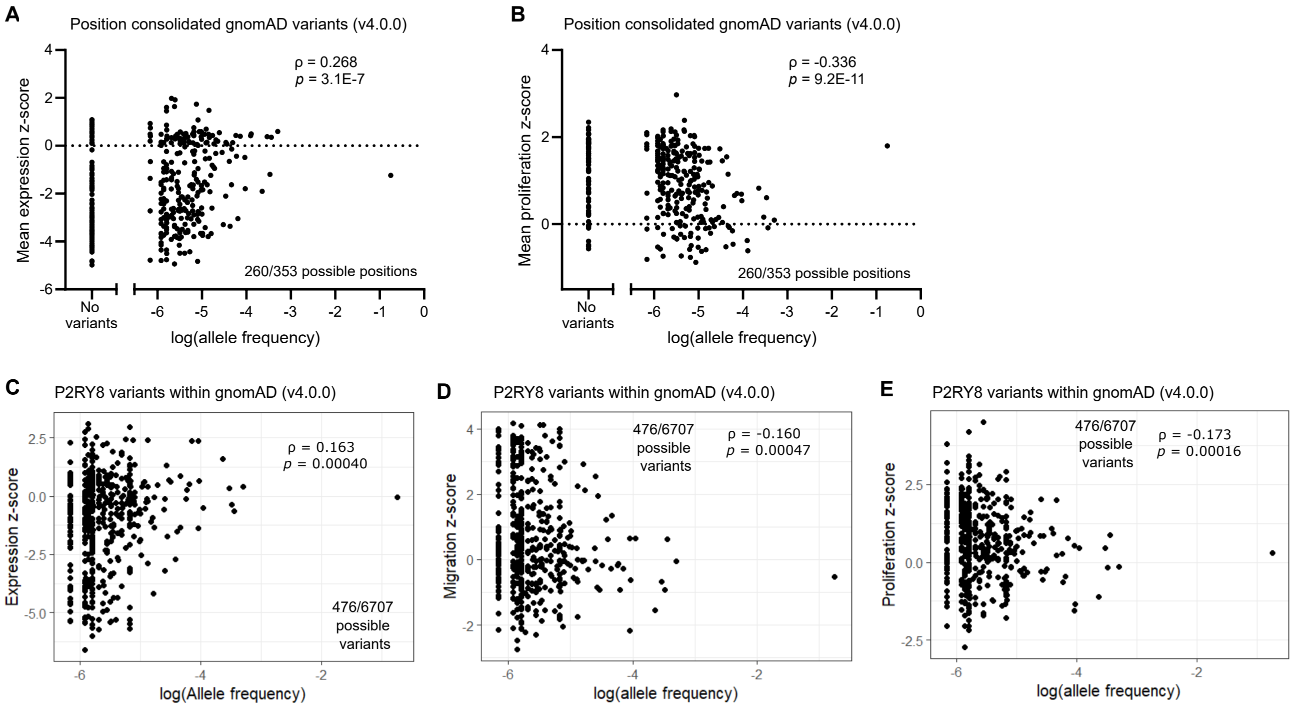

F

Expression

	gnomAD	DLBCL/BL	Non-hem COSMIC
All var	D=0.2039 p=2.22E-16	D=0.3223 p=1.51E-4	D=0.2222 p=5.81E-7
Non-hem COSMIC	D=0.153 p=0.0081	D=0.534 p=6.69E-10	
DLBCL/BL	D=0.5205 p=2.76E-10		

Migration

	gnomAD	DLBCL/BL	Non-hem COSMIC
All var	D=0.2027 p=4.44E-16	D=0.3795 p=3.87E-6	D=0.2316 p=1.59E-7
Non-hem COSMIC	D=0.1963 p=2.39E-4	D=0.5839 p=7.31E-12	
DLBCL/BL	D=0.5724 p=2.37E-12		

Proliferation

	gnomAD	DLBCL/BL	Non-hem COSMIC
All var	D=0.2141 p=2.2E-16	D=0.2971 p=6.29E-4	D=0.1688 p=3.36E-4
Non-hem COSMIC	D=0.0642 p=0.719	D=0.4596 p=2.42E-7	
DLBCL/BL	D=0.5023 p=1.31E-9		

Figure S10: Additional germline and lymphoma-associated P2RY8 variant analysis, related to Figure 7.
(A,B) Plot comparing summed allele frequency for all missense variants within each position in gnomAD (v4.0.0) to **(A)** mean expression z-score or **(B)** mean proliferation z-score for each position. **(C-E),** Plot comparing missense variant allele frequency in gnomAD (v4.0.0) to the **(C)** expression, **(D)** migration, or **(E)** proliferation z-scores for those variants. **(F)** Tables of Kolmogorov-Smirnov test results comparing expression, migration, and proliferation score distributions for all missense variants, variants present in gnomAD (v4.0.0), non-hematologic cancer variants from COSMIC, and set of DLBCL and Burkitt lymphoma variants. Asymptotic two-sample Kolmogorov-Smirnov test performed; p-values not adjusted for multiple comparison. **(A-E)** ρ, Spearman correlation; p-value calculated using algorithm AS 89 with Edgeworth series approximation.

Table S3. Cryo-EM data collection, refinement, and validation statistics, related to Figure 4

	**GGG-bound P2RY8-G_{13}**
EMDB: Full map	EMD-47912
EMDB: 7TM map	EMD-47914
RCSB PDB: Model	9ECJ
**Data collection**	
Microscope	Thermo Scientific Krios G3i
Detector	Thermo Scientific Falcon 4i with Selectris X energy filter
Voltage (kV)	300
Magnification	130,000
Defocus range (µm)	-0.8 to -2.1
Pixel size, physical (Å)	0.94
Total exposure (e^-/Å^2)	50
Images, number of	12,053
EER fractions	80
Initial particles, number of	10,117,438
Final particles, number of	90,243
Symmetry imposed	C1
Map sharpening, *B* factor (Å^2)	
Full map	-79.5
7TM map	-83.0
Map resolution, masked (Å)	
Full map	2.9
7TM map	2.7
FSC threshold	0.143
**Refinement**	
Initial model used (AlphaFold code)	Q86VZ1
Model resolution (Å)	3.4
FSC threshold	0.5
Model composition	
Chains	4
Non-hydrogen atoms	6,323
Protein residues	790
Ligands	1
B factors (Å^2)	
Protein	35.24
Ligand	34.49
R.m.s. deviations	
Bond length (Å)	0.013
Bond angles (°)	2.006
Validation	
MolProbity score	1.67
Clash score	6.34
Rotamer outliers (%)	0.29
Ramachandran plot	
Favored (%)	95.38
Allowed (%)	4.62
Disallowed (%)	0.00

Supplemental References:

S1. Rasmussen, S.G.F., DeVree, B.T., Zou, Y., Kruse, A.C., Chung, K.Y., Kobilka, T.S., Thian, F.S., Chae, P.S., Pardon, E., Calinski, D., et al. (2011). Crystal structure of the β2 adrenergic receptor-Gs protein complex. Nature *477*, 549–555. https://doi.org/10.1038/nature10361.
