# Supplementary material for: Phenotypic pleiotropy of missense variants in human B cell confinement receptor P2RY8
Source: Cell Genom. 2025 Sep 9;5(11):100981. doi: 10.1016/j.xgen.2025.100981 (PMC12648108; doi:10.1016/j.xgen.2025.100981)
Supplement: Document S2. Article plus supplemental information [file mmc5.pdf]

Cell Genomics

Article

Phenotypic pleiotropy of missense variants in human B cell confinement receptor P2RY8

Graphical abstract

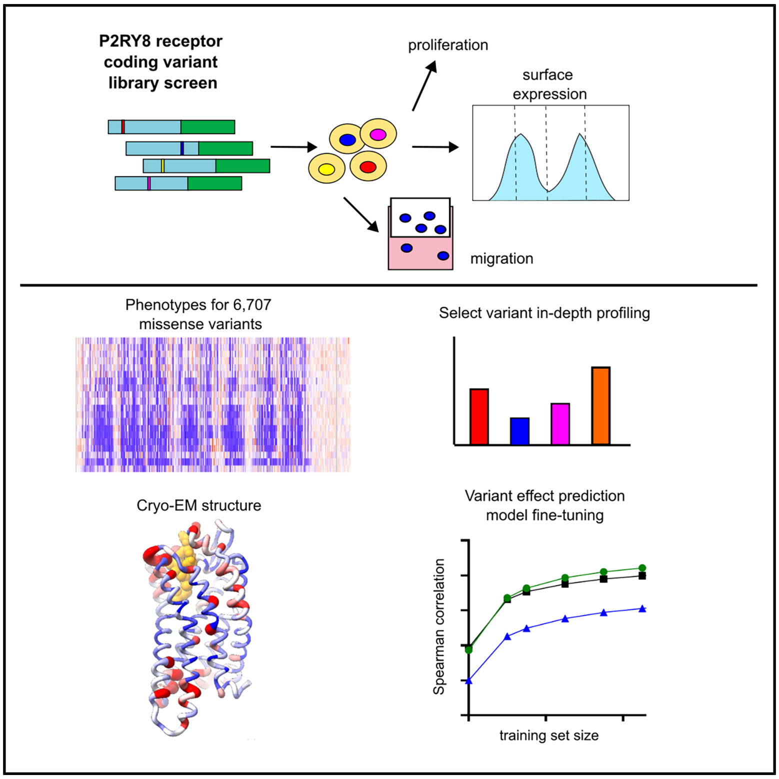

Authors

Taylor N. LaFlam, Christian B. Billesbølle, Tuan Dinh, ..., Aashish Manglik, Jason G. Cyster, Chun Jimmie Ye

Correspondence

taylor.laflam@ucsf.edu (T.N.L.),
jason.cyster@ucsf.edu (J.G.C.),
jimmie.ye@ucsf.edu (C.J.Y.)

In brief

LaFlam et al. combine deep mutational scanning, structural biology, and machine learning to characterize how missense variants affect the expression and function of the immune GPCR P2RY8. Their findings reveal pleiotropy and highlight how sparse experimental data can improve computational variant effect predictions.

Highlights

- Deep mutational scanning of P2RY8 maps variant effects across expression and function
- Cryo-EM reveals active structure of P2RY8 bound to endogenous ligand GGG
- DMS results enhance accuracy of computational variant effect predictions
- Variants show pleiotropy with distinct effects possible on migration and proliferation

LaFlam et al., 2025, Cell Genomics *5*, 100981
November 12, 2025 © 2025 The Authors. Published by Elsevier Inc.
https://doi.org/10.1016/j.xgen.2025.100981

CellPress

Cell Genomics

CellPress
OPEN ACCESS

Article

Phenotypic pleiotropy of missense variants in human B cell confinement receptor P2RY8

Taylor N. LaFlam,[1,2,3,18,22,*] Christian B. Billesbølle,[4,18] Tuan Dinh,[5] Finn D. Wolfreys,[2,6,19] Erick Lu,[2,6,20] Tomas Matteson,[7,8,21] Jinping An,[2,6] Ying Xu,[2,6] Arushi Singhal,[3] Nadav Brandes,[9] Vasilis Ntranos,[5,8,10,11,12] Aashish Manglik,[4,13,14,15] Jason G. Cyster,[2,6,*] and Chun Jimmie Ye[3,5,7,8,10,11,16,17,*]

[1]Division of Pediatric Rheumatology, Department of Pediatrics, University of California, San Francisco, San Francisco, CA, USA
[2]Department of Microbiology and Immunology, University of California, San Francisco, San Francisco, CA, USA
[3]Gladstone-UCSF Institute of Genomic Immunology, San Francisco, CA, USA
[4]Department of Pharmaceutical Chemistry, University of California, San Francisco, San Francisco, CA, USA
[5]Department of Epidemiology and Biostatistics, University of California, San Francisco, San Francisco, CA, USA
[6]Howard Hughes Medical Institute, University of California, San Francisco, San Francisco, CA, USA
[7]Division of Rheumatology, Department of Medicine, University of California, San Francisco, San Francisco, CA, USA
[8]Institute for Human Genetics, University of California, San Francisco, San Francisco, CA, USA
[9]Department of Biochemistry and Molecular Pharmacology, New York University, New York, NY, USA
[10]Department of Bioengineering and Therapeutic Sciences, University of California, San Francisco, San Francisco, CA, USA
[11]Bakar Computational Health Sciences Institute, University of California, San Francisco, San Francisco, CA, USA
[12]Diabetes Center, University of California, San Francisco, San Francisco, CA, USA
[13]Chan Zuckerberg Biohub, San Francisco, CA, USA
[14]Quantitative Biosciences Institute, San Francisco, CA, USA
[15]Department of Anesthesia and Perioperative Care, University of California, San Francisco, San Francisco, CA, USA
[16]Parker Institute for Cancer Immunotherapy, University of California, San Francisco, San Francisco, CA, USA
[17]Arc Institute, Palo Alto, CA, USA
[18]These authors contributed equally
[19]Present address: Department of Ophthalmology, University of California, San Francisco, San Francisco, CA, USA
[20]Present address: Gilead Sciences, Foster City, CA, USA
[21]Present address: Retro Biosciences, San Francisco, CA, USA
[22]Lead contact
*Correspondence: taylor.laflam@ucsf.edu (T.N.L.), jason.cyster@ucsf.edu (J.G.C.), jimmie.ye@ucsf.edu (C.J.Y.)
https://doi.org/10.1016/j.xgen.2025.100981

SUMMARY

Missense variants can have pleiotropic effects on protein function, and predicting these effects can be difficult. We performed near-saturation deep mutational scanning of P2RY8, a G protein-coupled receptor that promotes germinal center B cell confinement. We assayed the effect of each variant on surface expression, migration, and proliferation. We delineated variants that affected both expression and function, affected function independently of expression, and discrepantly affected migration and proliferation. We also used cryo-electron microscopy to determine the structure of activated, ligand-bound P2RY8, providing structural insights into the effects of variants on ligand binding and signal transmission. We applied the deep mutational scanning results to both improve computational variant effect predictions and to characterize the phenotype of germline variants and lymphoma-associated variants. Together, our results demonstrate the power of integrating deep mutational scanning, structure determination, and *in silico* prediction to advance the understanding of a receptor important in human health.

INTRODUCTION

Missense variants, in which the amino acid sequence of a protein is altered, can significantly impact protein function. Germline missense variants are frequent in humans,[1,2] highly enriched for those causal for rare Mendelian disorders, and statistically associated with common complex diseases.[3] Somatic missense mutations are frequent in cancers and major contributors to tumorigenesis.[4,5] Determining the effects of missense variants on protein function is essential for diagnosing and understanding the genetic causes of human diseases.[2,6]

Current approaches to missense variant annotation rely on both computational and experimental methods. Emerging variant effect prediction (VEP) approaches using deep learning can predict the effects of all missense mutations and have demonstrated success in identifying variants that cause Mendelian diseases, most of which are loss of function (LoF).[6–8] However, these algorithms are much less effective at identifying cancer driver mutations, which are frequently gain of function (GoF)

This is an open access article under the CC BY license (http://creativecommons.org/licenses/by/4.0/).

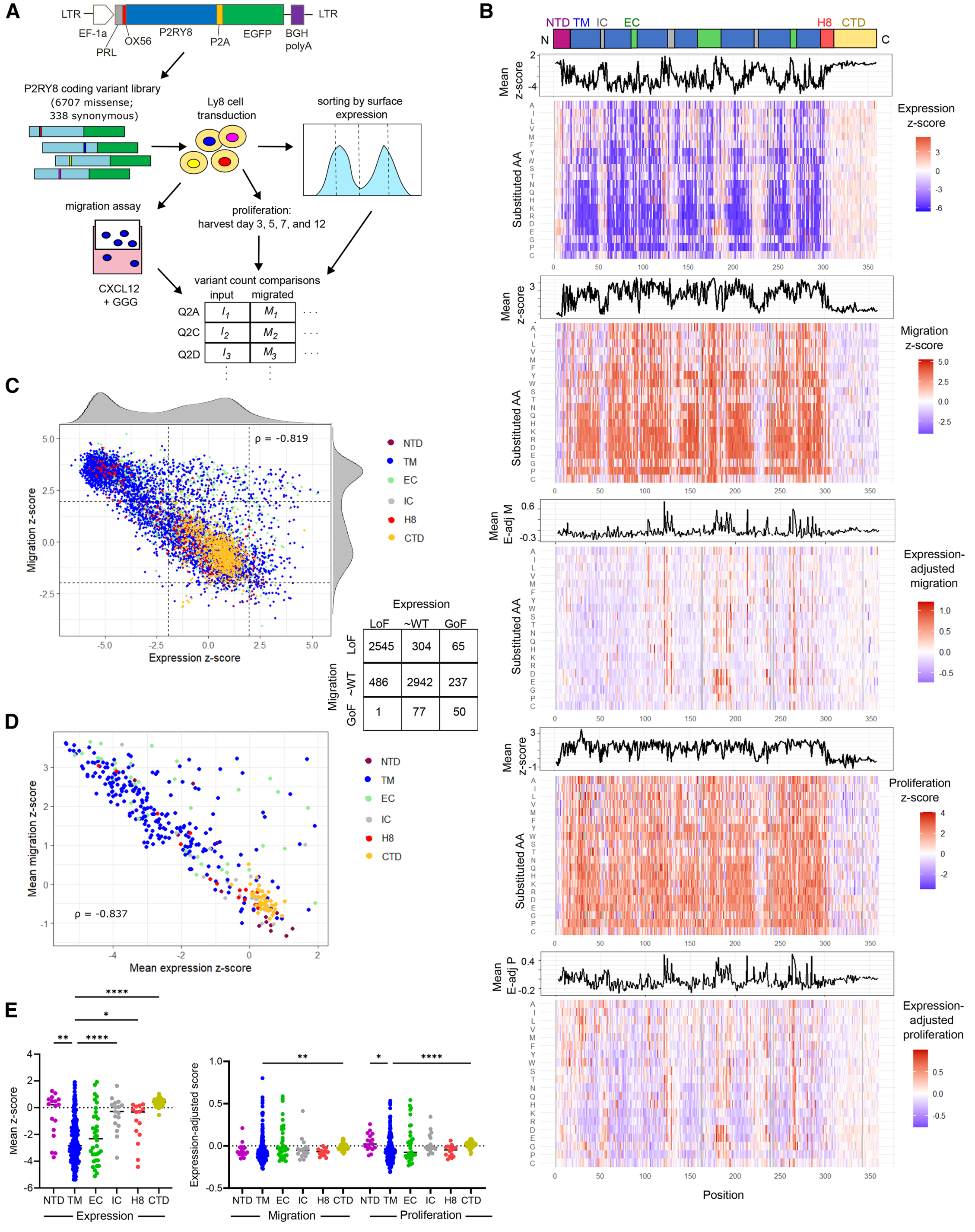

Figure 1. DMS of P2RY8 across three phenotypes

(A) Lentiviral vector schematic and DMS approach. OX56 is a peptide tag,[33] and PRL is a preprolactin signal peptide.

(B) Heatmaps of surface expression, migration, and proliferation *Z* scores; expression-adjusted migration and expression-adjusted proliferation scores; and accompanying line plots of mean scores for each position.

(C) Plot comparing missense variant expression and migration *Z* scores colored by domain, table of variant counts, and boundaries at *Z* scores of 2 and −2.

(D) Plot comparing mean expression and mean migration *Z* scores (averaged across all missense variants at each position), colored by domain.

(legend continued on next page)

and not evolutionarily conserved.[9,10] Moreover, these tools provide only a single functional prediction per variant, limiting their capacity to predict effects across diverse phenotypes.

In parallel, the convergence of next-generation sequencing and massively parallel gene synthesis have propelled experimental profiling of large numbers of missense variants via deep mutational scanning (DMS), also known as multiplexed assays of variant effect (MAVE).[11–13] Unlike computational predictions, DMS experiments can assess variant effects across multiple phenotypes and cell types, offering direct data on functional consequences. Furthermore, because DMS does not depend on evolutionary conservation, it provides an unbiased approach to variant characterization. DMS approaches remain low throughput, and most human genes have yet to be profiled. Nevertheless, there is a growing body of DMS data, including an international collaborative effort to collate these results[14] that could ultimately inform and refine computational VEP tools.

One application of these approaches is studying G protein-coupled receptors (GPCRs), a large family of ~800 transmembrane receptors that play important roles across numerous physiologic pathways and are the most frequent drug targets in medicine.[15,16] Transducers of GPCR signaling include heterotrimeric G proteins and β-arrestins.[17,18] Several GPCRs have been the focus of DMS, providing insights into how missense variants affect their surface expression[19–21] and signaling.[22,23] Examining both expression and function allows one to distinguish between effects mediated through, or independent of, changes in expression.[23] Despite this progress, GPCRs that signal primarily through $G_{12/13}$ remain underexplored among DMS and structural studies.[24]

P2RY8 is a G_{13}-coupled GPCR that is expressed on several lymphocyte subsets, including germinal center (GC) B cells, where it restrains cell migration and proliferation.[25–27] Its ligand is S-geranylgeranyl-L-glutathione (GGG).[28] Addition of GGG in migration assays of P2RY8-expressing cells causes activation of RhoA and inhibition of migration toward chemokines.[29] The *P2RY8* locus is located on the pseudoautosomal region of the X and Y chromosomes and is frequently mutated in diffuse large B cell lymphoma (DLBCL) and Burkitt lymphoma.[25,30,31] In addition, variants in P2RY8 have been identified in a small number of patients with lupus, and these variants were found to affect B cell negative selection and plasma cell development when expressed in mice.[32]

The immunologic importance of P2RY8 and its restricted expression make it an attractive target for therapeutic intervention, but the current understanding of how specific missense variants affect its function is limited. To address this, we conducted DMS of P2RY8, evaluating surface expression and two functional outcomes: inhibition of migration and restraint of proliferation. Using cryo-electron microscopy (cryo-EM), we determined the structure of P2RY8 in complex with its ligand, GGG, providing a structural scaffold for interpreting how missense variants influence its active conformation and signaling. We also illustrated how integrating relatively sparse experimental data from these screens can enhance the performance of computational VEP tools. We performed in-depth validation of select variants, which provided further insights into P2RY8 function, including evidence that receptor initiation of pathways regulating migration and proliferation is at least partly distinct. In sum, we demonstrate the utility of performing DMS on multiple phenotypes and the complementary benefits of a multimodal approach to annotate the pleiotropic effects of missense variants of a GPCR to better understand its biology.

RESULTS

DMS of P2RY8 across three phenotypes

We performed DMS of P2RY8, using a pooled lentiviral library of 7,045 variants covering all possible substitutions for 353 of 358 non-start positions and synonymous variants for 338 positions (Table S1). We transduced OCI-Ly8 (Ly8) cells, a human DLBCL cell line, in which we previously knocked out endogenous P2RY8,[28] and performed three parallel screens: receptor surface expression (using the OX56 N-terminal tag[33]), cell migration, and proliferation (Figures 1A and S1A). Each of these three screens was highly reproducible across four replicates, with pairwise Pearson correlation coefficients (r) ranging from 0.96 to 0.97 for surface expression, 0.71 to 0.85 for migration, and 0.33 to 0.66 for proliferation (Figures S1B–S1D).

Missense variants showed a bimodal distribution of effect sizes (*Z* scores based on synonymous variant effects) across all three assays (Figures 1B, 1C, S2A, and S2B; Table S2). Approximately half of the missense variants reduced the expression of surface P2RY8, but 352 variants increased expression (Figure 1C), suggesting that GoF effects are detectable in our screens. As expected, the effects of missense variants on expression of P2RY8 are inversely correlated with their effects on migration (Figure 1C; Spearman correlation coefficient [ρ] of −0.819) and proliferation (Figure S2A; ρ = −0.599). The migration and proliferation effect sizes were also significantly correlated (Figure S2B; ρ = 0.716). To better identify variants with effects on migration or proliferation independent of the effect on expression, we also calculated expression-adjusted migration and expression-adjusted proliferation scores for each variant (Figures 1B, S2C, and S2D). Consistent with what was observed with unadjusted migration and proliferation effect sizes, these expression-adjusted scores were highly correlated with each other (Figure S2E; r = 0.640). Plotting the expression-adjusted migration score by position across the protein sequence showed several peaks (Figure 1B).

Averaging the effect sizes across substitutions for each position resulted in higher inverse correlation between migration and expression while also identifying several positions in which missense variants consistently affected function but not

(E) Mean expression *Z* scores, mean expression-adjusted migration scores, and mean expression-adjusted proliferation scores for each position, partitioned by domain; lines are medians. Negative scores are LoF for expression, GoF for migration or proliferation. Welch ANOVA test with Dunnett's T3 multiple comparisons test; for each assay TM compared to the five other domains; adjusted *$p < 0.05$, **$p < 0.01$, ***$p < 0.001$, ****$p < 0.0001$.

C, C terminus; CTD, C-terminal domain; EC, extracellular; GoF, gain of function; H8, helix 8; IC, intracellular; LoF, loss of function; N, N terminus; NTD, N-terminal domain; TM, transmembrane; ρ, Spearman correlation coefficient. See also Figures S1 and S2.

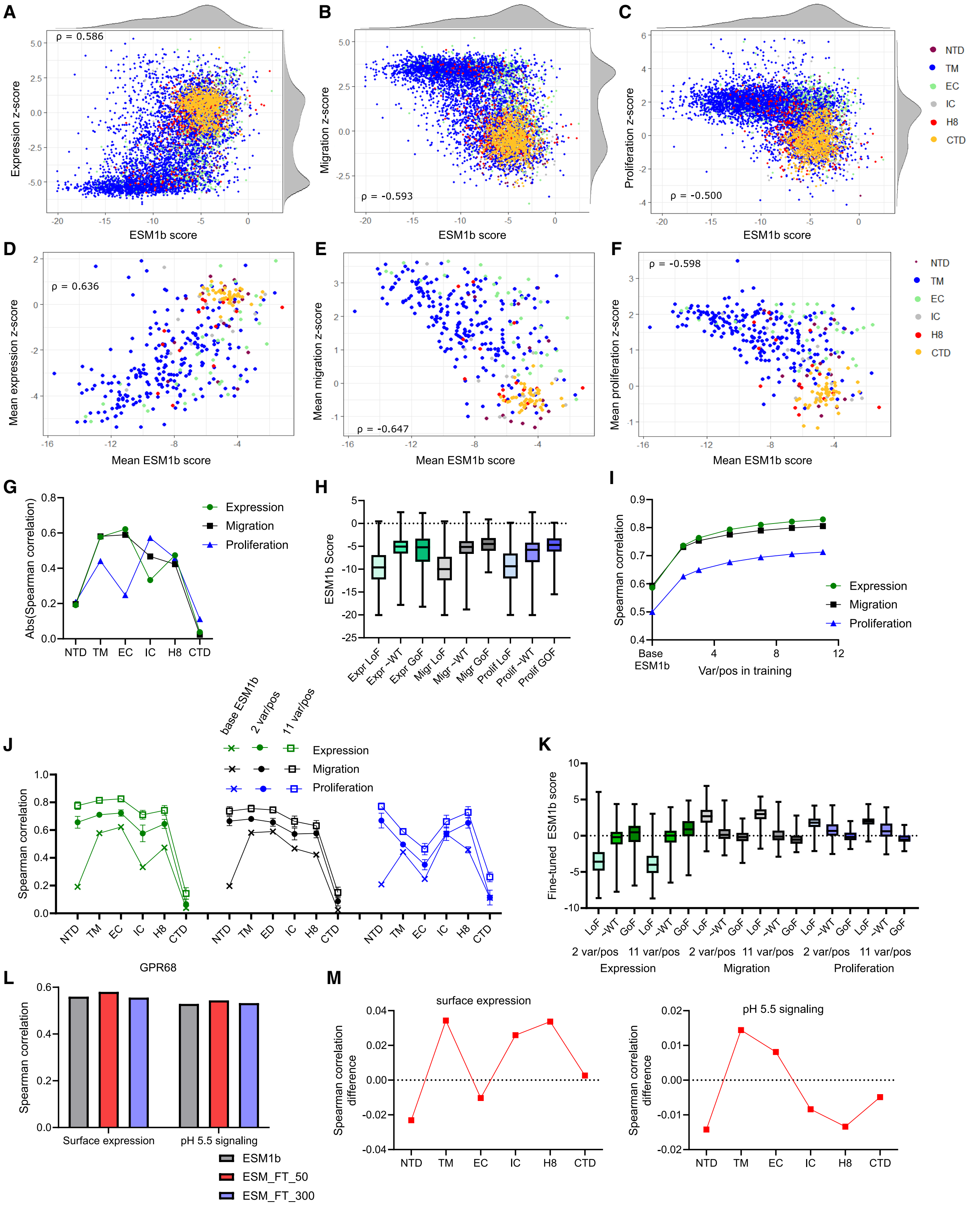

(legend on next page)

CellPress
OPEN ACCESS

expression (Figures 1D, S2F, and S2G). Mutation tolerance at specific positions varied significantly within each protein domain; highly sensitive positions were enriched in the transmembrane (TM) helices and extracellular (EC) loops, whereas no position in the C-terminal domain (CTD) was highly sensitive (Figure 1E). Similar differences between domains were also observed when examining the effect of individual variants, though large effect substitutions do exist in all domains (Figure S2H). The range of variant effects within positions varied widely, with some positions having similar effects for all missense variants and others having a large spread of effects (Figures S2I and S2J). Further examination showed that highly heterogeneous variant effects were concentrated in the TM helices (Figure S2K), with some tendencies also observed depending on the amino acid of a position (Figure S2L).

Improving computational VEP using limited experimental data

ESM1b is a self-supervised protein language model that has been shown recently to have high zero-shot predictive power of pathogenicity of coding variants.[6] We found that our DMS expression effect sizes correlated well overall with the variant ESM1b scores, though a subset of variants with decreased expression was not successfully predicted (Figure 2A; $\rho = 0.586$). There was similar correlation between ESM1b scores and migration effect sizes but lower correlation with proliferation effect sizes (Figures 2B and 2C; $\rho = -0.593$ for migration and $\rho = -0.500$ for proliferation). When averaged by position, the DMS results correlated better with ESM1b for all three assays (Figures 2D–2F). Correlation between ESM1b and the DMS results varied by protein domain, with consistently poor correlation within the CTD (Figure 2G). Consistent with previous reports, we found that ESM1b pathogenicity scores did not reliably distinguish between wild-type (WT)-like and GoF variants (Figure 2H).

We investigated whether fine-tuning ESM1b with limited amounts of training data from our DMS screen would yield a better supervised predictor of DMS results. Across training sets from 2 to 11 variants per position, we observed a sharply increased correlation between predicted scores and the DMS effect sizes with just 2 variants per position, with further improvements with increasing variants per position (Figure 2I). For example, for the expression data, correlation was $\rho = 0.586$ with base ESM1b scores, but the mean ρ was 0.736 after training with 2 variants per position (~10% of the DMS data). Across all three phenotypes, 2 variants per position produced ~60% as much improvement as was achieved with 11 variants per position. The fine-tuning included a final ridge regression step, as we observed that this resulted in greater increases in correlation, particularly with smaller training sets (Figure S3A).

We then examined domain-specific changes, observing the most striking improvement within the N-terminal domain (NTD) and the smallest amount of improvement within the CTD, with intermediate improvements for other domains (Figure 2J). Domains with higher correlations with base ESM1b continued to be the domains with higher correlations after fine-tuning, with the exception of the NTD, for which base ESM1b was poorly predictive, but even modest amounts of training data resulted in a large improvement.

The improved correlation was visible when plotting fine-tuned ESM1b and DMS expression effect sizes relative to plotting with baseline ESM1b (compare Figure S3B with Figure 2A). There was marginally better separation between the WT-like and GoF variants with 2 variants per position training, with greater improvement with 11 variants per position (Figure 2K). These findings suggest that, although a screen with as few as two variants per position can enhance predictions of variant effects, identification of GoF variants will likely require more intensive experimental analysis or improved VEP algorithms.

To ensure that these observations were not unique to ESM1b, we also compared our DMS results with an alternative VEP tool, AlphaMissense (AM). AM was produced through fine-tuning AlphaFold on human and primate population variant frequencies and has been shown to have state-of-the-art zero-shot performance at predicting DMS results.[7,34] We observed high correlation between AM scores and the expression, migration, and proliferation effect sizes (Figures S4A–S4C). Correlation was again higher for the position-averaged data relative to individual variant data (Figures S4D–S4F). As with ESM1b, correlation between AM and the DMS data varied by protein domain, with much lower correlation with the CTD (Figure S4G). As with ESM1b, AM better

Figure 2. Improving computational VEP using limited experimental data

(A–C) Plots of missense variants comparing ESM1b scores and (A) expression Z score, (B) migration Z score, and (C) proliferation Z score, colored by domain.

(D–F) Plots of positions comparing mean ESM1b score and (D) mean expression Z score, (E) mean migration Z score, and (F) mean proliferation Z score, colored by domain.

(G) Plot of Spearman correlation between ESM1b score and variant expression, migration, and proliferation Z scores, partitioned by domain.

(H) Plot comparing the distribution of variant ESM1b scores, partitioned by DMS phenotype. Whiskers extend to maximum and minimum, and boxes show 25%, median, and 75%.

(I) Plot of Spearman correlation between fine-tuned ESM1b scores and DMS Z scores as training set size varies. Shows mean and SD.

(J) Plot comparing Spearman correlation when using ESM1b score or fine-tuned ESM1b scores with 2 variants/position or 11 variants/position training sets, partitioned by domain. Shown are means and SD.

(K) Plot comparing distribution of fine-tuned ESM1b scores for representative 2 variant per position training and representative 11 variant per position training, partitioned by DMS phenotype. Whiskers extend to maximum and minimum, and boxes shows 25%, median, and 75%.

(L) Plot comparing Spearman correlations between human GPR68 DMS results[23] and base ESM1b or ESM1b fine-tuned on P2RY8 expression results for 50 or 300 fine-tuning steps.

(M) Plots showing difference in Spearman correlation between base ESM1b and 50-step fine-tuned ESM1b on GPR68 surface expression (L) or pH 5.5 signaling (R) (a value >0 indicates greater correlation with fine-tuning).

In (G), (I), and (J), sign is inverted for migration and proliferation, so all three correlations have the same sign. GoF, Z score >2 for expression, < -2 for migration, proliferation; LoF, Z score < -2 for expression, >2 for migration, proliferation. See also Figures S3 and S4.

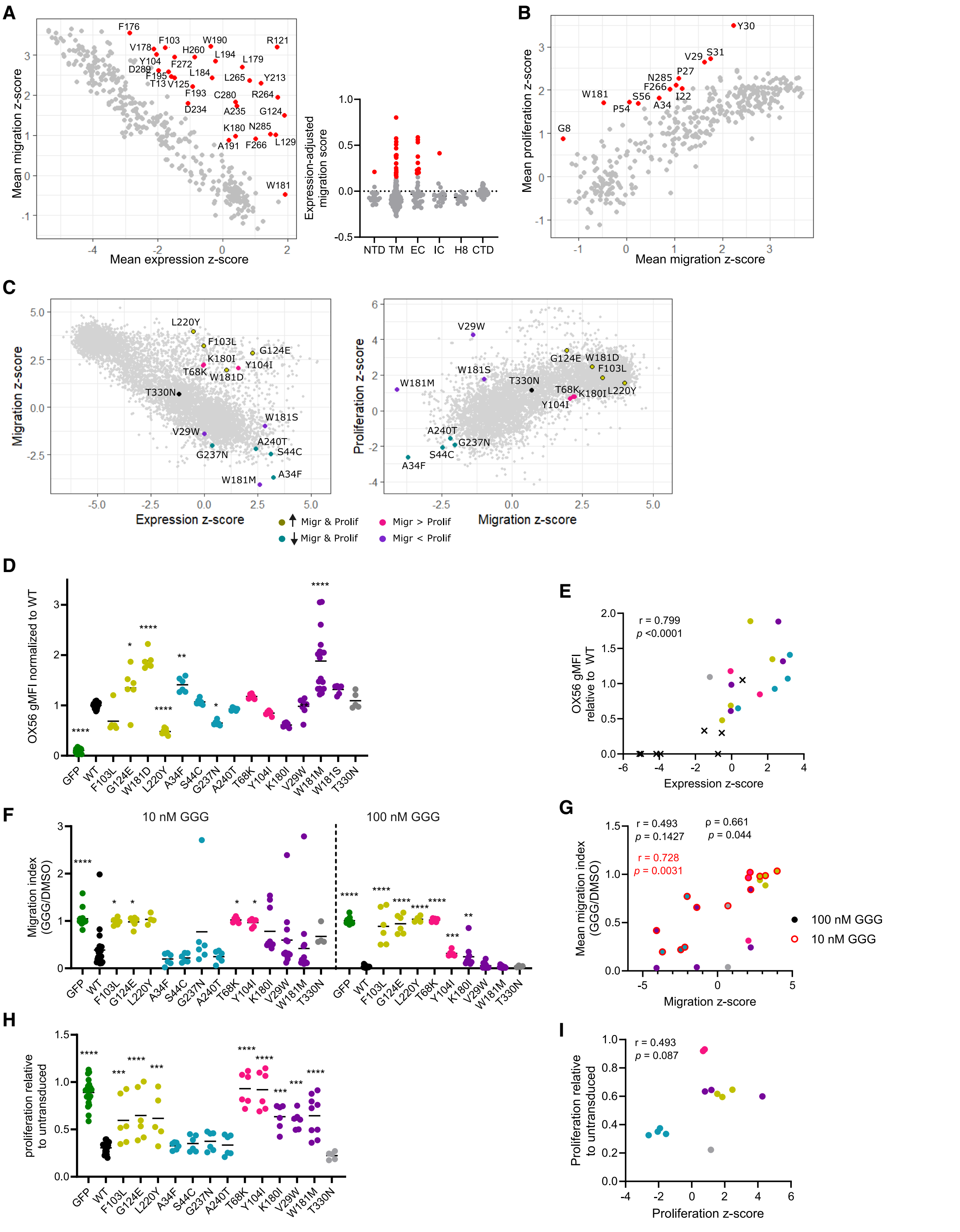

Figure 3. Validation of variants with independent effects on expression and function

(A) Left: plot of positions comparing expression and migration mean *Z* score; those with a high expression-adjusted migration scores are colored red. Right: expression-adjusted migration score for each position, partitioned by domain; lines are medians.

(B) Plot of positions comparing migration and proliferation mean *Z* scores, those with a high migration-adjusted proliferation score are colored red.

(legend continued on next page)

distinguished between LoF variants and WT-like variants than between GoF variants and WT-like variants (Figure S4H). LoF variants that had a benign AM pathogenicity score[7] were overrepresented in the EC and intracellular IC domains and CTD relative to correctly assigned LoF variants (Figure S4I). In addition, these misclassified variants were overrepresented at positions in which the WT amino acid is positively charged and among variants in which the substituted amino acid was glycine or an aliphatic hydrophobic residue (Figure S4I).

Fine-tuning comparable to what was performed with ESM1b was not possible with AM, given that the specific model weights are not publicly available. However, we implemented an augmented one-hot encoded (OHE) ridge regression model described previously,[35] using the same variant training sets as in the fine-tuning iterations. This resulted in improved correlation between predicted scores and the DMS effect sizes, with greater gains with increasing training set size (Figure S4J). The degree of improvement was less than with ESM1b fine-tuning (Figure S3C). OHE regression for ESM1b also produced less improvement than fine-tuning ESM1b, particularly with larger training sets (Figure S3D). In summary, two different approaches using a subset of our DMS data were able to improve VEP relative to two distinct state-of-the-art VEP tools alone.

Finally, we investigated whether the DMS results for P2RY8 could be used to improve VEP for another GPCR. Recently a DMS study was published for GPR68, a pH-sensing receptor that, like P2RY8, is a class A, subgroup δ GPCR.[23] The correlations between ESM1b scores and GPR68 DMS scores for missense variants were $\rho = 0.560$ for surface expression and $\rho = 0.529$ for pH 5.5 signaling, comparable to what was seen with P2RY8. We found that, after fine-tuning ESM1b on P2RY8 variant expression scores with 300 optimization steps, there was no improvement in VEP for GPR68 (Figure 2L). We restricted to 50 optimization steps to reduce overfitting and now observed a small increase in correlation of $\sim$0.02. We did observe varying effects by protein domain, with the greatest improvement in the TM domain for expression and function (Figure 2M). This finding corresponds to greater conservation of this region across GPCRs.[36,37]

In summary, use of a subset of our DMS data was able to strongly improve VEP relative to two distinct state-of-the-art VEP tools alone but showed limited ability to generalize to improvements in VEPs in a different GPCR.

Validation of variants with independent effects on expression and function

Although variant effects on cell migration and surface expression were overall inversely correlated, there were several hundred variants that affected migration independent of expression (Figure 1C). We identified 29 residues with substantially higher mean effect size across substitutions in migration than in expression; 27 of 29 of these positions were located within a TM helix or EC loop (Figure 3A), including some conserved residues known to be critical for GPCR signaling, such as R121^{3x50} of the D(E)-R-Y motif[17] (the superscript provided at the initial reference to position is generic GPCR residue numbering per the revised Ballesteros-Weinstein method for class A GPCRs.[36,38]). These 29 positions also generally had a high expression-adjusted proliferation score (Figure S5A). Performing a comparable analysis on migration and proliferation, there were 12 positions with a high migration-adjusted proliferation scores and none with the converse (Figure 3B).

We performed validation studies of several variants, drawing from different regions of the protein and representative of various functional phenotypes with preserved surface expression: increased migration and proliferation (F103^{3x32}L, G124^{3x53}E, W181^{ECL2}D, and L220^{5x65}Y); decreased migration and proliferation (A34^{1x43}F, S44^{1x53}C, G237^{6x35}N, and A240^{6x38}T); increased migration relative to proliferation (T68^{2x49}K, Y104^{3x33}I, and K180^{ECL2}I); increased proliferation relative to migration (V29^{1x38}W, W181^{ECL2}M, and W181^{ECL2}S); and WT-like function (T330^{CTD}N) (Figure 3C). Of these variants, seven involve positions with high expression-adjusted migration scores, and four involve positions with high migration-adjusted proliferation scores. All variants were confirmed to support surface expression, and high correlation was observed between screen and validation results for these 15 variants (Figures 3D and 3E; r = 0.506, $p = 0.0543$). The inclusion of 8 additional P2RY8 variants that we have characterized previously[25,28] resulted in a higher correlation (Figure 3E; r = 0.799, $p < 0.0001$). Despite P2RY8 expression, cells with these variants showed a range of migratory capacity toward CXCL12 in the presence of GGG (Figure 3F). We observed high correlation between the screen and validation results at both high and low GGG exposures (Figure 3G; 100 nM, r = 0.493, $p = 0.1427$, though Spearman $\rho = 0.661$, $p = 0.044$; 10 nM, r = 0.728, $p = 0.0031$). We also performed validation proliferation studies, comparing changes in

(C) Plots of missense variants comparing expression and migration *Z* scores (left) or migration and proliferation *Z* scores (right), with select variants labeled.

(D) P2RY8 surface expression normalized to WT P2RY8; lines are means. Shown are pooled results from 9 experiments, total 5–26 biological replicates per condition.

(E) Plot of DMS expression *Z* score and WT-normalized expression for select variants. Circles are those in D, with corresponding colors, and crosses are variants assayed previously.[25,28]

(F) Migration of cells toward CXCL12 in the presence of GGG relative to DMSO (vehicle); each dot is a biological replicate, and lines are means. Shown are pooled results from 9 experiments, total 4–23 biological replicates per condition.

(G) Plot of DMS migration *Z* score and migration indices for variants shown in (F), with corresponding colors.

(H) Proliferation over 13 days relative to untransduced cells; each point is a biological replicate, lines are means. Shown are pooled results from 8 experiments, total 5–21 biological replicates per condition.

(I) Plot of DMS proliferation *Z* score and proliferation results for variants as shown in (H), with corresponding colors.

(D, F, and H) One-way ANOVA test with Dunnett's multiple comparisons test, each variant compared to the WT; adjusted $^{*}p < 0.05$, $^{**}p < 0.01$, $^{***}p < 0.001$, $^{****}p < 0.0001$.

(E, G, and I) Two-tailed *p* values; r, Pearson correlation coefficient.

See also Figure S5.

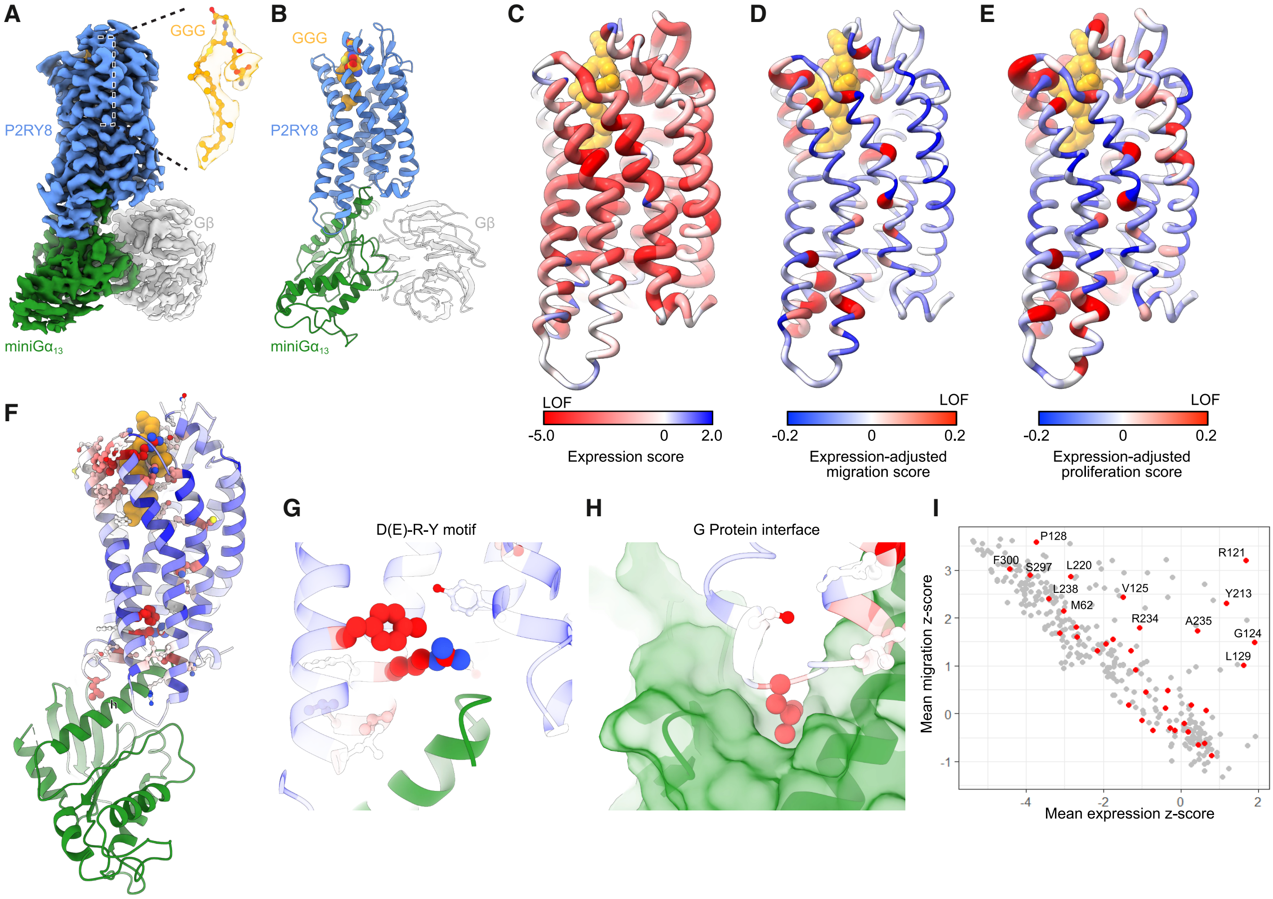

Figure 4. Structure of activated ligand-bound human P2RY8

(A and B) Cryo-EM density map (A) and ribbon model (B) of active human P2RY8 bound to GGG (orange). P2RY8 is fused to miniGα_{13} (green) and bound to G β_1 (gray).

(C–E) Ribbon model of P2RY8, colored to indicate DMS mean scores for (C) expression, (D) expression-adjusted migration, and (E) expression-adjusted proliferation. Gray, no data available.

(F–H) Expression-adjusted migration scores for individual residues with close-up views of (G) the D(E)-R-Y motif and (H) the G protein interface.

(I) Plot comparing mean expression Z scores and mean migration Z scores by position; Gα_{13}-interacting positions are colored red.

See also Figures S6–S8.

relative abundance of transduced and untransduced cells. Over 13 days, WT P2RY8 resulted in an ~2/3 reduction in frequency compared to untransduced cells. Although the effects of the variants on proliferation appeared to be largely in agreement with those seen in the screen, the trend was not statistically significant (Figures 3H and 3I). In summary, individual variant validation across all three assays yielded findings that were in close accord with the screen.

Structure of activated ligand-bound human P2RY8

To facilitate understanding the structural basis of the variant phenotypes, we used cryo-EM to determine the structure of activated, GGG-bound human P2RY8. We generated a C-terminal fusion of P2RY8 with a minimized and stabilized version of Gα_{13} (miniGα_{13}), as described previously for other heterotrimeric G proteins[39,40] (Figure S6A). P2RY8-miniGα_{13} was purified in the presence of GGG and further complexed with recombinant Gβ1γ2 (Figure S6B). The resulting preparation was analyzed by single-particle cryo-EM, which yielded a 2.7 Å map of the complex (Figure S7; Table S3). While the receptor and miniGα_{13} were well resolved, only a portion of the Gβ_1 was visible in the resulting density; the majority of Gγ_2 was not resolved. Importantly, the map revealed a density consistent with GGG located in the canonical class A GPCR orthosteric ligand-binding pocket (Figures 4A and 4B).

We mapped DMS results onto the receptor structure, illustrating the structural elements most essential for expression (Figure 4C). Positions essential for P2RY8 expression were primarily concentrated in the TM regions, with EC and IC loops more tolerant to variants. We also mapped expression-adjusted migration and expression-adjusted proliferation scores (Figures 4D and 4E). Positions with high expression-adjusted migration and proliferation scores were clustered in the same few regions within the protein. This analysis revealed two critically important regions: residues surrounding the GGG ligand and key interacting residues with Gα_{13} (Figure 4F).

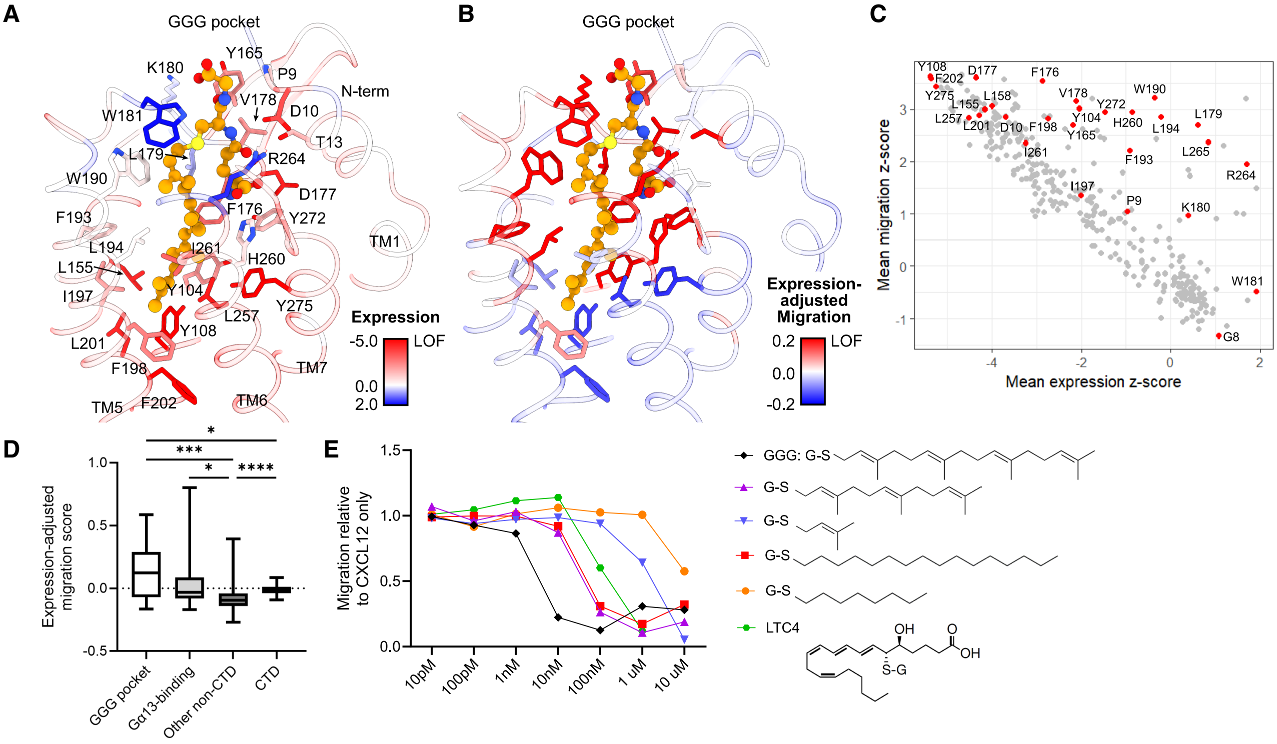

Figure 5. Structural features of GGG recognition

(A and B) Close-up view of the GGG binding site in P2RY8.

(A) Expression mean Z scores mapped onto P2RY8 residues (sticks/ribbon) that form the GGG binding site (orange sticks/spheres).

(B) Migration mean Z scores mapped onto P2RY8 residues (sticks/ribbon) that form the GGG binding site (orange sticks/spheres).

(C) Plot comparing expression and migration mean Z scores by position; GGG-interacting positions are colored red.

(D) Distribution of expression-adjusted migration scores for positions of specified categories. Whiskers extend to maximum and minimum, and boxes shows 25%, median, and 75%. Kruskal-Wallis test with Dunn's multiple comparisons test; ns, nonsignificant. ***$p < 0.001$, ****$p < 0.0001$.

(E) Migration of P2RY8-transduced WEHI-231 cells in the presence of 50 ng mL^{-1} CXCL12 with or without GGG or candidate P2RY8 ligands of varying concentrations, normalized to migration to CXCL12 alone. Representative results are from one of two independent experiments. G-S, glutathione; LTC_4, leukotriene C_4.

See also Figure S8.

The P2RY8 interaction with miniGα_{13} is highly similar to the canonical conformation observed for most GPCR-G protein complexes to date.[37,41] Our DMS provides an unbiased view of key regions of P2RY8 that are important for signal transduction. We identified residues directly contacting miniGα_{13} with high expression-adjusted migration scores (Figures 3A and 4F). Although we lack an inactive-state structure of P2RY8, the importance of these residues is underscored by their conservation among class A GPCRs and key contacts they make to stabilize P2RY8 or interact with Gα_{13}. For example, both R121 and Y213 directly bind to the α5 helix of miniGα_{13} (Figure 4G). As has been observed previously for many class A GPCRs, Y213 engages Y293^{7x53} in a bonding network that likely stabilizes active P2RY8. Additionally, L129 binds to a conserved hydrophobic cavity in Gα_{13} (Figure 4H). The 37 P2RY8 positions in contact with miniGα_{13} showed a range of mutation tolerance ranging from highly sensitive for both expression and migration to largely tolerant (Figure 4I). Individual missense variants for two of these positions, G124E and L220Y, were included in the variant validation set already described; both of these variants were expressed but strongly deleterious to function (Figures 3D, 3F, and 3H). Taken together, the DMS results and cryo-EM structure provide complementary, concordant insights into receptor function.

Structural features of GGG recognition

GGG binds to P2RY8 in a large solvent-facing cavity, with a combination of hydrophilic interactions with the glutathione head group and hydrophobic interactions along the geranylgeranyl tail (Figures 5A and S8). The glutathione head group is located toward the EC side of the pocket, while the geranylgeranyl tail extends deeply within the core of the receptor. We found that 13 of 28 residues that contact GGG have high expression-adjusted migration scores, with residues with lower expression-adjusted migration scores predominantly in the deeper portion of the GGG-binding pocket (Figures 5B and 5C). High-scoring positions contacting the GGG head group include K180, H260^{6x58}, R264^{ECL3}, and Y272^{7x32}. Residues H260, R264, and Y272 engage the glutamate residue in GGG, with H260 making a direct hydrogen bond with the carboxylic acid. Additionally, several positions contact the proximal geranylgeranyl tail closest to the thioether cysteine in GGG,

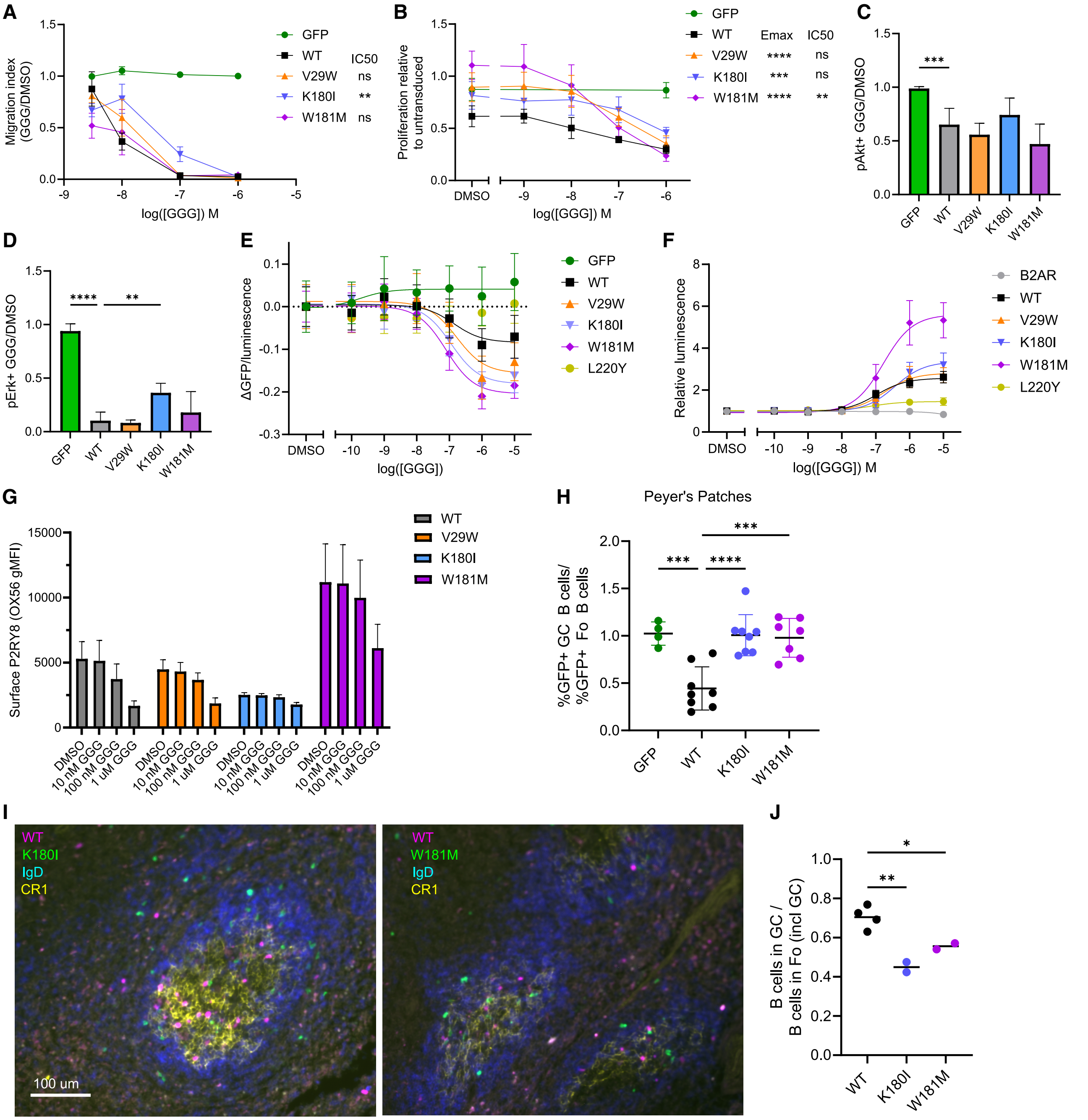

Figure 6. *In vitro* and *in vivo* delineation of heterogeneous variants effects

(A) Migration of transduced Ly8 cells toward CXCL12 with varying concentrations of GGG relative to DMSO (vehicle) and normalized to untransduced cells; shown are means and SEMs. Pooled results are from 10 experiments, total 2–22 (mean 9.5, SD 4.9) replicates per condition.

(B) Effect of GGG on proliferation over 7 days relative to untransduced cells; shown are means and SDs. Pooled results are from 5 experiments, total 6–12 replicates per condition.

(C and D) Transduced Ly8 cells exposed to GGG or vehicle were assayed using phospho-flow cytometry; graphs show the ratio of pAkt^{+} (C) or pErk^{+} (D) cells after GGG vs. vehicle, normalized to GFP vector. Shown are means and SDs. Pooled results are from 4 experiments, total 6 replicates per condition.

(E) BRET trimeric G protein activation assay: ratio of luminescence and GFP fluorescence normalized to DMSO (vehicle) condition. Shown are means and SDs. Pooled results are from 4 experiments, total 9–12 replicates per condition.

(F) NanoBiT β-arrestin recruitment assay: luminescence relative to DMSO (vehicle) is plotted. Shown are means and SDs. Pooled results are from 4 experiments, total 12 replicates per condition.

(G) P2RY8 surface expression of transduced Ly8 cells after 45 min GGG treatment. Pooled from 3 experiments, total 6–8 replicates per condition.

(legend continued on next page)

including L179[ECL2], W190[5x35], L194[5x39], and L265[ECL3]. In addition, two residues in contact with the GGG head group, G8[NTD] and W181, have high migration-adjusted proliferation scores (Figure 3B). The validation assays described above included variants in three residues in the GGG-binding pocket (Y104I, K180I, and W181M), all of which showed decreased proliferation restraint and two of which showed decreased migration inhibition (Figures 3F and 3H). Examination of the DMS results for all binding pocket residues showed significant intolerance to mutation for most positions (Figure 5C). In summary, GGG-binding pocket positions and the $G\alpha_{13}$-contacting positions showed increased average expression-adjusted migration scores (Figure 5D). That is, positions that were either part of the GGG-binding pocket or in contact with $G\alpha_{13}$ made up 20 of 29 non-CTD positions with high expression-adjusted migration scores and 45 of 282 of the remaining non-CTD positions ($p < 0.0001$, Fisher's exact test).

Our DMS results suggest that the glutathione head group combined with the proximal prenyl group in the geranylgeranyl tail are required for P2RY8 activation. We tested our DMS-driven model for the most important chemical components of GGG required for P2RY8 activation with analogs. We have shown previously that glutathione alone and geranylgeranyl-pyrophosphate are both inactive at P2RY8.[28] As we have demonstrated previously, the cysteinyl leukotriene C_4 (LTC_4) is active at P2RY8 with ~100-fold less potency than GGG in a migration inhibition assay[28] (Figure 5E). Although the specific orientation of LTC_4 within the P2RY8 binding site is uncertain, it is likely that the glutathione head group and the aliphatic tail of LTC_4 occupy similar general positions as GGG. The ability of LTC_4 to activate P2RY8 suggested that the specific shape of the geranylgeranyl tail of GGG is not required for P2RY8 activation. Indeed, a saturated 16-carbon chain and a 15-carbon farnesyl group, which is one prenyl group smaller than geranylgeranyl, both activated P2RY8 with ~10-fold less potency than GGG (Figure 5E). Further diminutions in tail size significantly reduced potency, as seen for glutathione conjugated to a single prenyl group (~1,000-fold less potent than GGG) and glutathione conjugated to a saturated 8-carbon chain (~5,000-fold less potent than GGG). These results illustrated that ligand potency varied with the degree of contact possible between the hydrophobic tail and P2RY8 within the binding site.

Significant functional phenotypes with subtle proximal signaling changes

Where the P2RY8 signaling pathways affecting migration and proliferation diverge has not been established, but our screen results suggested that biased agonism of these activities was possible; on initial validation, V29W and W181M showed WT-like behavior in migration but decreased proliferation restraint, whereas K180I showed decreased but not absent function in both phenotypes (Figures 3F and 3H). K180I and W181M are part of the GGG-binding pocket (Figure 5A), whereas V29W is in TM1. We performed more in-depth validation of these variants to explore the mechanism for these phenotypes.

We first examined migration inhibition across a range of GGG concentrations and found that W181M and V29W were not significantly different from the WT, whereas K180I inhibited migration less than the WT, with a significantly higher IC_{50} (Figure 6A). A different pattern was observed when examining proliferation with a range of exogenous GGG concentrations. (We had not added exogenous GGG to the culture in the proliferation arm of the screen or initial validation because we had observed previously that B cells and B cell lines secrete GGG in amounts sufficient to affect proliferation.[27,28,32]) All three variants showed responses that were significantly different from the WT. W181M was most different from the WT at lower GGG concentrations but equivalent at high concentrations, V29W had a similar but less striking pattern, and K180I had a moderate deficit at all GGG concentrations relative to the WT (Figure 6B). In sum, at low levels, W181M showed greater defects in proliferation restraint and K180I greater defects in migration inhibition (Figures 6A and 6B). This provided evidence that the P2RY8 signaling that drives these two processes is different at the receptor itself.

We then assayed elements of the known P2RY8 signaling pathways. P2RY8 activation results in decreased phosphorylation of Akt and Erk, and deleterious P2RY8 variants can disrupt this effect.[28,32] We used phospho-flow to assess the phosphorylation status of these two kinases. As expected, WT P2RY8 allowed GGG to trigger a significant reduction in the frequency of $pAkt^+$ cells. Similar reductions were seen for V29W, K180I, and W181M (Figure 6C). For all variants, GGG also drove a decrease in $pErk^+$ cells but less effectively with K180I than the WT (Figure 6D). Overall, the phospho-flow results showed less separation between WT P2RY8 and the variants than what was seen in the migration and proliferation assays. This was further reflected in assays of RhoA activation, a known mediator of P2RY8 signaling.[32] The lower average RhoA activation in K180I and W181M relative to the WT was not statistically significant, though there was decreased RhoA activity with L220Y, a variant that was strongly deleterious in initial validation studies (Figure S9A).

(H) Ratio of GFP+ cell frequency among GC and follicular B cells in Peyer's patches of irradiated CD45.1 mice reconstituted with bone marrow transduced with EV-GFP, WT-P2RY8-GFP, K180I-GFP, or W181M-GFP. Each point is a mouse. Mean and SD are shown. Pooled from 3 experiments, total 4–8 mice per condition.

(I) Representative immunofluorescence micrographs. Polyclonal B cells transduced with K180I-GFP or W181M-GFP were co-transferred with WT-TagBFP into SRBC-immunized recipient mice. Green, anti-GFP; magenta, anti-TagBFP; yellow, anti-CR1 (GC label); and blue, immunoglobulin D (IgD; follicular B cell label). Scale bar, 100 μm, both images are of the same scale.

(J) Ratio of transduced cells within the GC to those within the follicle (including GC). A given mouse is represented by one WT dot and one K180I or W181M dot. Two independent experiments, one recipient mouse per variant per experiment, at least 45 GCs analyzed per mouse.

For (A) and (B), two-sided p value from z test comparison with WT with Benjamini-Hochberg multiple comparisons adjustment; E_{max} and IC_{50} were determined by non-linear regression (R). For (E) and (F) Curves were fit using log(agonist) vs. response, 3-parameter mode (GraphPad Prism). For (C), (D), (H), and (J), one-way ANOVA test with Dunnett's multiple comparisons test, each variant compared to WT; adjusted p value: ns, $p > 0.05$, **$p < 0.01$, ***$p < 0.001$, ****$p < 0.0001$. See also Figure S9.

We then used two luciferase-based reporter assays to assess variant effects on signaling pathway elements more proximal to the receptor. To examine trimeric G protein activation, we used TRUPATH, a bioluminescence resonance energy transfer (BRET) approach.[42,43] Receptor activation results in dissociation of $G\alpha_{13}$-luciferase from $G\gamma$-GFP and therefore a decreased GFP signal relative to the luciferase signal. The strongly deleterious variant L220Y was unable to drive G protein activation (Figure 6E). In contrast, WT P2RY8, V29W, K180I, and W181M were all able to do so in a dose-responsive manner with comparable IC_{50} values (Figure 6E). Although the variants showed a larger absolute change than the WT, the degree to which this reflected differences in expression versus intrinsic per-receptor maximum activity was not determined. We also examined activity in the absence of exogenous GGG and found that V29W and W181M were not significantly different from the WT, whereas K180I and L220Y showed less $G\alpha_{13}$ activation (Figure S9B). This suggests that the defect in proliferation restraint by W181M is not secondary to reductions in basal receptor activation of G_{13}. To measure β-arrestin recruitment, we used the NanoBiT system.[44] In this system, when the GPCR and β-arrestin are in close proximity, two luciferase fragments interact to form a functional luciferase. In contrast to β2AR (a GPCR that does not bind GGG), robust recruitment was seen by WT P2RY8, V29W, K180I, and W181M, with W181M having higher maximum recruitment, though not a significantly different EC_{50} (Figure 6F). In sum, neither of these assays provided a mechanistic explanation for the effects of these variants on migration and proliferation.

For many GPCRs, β-arrestin recruitment promotes receptor internalization.[18] We observed this effect with WT P2RY8 (Figure 6G). V29W showed a response similar to the WT, whereas W181M had a higher baseline expression and less of a relative decrease at 100 nM GGG, and K180I had a lower baseline expression but only a modest reduction in surface expression even at high GGG concentrations (Figure 6G). Taken together, our assays suggest that the ability to recruit β-arrestin is not sufficient to produce a normal P2RY8 internalization response to ligand. Although dose-response studies further validated the migration and proliferation phenotypes, assays of proximal signaling elements did not delineate the specific mechanism underlying these phenotypes.

Disparate migration and proliferation phenotypes of two P2RY8 variants *in vivo*

Given their contrasting phenotypes *in vitro* as described above (Figures 6A and 6B), we decided to further assay K180I and W181M *in vivo*. P2RY8, although conserved across vertebrates, has been lost in rodents. However, previous work has illustrated that human P2RY8 introduced into mice is able to affect GC B cell abundance in Peyer's patches and B cell positioning in lymphoid follicles.[27,28] To examine variant effect on GC B cell abundance, we used transduced bone marrow to reconstitute irradiated congenic mice and, after reconstitution, compared the proportion of transduced cells among GC and follicular B cells. We have shown previously that WT P2RY8-transduced cells are less prevalent among GC B cells than follicular B cells in Peyer's patches, whereas there is not a significant difference in mesenteric lymph nodes or the spleen.[27] We reproduced this effect for the WT. In contrast, neither K180I nor W181M showed altered prevalence in GC compared to follicular B cells in any of the three tissues (Figures 6H, S9C, and S9D). This argues that the phenotype produced by WT P2RY8 requires a high degree of receptor activity and that even moderate defects in proliferation restraint, as seen *in vitro* with K180I (Figure 6B), prevent the *in vivo* growth regulation phenotype. The still greater deficit of W181M at physiological ranges of GGG *in vitro* (Figure 6B) therefore also unsurprisingly translates to failure to recapitulate the WT P2RY8 phenotype.

In a second *in vivo* assay, we examined the effect of these mutants on B cell positioning in lymphoid tissues. To do so, activated polyclonal murine B cells, transduced with variant or WT P2RY8, were co-transferred into pre-immunized mice before harvest for immunofluorescent microscopy analysis. We have shown previously that, in the absence of P2RY8, transferred activated B cells do not localize into the GC, whereas most cells with WT P2RY8 congregate within the GC.[27] The WT P2RY8-transduced B cells in our experiment reproduced this pattern, with approximately 80% of the cells within the follicle being located in the GC (Figures 6I and 6J). For both K180I and W181M, approximately half of the transduced cells within the follicle were in the GC (Figures 6I and 6J). This *in vivo* phenotype is consistent with the modest migration inhibition defect seen in *in vitro* for K180I. The mechanism for the decreased localization of W181M *in vivo* compared to the WT, despite at least WT-like responsiveness *in vitro*, is less evident. Although previous direct GGG measurement in lymphoid tissue homogenates found a concentration of approximately 10 nM,[28] the interstitial concentrations at different sites within the lymphoid tissue are uncertain aside from a relatively lower level within the GC.[27,28] The *in vivo* behavior differences for W181M compared to WT that contrast with the *in vitro* migration assay (Figure 6A) may be a consequence of other differences resulting from this variant, including higher baseline surface expression and decreased internalization in response to ligand, differences in response kinetics, and more complicated chemokine and GGG gradients *in vivo* compared to the Transwell assay. Overall, the results of these *in vivo* assays provide the first evidence that it is possible for P2RY8-driven confinement within the GC to occur without causing decreased prevalence within the GC B cell pool.

Phenotypes of germline and lymphoma-associated P2RY8 variants

We analyzed hundreds of human germline missense variants in P2RY8 from gnomAD[2] and noted highly varied phenotypes per our DMS results (Figure 7A). Comparison of allele frequency and DMS effect sizes revealed statistically significant correlations, consistent with selective pressure. This was apparent both when the 476 individual variant frequencies were considered separately and when consolidating these variants within the 260 involved positions (Figures 7B and S10A–S10E). Outside of the common variant V125I, these variants rarely occur in homozygosity (∼0.2%), though compound heterozygosity cannot be determined. However, we have shown previously that haploinsufficiency can occur from deleterious variants of P2RY8.[32]

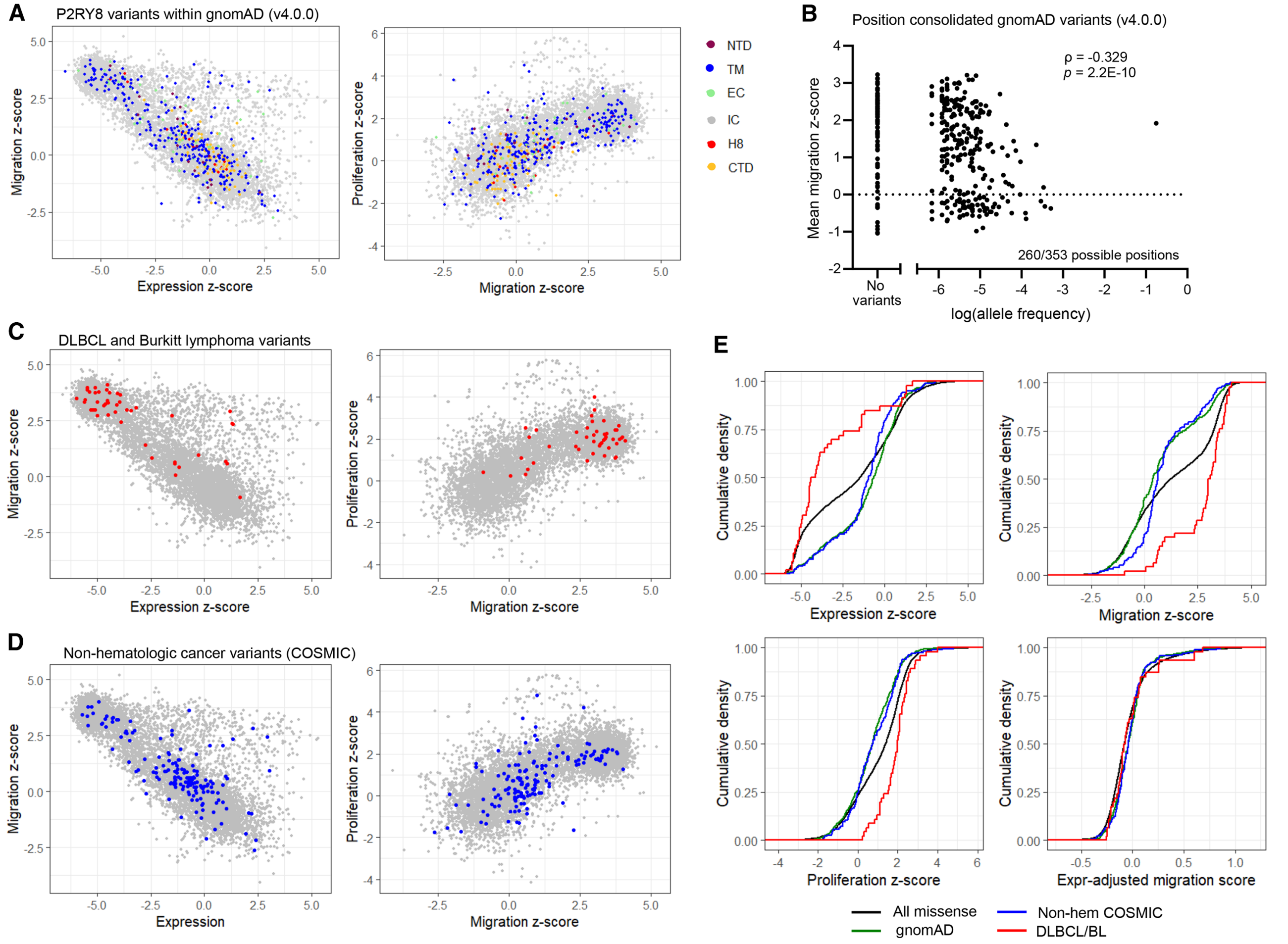

Figure 7. Phenotypes of germline and lymphoma-associated P2RY8 variants

(A) Plots of missense variants, comparing expression and migration (left) and migration and proliferation (right) of variants, with gnomAD variants colored by domain.

(B) Plot comparing allele frequency for missense variants in gnomAD by position and mean migration *Z* score for each position. *p* value was calculated using algorithm AS 89 with Edgeworth series approximation.

(C and D) Plots of missense variants, comparing expression and migration (left) and migration and proliferation *Z* scores (right), with (C) 48 variants reported in DLBCL or Burkitt lymphoma colored red and (D) non-hematologic cancer variants colored blue.

(E) Cumulative density plots of expression, migration, and proliferation *Z* scores and expression-adjusted migration scores for all missense variants (black), gnomAD (green), DLBCL/Burkitt lymphoma (red), and non-hematologic cancer (blue).

See also Figure S10.

The variants reported in gnomAD include four of the variants validated above (Figures 3D–3F, and 3H; F103L, which shows LoF, and G237N, A240T, and T330N, which are WT like).

P2RY8 missense variants are common in two GC B cell-derived lymphomas, DLBCL and Burkitt lymphoma. We plotted the effect sizes of 48 variants compiled from two sources (Catalogue of Somatic Mutations in Cancer [COSMIC][31,45] and Muppidi et al.[25]) and observed that nearly all of these variants reduced P2RY8 expression and decreased its migration inhibition, and none of them showed increased expression or function (Figure 7C). The 15 of these variants also observed in gnomAD as germline variants all have an allele frequency <0.0001, indicating that it is very likely that they were somatically acquired in these cancers. Of the 41 positions with lymphoma variants, six had high expression-adjusted migration scores (Figure 3A; F103, Y104, $A191^{5x36}$, A235, R264, and $F266^{ECL3}$). In addition, another involved position, $Y30^{1x39}$, has the highest mean proliferation effect size of any position (Figure 3C). The lymphoma variants include residues that interact with $G\alpha_{13}$, including $M62^{2x43}$, A235, and $S297^{8x47}$, and residues in the GGG-binding pocket, including Y104, $F176^{ECL2}$, $D177^{ECL2}$, and R264. In addition, our screen revealed that six of the lymphoma variants that are LoF would not have been classified as pathogenic based on AM pathogenicity score ($P20^{1x29}$L, $R86^{ECL1}$C, $A140^{4x42}$T, R264H, $S270^{ECL3}$N, and $K276^{7x36}$R; Tables S1 and S2).

Turning to 159 missense variants that have been identified in non-hematologic cancers (COSMIC), we observed a broadly distributed set of phenotypes akin to that seen in germline

variants (Figure 7D). Although 67 of these variants are present in gnomAD, only 3 have an observed allele frequency >0.0001 (V125I, R133^{34x55}P, and V333^{CTD}L), again suggesting predominantly somatic mutagenesis. Ten of these variants are also present in the DLBCL/Burkitt lymphoma set. Comparison of the phenotype distributions for these four sets of variants using Kolmogorov-Smirnov tests revealed statistically significant differences, with gnomAD and non-hematologic cancer variants containing fewer deleterious variants than the set of all missense variants and DLBCL/Burkitt lymphoma variants being enriched for deleterious variants (Figures 7E and S10F). The relative lack of such enrichment or depletion when looking at expression-adjusted migration scores argues that this is largely driven by variants that affect both expression and function. These findings aligned with previous observations supporting P2RY8 as a cancer driver gene in DLBCL and Burkitt lymphoma, in contrast to non-hematologic cancers in which P2RY8 variants are more likely passenger mutations.

DISCUSSION

Here, we report near-saturation DMS of human P2RY8, a G_{13}-coupled GPCR important for GC B cell confinement, defining the expression, migration, and proliferation phenotypes for these variants. Use of these different phenotypes allowed delineation of variants that affected both expression and function, affected function independently of expression, and discrepantly affected migration and proliferation.

VEP algorithms have improved significantly and, particularly at the position level, are highly accurate at identifying changes that will result in loss of protein function. In P2RY8, however, these methods were much less able to predict increases in protein expression or activity, echoing similar limitations in VEP analysis of other proteins.[7,9] Our work demonstrates that it is possible to generate new, more highly correlated prediction scores through the combination of VEP scores and limited amounts of experimental data. Although greater gains were achievable with actual fine-tuning of the protein language model itself, even an OHE regression-based approach, which does not require knowledge of the model weights, achieved significant improvements. Despite these sizable improvements in predictions for P2RY8 itself, use of P2RY8 experimental data could only modestly improve VEPs for GPR68, a recently profiled GPCR. We hypothesize that one challenge is selecting the appropriate number of fine-tuning cycles to improve predictions of other proteins without overfitting on the training data; the degree to which the optimum number of cycles will vary depending on the target protein also remains an open question. Another area for improvement may be the specific method of fine-tuning, of which there are multiple proposed approaches, some of which may result in greater generalizability than others.[46,47] Another hypothesis is that fine-tuning using a small set of proteins (rather than a single protein) will yield significantly better generalizability. A recent pre-print article offers partial support for this hypothesis; the authors fine-tuned ESM1v on DMS results from 25 proteins and then applied this to 24 proteins in ProteinGym, observing an average Spearman correlation increase of 0.025.[47] (Above, we noted an increase of 0.021 for GPR68 expression and 0.015 for pH 5.5 signaling.) A final hypothesis to consider is that incorporation of knowledge beyond amino acid sequences (e.g., functional annotations) may enhance the performance of protein language models.[48–50] Based on our results, we suggest that predictions across a family of GPCRs may be achieved more efficiently by profiling relatively few variants across many receptors rather than comprehensively profiling variants within a small number of receptors. Beyond single missense variants, future studies could assay the combinatorial effects of multiple variants, insertions or deletions, and other structural variants to enhance our understanding and ability to computationally predict epistasis in GPCRs.

The activated P2RY8 structure provides further understanding of the functional data obtained through the DMS. By identifying key residues involved in interactions with $G\alpha_{13}$ and GGG, as well as our work evaluating the potency of several alternative ligands, we lay the groundwork for additional studies aimed at developing alternative agonist and antagonist ligands of this receptor.

Overall, our work highlights the ability of phenotypic screens to detect subtle phenotypes that may otherwise be missed in classical GPCR signaling assays that report changes more proximal to the receptor. We believe that results from our screens provide a more complete picture of how P2RY8 activation manifests as a phenotype by integrating the compounding and amplified effects that result in subtle shifts in signal propagation through the cell. Future study of the basis of these variants' phenotypes will be useful in further defining P2RY8 function. Additionally, P2RY8 variants identified in this study will enable *in vivo* studies to probe the role of P2RY8 function in GCs,[28] B cell negative selection,[32] and T cell responses.[27]

Our work provides a resource for interpretation of the pathogenicity of nearly any given missense variant of P2RY8, whether germline or somatic, which may be of particularly utility in the setting of autoimmunity, where deleterious P2RY8 variants have already been identified in a small number of patients.[32] It also allows interpretation of any somatic P2RY8 variants observed in cancer, including identification of deleterious variants that may not be identified by current computational VEP tools.

Limitations of the study

We note several caveats of our work. The range of observed effect sizes is greater for expression than for migration, and both are greater than for proliferation. This may reflect details of the screen itself, in which the maximum possible enrichment differs between the readouts. Adjustments, such as running the proliferation assay for a longer period of time, might result in increased effect sizes, thereby enhancing phenotyping of proliferation. Our follow-up signaling assays did not enforce equal expression between different variants, making determination of their expression-independent effect more complicated. The cryo-EM structure presents the stable, active conformation of P2RY8. Therefore, the specific conformational changes that occur in the transition from the inactive to the active state have not been determined. In profiling the effects of select variants on $G\alpha_{13}$ activation, we acknowledge that our work has not ruled out possible changes in interactions with other G protein

isoforms, such as G$\alpha_{12,}$ though we note that previous work has shown that Gα_{13} is essential for the action of P2RY8 *in vivo* in mice.[25,26] Our exploratory studies of select variants did not clarify the mechanism by which a given variant may produce different degrees of defects in migration or proliferation restraint, particularly as the assays of trimeric G protein activity and β-arrestin recruitment did not show differences that might explain these phenotypes.

In conclusion, we used DMS and cryo-EM to comprehensively map the mutational landscape of the immunomodulatory GPCR P2RY8, advancing understanding of its function.

RESOURCE AVAILABILITY

Lead contact

Requests for further information and resources should be directed to and will be fulfilled by the lead contact, Taylor LaFlam (taylor.laflam@ucsf.edu).

Materials availability

Stable reagents generated within this study will be shared upon request to the corresponding authors.

Data and code availability

Sequencing data from the deep mutational scan were deposited in the NCBI Sequence Read Archive under BioProject PRJNA1179413 and are publicly available. Coordinates for the P2RY8-Gα_{13} complex were deposited in the RCSB Protein Databank under accession code 9ECJ. The EM density map for the P2RY8-Gα_{13} complex was deposited in the Electron Microscopy Databank under accession codes EMD-47912 (full map) and EMD-47914 (7TM map). Code used in the DMS analysis was deposited at https://github.com/yelabucsf/P2RY8_DMS and Zenodo at https://doi.org/10.5281/zenodo.15811041.

ACKNOWLEDGMENTS

T.N.L. was supported by the Pediatric Scientist Development Program (NICHD K12-HD000850) and the UCSF Center for Rheumatic Diseases. J.G.C. is an investigator of the Howard Hughes Medical Institute (HHMI), with work supported in part by the NIH grant R01 AI045073. C.J.Y. was supported by the NIH grants R01AI171184 and P01AI172523 and the Arc Research Institute and is a member of the Gladstone-UCSF Institute of Genomic Immunology and the Parker Institute for Cancer Immunotherapy. UCSF PFCC (RRID: SCR_018206) is supported in part by the grant NIH P30 DK063720 and by the NIH S10 instrumentation grant S10 1S10OD021822-01. Sequencing was performed at UCSF CAT, supported by the UCSF PBBR, the RRP IMIA, and the NIH 1S10OD028511-01. The Wynton HPC Co-Op cluster is supported by the UCSF research faculty and UCSF institutional funds. The authors thank the UCSF Wynton team for technical support of Wynton and Drs. Li Wang, Glen Gilbert, and David Bulkley at the UCSF Bay Area Cryo-EM Consortium for help with Glacios microscope operation. The cryo-EM equipment at UCSF is partially supported by the NIH grants S10OD020054, S10OD021741, and S10OD026881 and the HHMI. The authors thank Drs. Shinxin Yang and Rui Yan at the HHMI Janelia CryoEM Facility for help with Krios microscope operation and data collection. Molecular graphics and analyses were performed with UCSF ChimeraX, developed by UCSF RBVI, with support from NIH R01-GM129325 and OCICB, NIAID.

AUTHOR CONTRIBUTIONS

T.N.L., C.B.B., A.M., J.G.C., and C.J.Y. conceived and designed this study. T.N.L. performed DMS and follow-up experiments and some VEP analyses and led manuscript writing. C.B.B. performed cryo-EM sample preparation and data analysis. J.G.C., C.J.Y., A.M., and C.B.B. co-wrote the paper. T.D. and V.N. performed ESM1b fine-tuning. F.D.W. synthesized candidate ligands. E.L. performed migration assays with candidate ligands. J.A. participated in creating mouse chimeras. Y.X. participated in cloning and plasmid preparation. A.S. participated in signaling assays, cloning, and plasmid preparation. T.M. and N.B. assisted with VEP analysis.

DECLARATION OF INTERESTS

A.M. is a founder of Epiodyne and Stipple Bio, consults for Abalone, and serves on the scientific advisory board of Septerna and Alkermes. J.G.C. is a scientific advisory board member of Be Biopharma and consults for Lycia Therapeutics and DrenBio Inc. C.J.Y. is a founder of and holds equity in DropPrint Genomics (now ImmunAI) and Survey Genomics, a scientific advisory board member of and hold equity in Related Sciences and ImmunAI, a consultant for and hold equity in Maze Therapeutics, and a consultant for TReX Bio, HiBio, ImYoo, and Santa Ana. Additionally, C.J.Y is also an Innovation Investigator for the Arc Institute. C.J.Y. has received research support from the Chan Zuckerberg Initiative, Chan Zuckerberg Biohub, Genentech, BioLegend, ScaleBio, and Illumina.

STAR★METHODS

Detailed methods are provided in the online version of this paper and include the following:

- KEY RESOURCES TABLE
- EXPERIMENTAL MODEL AND SUBJECT DETAILS
  - Animals
  - Cell lines
- METHOD DETAILS
  - Variant pool generation
  - Variant pool screening
  - Sequencing library preparation
  - Sequencing analysis
  - ESM1b fine-tuning using P2RY8 DMS data
  - Individual variant validation
  - Proliferation assay with exogenous GGG
  - Candidate ligand migration assay
  - Phospho-flow
  - RhoA activation assay
  - TRUPATH BRET trimeric G protein assay
  - *NanoBit β-arrestin* assay
  - Bone marrow chimera generation and analysis
  - B cell transduction, transfer, and analysis
  - Expression and purification of P2RY8-miniGα13 protein
  - Expression and purification of Gβ1γ2
  - Preparation of the active-state of P2RY8-G13 complex
  - Cryo-EM vitrification, data collection, and processing
  - Model building and refinement
  - Chemical synthesis
  - Previously available data
- QUANTIFICATION AND STATISTICAL ANALYSIS

SUPPLEMENTAL INFORMATION

Supplemental information can be found online at https://doi.org/10.1016/j.xgen.2025.100981.

Received: February 18, 2025
Revised: June 16, 2025
Accepted: July 30, 2025
Published: September 9, 2025

REFERENCES

1. Bycroft, C., Freeman, C., Petkova, D., Band, G., Elliott, L.T., Sharp, K., Motyer, A., Vukcevic, D., Delaneau, O., O'Connell, J., et al. (2018). The UK Biobank resource with deep phenotyping and genomic data. Nature *562*, 203–209. https://doi.org/10.1038/s41586-018-0579-z.

2. Karczewski, K.J., Francioli, L.C., Tiao, G., Cummings, B.B., Alföldi, J., Wang, Q., Collins, R.L., Laricchia, K.M., Ganna, A., Birnbaum, D.P., et al. (2020). The mutational constraint spectrum quantified from variation in 141,456 humans. Nature *581*, 434–443. https://doi.org/10.1038/s41586-020-2308-7.

3. Medina-Carmona, E., Betancor-Fernández, I., Santos, J., Mesa-Torres, N., Grottelli, S., Batlle, C., Naganathan, A.N., Oppici, E., Cellini, B., Ventura, S., et al. (2019). Insight into the specificity and severity of pathogenic mechanisms associated with missense mutations through experimental and structural perturbation analyses. Hum. Mol. Genet. *28*, 1–15. https://doi.org/10.1093/hmg/ddy323.

4. Malhotra, S., Alsulami, A.F., Heiyun, Y., Ochoa, B.M., Jubb, H., Forbes, S., and Blundell, T.L. (2019). Understanding the impacts of missense mutations on structures and functions of human cancer-related genes: A preliminary computational analysis of the COSMIC Cancer Gene Census. PLoS One *14*, e0219935. https://doi.org/10.1371/journal.pone.0219935.

5. Martínez-Jiménez, F., Muiños, F., Sentís, I., Deu-Pons, J., Reyes-Salazar, I., Arnedo-Pac, C., Mularoni, L., Pich, O., Bonet, J., Kranas, H., et al. (2020). A compendium of mutational cancer driver genes. Nat. Rev. Cancer *20*, 555–572. https://doi.org/10.1038/s41568-020-0290-x.

6. Brandes, N., Goldman, G., Wang, C.H., Ye, C.J., and Ntranos, V. (2023). Genome-wide prediction of disease variant effects with a deep protein language model. Nat. Genet. *55*, 1512–1522. https://doi.org/10.1038/s41588-023-01465-0.

7. Cheng, J., Novati, G., Pan, J., Bycroft, C., Žemgulytė, A., Applebaum, T., Pritzel, A., Wong, L.H., Zielinski, M., Sargeant, T., et al. (2023). Accurate proteome-wide missense variant effect prediction with AlphaMissense. Science *381*, eadg7492. https://doi.org/10.1126/science.adg7492.

8. Gao, H., Hamp, T., Ede, J., Schraiber, J.G., McRae, J., Singer-Berk, M., Yang, Y., Dietrich, A.S.D., Fiziev, P.P., Kuderna, L.F.K., et al. (2023). The landscape of tolerated genetic variation in humans and primates. Science *380*, eabn8153. https://doi.org/10.1126/science.abn8197.

9. Stein, D., Kars, M.E., Wu, Y., Bayrak, Ç.S., Stenson, P.D., Cooper, D.N., Schlessinger, A., and Itan, Y. (2023). Genome-wide prediction of pathogenic gain- and loss-of-function variants from ensemble learning of a diverse feature set. Genome Med. *15*, 103. https://doi.org/10.1186/s13073-023-01261-9.

10. Ostroverkhova, D., Sheng, Y., and Panchenko, A. (2024). Are Next-Generation Pathogenicity Predictors Applicable to Cancer? J. Mol. Biol. *436*, 168644. https://doi.org/10.1016/j.jmb.2024.168644.

11. Fowler, D.M., and Fields, S. (2014). Deep mutational scanning: a new style of protein science. Nat. Methods *11*, 801–807. https://doi.org/10.1038/nmeth.3027.

12. Kinney, J.B., and McCandlish, D.M. (2019). Massively Parallel Assays and Quantitative Sequence-Function Relationships. Annu. Rev. Genomics Hum. Genet. *20*, 99–127. https://doi.org/10.1146/annurev-genom-083118-014845.

13. Tabet, D., Parikh, V., Mali, P., Roth, F.P., and Claussnitzer, M. (2022). Scalable Functional Assays for the Interpretation of Human Genetic Variation. Annu. Rev. Genet. *56*, 441–465. https://doi.org/10.1146/annurev-genet-072920-032107.

14. Fowler, D.M., Adams, D.J., Gloyn, A.L., Hahn, W.C., Marks, D.S., Muffley, L.A., Neal, J.T., Roth, F.P., Rubin, A.F., Starita, L.M., and Hurles, M.E. (2023). An Atlas of Variant Effects to understand the genome at nucleotide resolution. Genome Biol. *24*, 147. https://doi.org/10.1186/s13059-023-02986-x.

15. Bjarnadóttir, T.K., Gloriam, D.E., Hellstrand, S.H., Kristiansson, H., Fredriksson, R., and Schiöth, H.B. (2006). Comprehensive repertoire and phylogenetic analysis of the G protein-coupled receptors in human and mouse. Genomics *88*, 263–273. https://doi.org/10.1016/j.ygeno.2006.04.001.

16. Hauser, A.S., Attwood, M.M., Rask-Andersen, M., Schiöth, H.B., and Gloriam, D.E. (2017). Trends in GPCR drug discovery: new agents, targets and indications. Nat. Rev. Drug Discov. *16*, 829–842. https://doi.org/10.1038/nrd.2017.178.

17. Weis, W.I., and Kobilka, B.K. (2018). The Molecular Basis of G Protein-Coupled Receptor Activation. Annu. Rev. Biochem. *87*, 897–919. https://doi.org/10.1146/annurev-biochem-060614-033910.

18. Jiang, H., Galtes, D., Wang, J., and Rockman, H.A. (2022). G protein-coupled receptor signaling: transducers and effectors. Am. J. Physiol. Cell Physiol. *323*, C731–C748. https://doi.org/10.1152/ajpcell.00210.2022.

19. Heredia, J.D., Park, J., Brubaker, R.J., Szymanski, S.K., Gill, K.S., and Procko, E. (2018). Mapping Interaction Sites on Human Chemokine Receptors by Deep Mutational Scanning. J. Immunol. *200*, 3825–3839. https://doi.org/10.4049/jimmunol.1800343.

20. Park, J., Selvam, B., Sanematsu, K., Shigemura, N., Shukla, D., and Procko, E. (2019). Structural architecture of a dimeric class C GPCR based on co-trafficking of sweet taste receptor subunits. J. Biol. Chem. *294*, 4759–4774. https://doi.org/10.1074/jbc.RA118.006173.

21. Penn, W.D., McKee, A.G., Kuntz, C.P., Woods, H., Nash, V., Gruenhagen, T.C., Roushar, F.J., Chandak, M., Hemmerich, C., Rusch, D.B., et al. (2020). Probing biophysical sequence constraints within the transmembrane domains of rhodopsin by deep mutational scanning. Sci. Adv. *6*, eaay7505. https://doi.org/10.1126/sciadv.aay7505.

22. Jones, E.M., Lubock, N.B., Venkatakrishnan, A.J., Wang, J., Tseng, A.M., Paggi, J.M., Latorraca, N.R., Cancilla, D., Satyadi, M., Davis, J.E., et al. (2020). Structural and functional characterization of G protein-coupled receptors with deep mutational scanning. eLife *9*, e54895. https://doi.org/10.7554/eLife.54895.

23. Howard, M.K., Hoppe, N., Huang, X.-P., Mitrovic, D., Billesbølle, C.B., Macdonald, C.B., Mehrotra, E., Rockefeller Grimes, P., Trinidad, D.D., Delemotte, L., et al. (2025). Molecular basis of proton sensing by G protein-coupled receptors. Cell *188*, 671–687.e20. https://doi.org/10.1016/j.cell.2024.11.036.

24. Pándy-Szekeres, G., Caroli, J., Mamyrbekov, A., Kermani, A.A., Keserű, G. M., Kooistra, A.J., and Gloriam, D.E. (2023). GPCRdb in 2023: state-specific structure models using AlphaFold2 and new ligand resources. Nucleic Acids Res. *51*, D395–D402. https://doi.org/10.1093/nar/gkac1013.

25. Muppidi, J.R., Schmitz, R., Green, J.A., Xiao, W., Larsen, A.B., Braun, S.E., An, J., Xu, Y., Rosenwald, A., Ott, G., et al. (2014). Loss of signalling via Gα13 in germinal centre B-cell-derived lymphoma. Nature *516*, 254–258. https://doi.org/10.1038/nature13765.

26. Muppidi, J.R., Lu, E., and Cyster, J.G. (2015). The G protein-coupled receptor P2RY8 and follicular dendritic cells promote germinal center confinement of B cells, whereas S1PR3 can contribute to their dissemination. J. Exp. Med. *212*, 2213–2222. https://doi.org/10.1084/jem.20151250.

27. Gallman, A.E., Wolfreys, F.D., Nguyen, D.N., Sandy, M., Xu, Y., An, J., Li, Z., Marson, A., Lu, E., and Cyster, J.G. (2021). Abcc1 and Ggt5 support lymphocyte guidance through export and catabolism of S-geranylgeranyl-l-glutathione. Sci. Immunol. *6*, eabg1101. https://doi.org/10.1126/sciimmunol.abg1101.

28. Lu, E., Wolfreys, F.D., Muppidi, J.R., Xu, Y., and Cyster, J.G. (2019). S-Geranylgeranyl-L-glutathione is a ligand for human B cell-confinement receptor P2RY8. Nature *567*, 244–248. https://doi.org/10.1038/s41586-019-1003-z.

29. Lu, E., and Cyster, J.G. (2019). G-protein coupled receptors and ligands that organize humoral immune responses. Immunol. Rev. *289*, 158–172. https://doi.org/10.1111/imr.12743.

30. Lohr, J.G., Stojanov, P., Lawrence, M.S., Auclair, D., Chapuy, B., Sougnez, C., Cruz-Gordillo, P., Knoechel, B., Asmann, Y.W., Slager, S.L., et al. (2012). Discovery and prioritization of somatic mutations in diffuse large B-cell lymphoma (DLBCL) by whole-exome sequencing. Proc. Natl. Acad. Sci. USA *109*, 3879–3884. https://doi.org/10.1073/pnas.1121343109.

CellPress
OPEN ACCESS

31. Sondka, Z., Dhir, N.B., Carvalho-Silva, D., Jupe, S., Madhumita, null, McLaren, K., Starkey, M., Ward, S., Wilding, J., Ahmed, M., et al. (2024). COSMIC: a curated database of somatic variants and clinical data for cancer. Nucleic Acids Res. *52*, D1210–D1217. https://doi.org/10.1093/nar/gkad986.

32. He, Y., Gallman, A.E., Xie, C., Shen, Q., Ma, J., Wolfreys, F.D., Sandy, M., Arsov, T., Wu, X., Qin, Y., et al. (2022). P2RY8 variants in lupus patients uncover a role for the receptor in immunological tolerance. J. Exp. Med. *219*, e20211004. https://doi.org/10.1084/jem.20211004.

33. Cyster, J.G., Shotton, D.M., and Williams, A.F. (1991). The dimensions of the T lymphocyte glycoprotein leukosialin and identification of linear protein epitopes that can be modified by glycosylation. EMBO J. *10*, 893–902. https://doi.org/10.1002/j.1460-2075.1991.tb08022.x.

34. Olvera-León, R., Zhang, F., Offord, V., Zhao, Y., Tan, H.K., Gupta, P., Pal, T., Robles-Espinoza, C.D., Arriaga-González, F.G., Matsuyama, L.S.A.S., et al. (2024). High-resolution functional mapping of RAD51C by saturation genome editing. Cell *187*, 5719–5734.e19. https://doi.org/10.1016/j.cell.2024.08.039.

35. Hsu, C., Nisonoff, H., Fannjiang, C., and Listgarten, J. (2022). Learning protein fitness models from evolutionary and assay-labeled data. Nat. Biotechnol. *40*, 1114–1122. https://doi.org/10.1038/s41587-021-01146-5.

36. Isberg, V., de Graaf, C., Bortolato, A., Cherezov, V., Katritch, V., Marshall, F.H., Mordalski, S., Pin, J.-P., Stevens, R.C., Vriend, G., and Gloriam, D.E. (2015). Generic GPCR residue numbers - aligning topology maps while minding the gaps. Trends Pharmacol. Sci. *36*, 22–31. https://doi.org/10.1016/j.tips.2014.11.001.

37. Gacasan, S.B., Baker, D.L., and Parrill, A.L. (2017). G protein-coupled receptors: the evolution of structural insight. AIMS Biophys. *4*, 491–527. https://doi.org/10.3934/biophy.2017.3.491.

38. Ballesteros, J.A., and Weinstein, H. (1995). [19] Integrated methods for the construction of three-dimensional models and computational probing of structure-function relations in G protein-coupled receptors. In Methods in Neurosciences Receptor Molecular Biology, S.C. Sealfon, ed. (Academic Press), pp. 366–428. https://doi.org/10.1016/S1043-9471(05)80049-7.

39. Barros-Álvarez, X., Nwokonko, R.M., Vizurraga, A., Matzov, D., He, F., Papasergi-Scott, M.M., Robertson, M.J., Panova, O., Yardeni, E.H., Seven, A.B., et al. (2022). The tethered peptide activation mechanism of adhesion GPCRs. Nature *604*, 757–762. https://doi.org/10.1038/s41586-022-04575-7.

40. Nehmé, R., Carpenter, B., Singhal, A., Strege, A., Edwards, P.C., White, C. F., Du, H., Grisshammer, R., and Tate, C.G. (2017). Mini-G proteins: Novel tools for studying GPCRs in their active conformation. PLoS One *12*, e0175642. https://doi.org/10.1371/journal.pone.0175642.

41. Lu, M., and Wu, B. (2016). Structural studies of G protein-coupled receptors. IUBMB Life *68*, 894–903. https://doi.org/10.1002/iub.1578.

42. Olsen, R.H.J., DiBerto, J.F., English, J.G., Glaudin, A.M., Krumm, B.E., Slocum, S.T., Che, T., Gavin, A.C., McCorvy, J.D., Roth, B.L., and Strachan, R.T. (2020). TRUPATH, an open-source biosensor platform for interrogating the GPCR transducerome. Nat. Chem. Biol. *16*, 841–849. https://doi.org/10.1038/s41589-020-0535-8.

43. DiBerto, J.F., Olsen, R.H.J., and Roth, B.L. (2022). TRUPATH: An Open-Source Biosensor Platform for Interrogating the GPCR Transducerome. Methods Mol. Biol. *2525*, 185–195. https://doi.org/10.1007/978-1-0716-2473-9_13.

44. Dixon, A.S., Schwinn, M.K., Hall, M.P., Zimmerman, K., Otto, P., Lubben, T.H., Butler, B.L., Binkowski, B.F., Machleidt, T., Kirkland, T.A., et al. (2016). NanoLuc Complementation Reporter Optimized for Accurate Measurement of Protein Interactions in Cells. ACS Chem. Biol. *11*, 400–408. https://doi.org/10.1021/acschembio.5b00753.

45. Tate, J.G., Bamford, S., Jubb, H.C., Sondka, Z., Beare, D.M., Bindal, N., Boutselakis, H., Cole, C.G., Creatore, C., Dawson, E., et al. (2019). COSMIC: the Catalogue Of Somatic Mutations In Cancer. Nucleic Acids Res. *47*, D941–D947. https://doi.org/10.1093/nar/gky1015.

46. Hawkins-Hooker, A., Kmec, J., Bent, O., and Duckworth, P. (2024). Likelihood-based fine-tuning of protein language models for few-shot fitness prediction and design. Preprint at bioRxiv. https://doi.org/10.1101/2024.05.28.596156.

47. Lafita, A., Gonzalez, F., Hossam, M., Smyth, P., Deasy, J., Allyn-Feuer, A., Seaton, D., and Young, S. (2024). Fine-tuning protein language models with deep mutational scanning improves variant effect prediction. Preprint at arXiv. https://doi.org/10.48550/arXiv.2405.06729.

48. Zhang, N., Bi, Z., Liang, X., Cheng, S., Hong, H., Deng, S., Lian, J., Zhang, Q., and Chen, H. (2022). OntoProtein: Protein Pretraining With Gene Ontology Embedding. Preprint at arXiv. https://doi.org/10.48550/arXiv.2201.11147.

49. Zhang, J., Zhang, D.C., Liang, S., Li, Z., Ying, R., and Shao, J. (2024). Retrieval-Augmented Language Model for Knowledge-aware Protein Encoding. https://openreview.net/forum?id=0MVWOHwHDb, accessed May 2025.

50. Kalifa, D., Singer, U., and Radinsky, K. (2025). GOProteinGNN: Leveraging Protein Knowledge Graphs for Protein Representation Learning. Preprint at arXiv. https://doi.org/10.48550/arXiv.2408.00057.

51. Chen, H., Chen, K., Huang, W., Staudt, L.M., Cyster, J.G., and Li, X. (2022). Structure of S1PR2-heterotrimeric G13 signaling complex. Sci. Adv. *8*, eabn0067. https://doi.org/10.1126/sciadv.abn0067.

52. Billesbølle, C.B., de March, C.A., van der Velden, W.J.C., Ma, N., Tewari, J., Del Torrent, C.L., Li, L., Faust, B., Vaidehi, N., Matsunami, H., and Manglik, A. (2023). Structural basis of odorant recognition by a human odorant receptor. Nature *615*, 742–749. https://doi.org/10.1038/s41586-023-05798-y.

53. Ursu, O., Neal, J.T., Shea, E., Thakore, P.I., Jerby-Arnon, L., Nguyen, L., Dionne, D., Diaz, C., Bauman, J., Mosaad, M.M., et al. (2022). Massively parallel phenotyping of coding variants in cancer with Perturb-seq. Nat. Biotechnol. *40*, 896–905. https://doi.org/10.1038/s41587-021-01160-7.

54. Barsi-Rhyne, B., Manglik, A., and von Zastrow, M. (2022). Discrete GPCR-triggered endocytic modes enable β-arrestins to flexibly regulate cell signaling. eLife *11*, e81563. https://doi.org/10.7554/eLife.81563.

55. Staus, D.P., Wingler, L.M., Choi, M., Pani, B., Manglik, A., Kruse, A.C., and Lefkowitz, R.J. (2018). Sortase ligation enables homogeneous GPCR phosphorylation to reveal diversity in β-arrestin coupling. Proc. Natl. Acad. Sci. USA *115*, 3834–3839. https://doi.org/10.1073/pnas.1722336115.

56. Andrews, S. (2010). FastQC: A Quality Control Tool for High Throughput Sequence Data. Available online.

57. Bushnell, B. BBTools Software Package. Available online.

58. McKenna, A., Hanna, M., Banks, E., Sivachenko, A., Cibulskis, K., Kernytsky, A., Garimella, K., Altshuler, D., Gabriel, S., Daly, M., and DePristo, M. A. (2010). The Genome Analysis Toolkit: a MapReduce framework for analyzing next-generation DNA sequencing data. Genome Res. *20*, 1297–1303. https://doi.org/10.1101/gr.107524.110.

59. Rubin, A.F., Gelman, H., Lucas, N., Bajjalieh, S.M., Papenfuss, A.T., Speed, T.P., and Fowler, D.M. (2017). A statistical framework for analyzing deep mutational scanning data. Genome Biol. *18*, 150. https://doi.org/10.1186/s13059-017-1272-5.

60. Schindelin, J., Arganda-Carreras, I., Frise, E., Kaynig, V., Longair, M., Pietzsch, T., Preibisch, S., Rueden, C., Saalfeld, S., Schmid, B., et al. (2012). Fiji: an open-source platform for biological-image analysis. Nat. Methods *9*, 676–682. https://doi.org/10.1038/nmeth.2019.

61. Goddard, T.D., Huang, C.C., Meng, E.C., Pettersen, E.F., Couch, G.S., Morris, J.H., and Ferrin, T.E. (2018). UCSF ChimeraX: Meeting modern challenges in visualization and analysis. Protein Sci. *27*, 14–25. https://doi.org/10.1002/pro.3235.

62. Meng, E.C., Goddard, T.D., Pettersen, E.F., Couch, G.S., Pearson, Z.J., Morris, J.H., and Ferrin, T.E. (2023). UCSF ChimeraX: Tools for structure building and analysis. Protein Sci. *32*, e4792. https://doi.org/10.1002/pro.4792.

63. Croll, T.I. (2018). ISOLDE: a physically realistic environment for model building into low-resolution electron-density maps. Acta Crystallogr. D Struct. Biol. *74*, 519–530. https://doi.org/10.1107/S2059798318002425.

64. Adams, P.D., Afonine, P.V., Bunkóczi, G., Chen, V.B., Davis, I.W., Echols, N., Headd, J.J., Hung, L.-W., Kapral, G.J., Grosse-Kunstleve, R.W., et al. (2010). PHENIX: a comprehensive Python-based system for macromolecular structure solution. Acta Crystallogr. D Biol. Crystallogr. *66*, 213–221. https://doi.org/10.1107/S0907444909052925.

65. Moriarty, N.W., Grosse-Kunstleve, R.W., and Adams, P.D. (2009). electronic Ligand Builder and Optimization Workbench (eLBOW): a tool for ligand coordinate and restraint generation. Acta Crystallogr. D Biol. Crystallogr. *65*, 1074–1080. https://doi.org/10.1107/S0907444909029436.

66. Emsley, P., and Cowtan, K. (2004). Coot: model-building tools for molecular graphics. Acta Crystallogr. D Biol. Crystallogr. *60*, 2126–2132. https://doi.org/10.1107/S0907444904019158.

67. Williams, C.J., Headd, J.J., Moriarty, N.W., Prisant, M.G., Videau, L.L., Deis, L.N., Verma, V., Keedy, D.A., Hintze, B.J., Chen, V.B., et al. (2018). MolProbity: More and better reference data for improved all-atom structure validation. Protein Sci. *27*, 293–315. https://doi.org/10.1002/pro.3330.

68. Barad, B.A., Echols, N., Wang, R.Y.-R., Cheng, Y., DiMaio, F., Adams, P. D., and Fraser, J.S. (2015). EMRinger: side chain-directed model and map validation for 3D cryo-electron microscopy. Nat. Methods *12*, 943–946. https://doi.org/10.1038/nmeth.3541.

69. Bushnell, B., Rood, J., and Singer, E. (2017). BBMerge - Accurate paired shotgun read merging via overlap. PLoS One *12*, e0185056. https://doi.org/10.1371/journal.pone.0185056.

70. Van der Auwera, G.A., and O’Connor, B.D. (2020). Genomics in the Cloud: Using Docker, GATK, and WDL in Terra (O’Reilly Media).

71. Rives, A., Meier, J., Sercu, T., Goyal, S., Lin, Z., Liu, J., Guo, D., Ott, M., Zitnick, C.L., Ma, J., and Fergus, R. (2021). Biological structure and function emerge from scaling unsupervised learning to 250 million protein sequences. Proc. Natl. Acad. Sci. USA *118*, e2016239118. https://doi.org/10.1073/pnas.2016239118.

STAR★METHODS

KEY RESOURCES TABLE

REAGENT or RESOURCE	SOURCE	IDENTIFIER
Antibodies		
Human TruStain FcX block	Biolegend	Cat# 422302; RRID:AB_2818986
InVivoPlus anti-mouse CD16/CD32 (mouse Fc block)	Bio X Cell	Cat# BE0307; RRID:AB_2736987
Armenian hamster anti-mouse CD3ε biotin (clone 145-2C11)	Biolegend	Cat# 100303, RRID:AB_312668
Syrian hamster anti-mouse anti-CD3ε AF700 (clone 500A2)	Biolegend	Cat# 152316; RRID:AB_2632713
Rat anti-mouse CD4 BV711 (clone GK1.5)	Biolegend	Cat# 100447; RRID:AB_2564586
Rat anti-mouse CD8a BV510 (clone 53-6.7)	Biolegend	Cat# 100752; RRID:AB_2563057
Rat anti-mouse CD11b BV570 (clone M1/70)	Biolegend	Cat# 101233; RRID:AB_10896949
Rat anti-mouse CD35 (CR1) biotin (clone 8C12)	Fisher Scientific	Cat# 553816; RRID:AB_2643602
Mouse anti-mouse CD45.1 APC (clone A20)	Cytek Biosciences	Cat# 20–0453; RRID:AB_2621575
Mouse anti-mouse CD45.1 PE (clone 104)	Biolegend	Cat# 109808; RRID:AB_313445
Rat anti-mouse/human CD45R/B220 BV785 (clone RA3-6B2)	Biolegend	Cat# 103246; RRID:AB_2563256
Hamster anti-mouse CD95 PE-Cy7 (clone Jo2)	BD Biosciences	Cat# 557653; RRID:AB_396768
Rat anti-mouse CD180 (clone RP/14)	BD Biosciences	Cat# 562191; RRID:AB_11153851
Rabbit anti-GFP AF488 (polyclonal)	Thermo Fisher Scientific	Cat# A21311; RRID:AB_221477
Rat anti-GFP AF488 (clone FM2-64G)	Biolegend	Cat # 338007; RRID:AB_2563287
Rat anti-mouse/human GL7 antigen Pacific Blue (clone GL7)	Biolegend	Cat# 144614; RRID:AB_2563292
Rat anti-mouse IgD AF647 (clone 11-26c.2a)	Biolegend	Cat# 405708; RRID:AB_893528
Rat anti-mouse IgD PerCP-Cy5.5 (clone 11-26c.2a)	Biolegend	Cat# 405710; RRID:AB_1575113
Rat anti-OX56 antigen biotin	Contract production Bio X Cell; biotinylated in house	N/A
Rabbit anti-phospho-Akt (Ser473) (clone D9E)	Cell Signaling Technology	Cat# 4060; RRID:AB_2315049
Rabbit anti-phospho-p44/42 MAPK (Erk1/2, Thr202/Tyr204) (clone 197G2)	Cell Signaling Technology	Cat# 4377; RRID:AB_331775
Goat anti-rabbit IgG AF647 (polyclonal)	Thermo Fisher Scientific	Cat# A21245; RRID:AB_2535813
FluoTag-X2 anti-TagFP, AZDye568	NanoTag Biotechnologies	Cat# N0502-AF568; RRID:AB_3075935
Bacterial and virus strains		
Endura electrocompetent cells	Biosearch Technologies	Cat# 60242-2
Chemicals, peptides, and recombinant proteins		
AvrII restriction enzyme	NEB	Cat# R0174S
BmtI-HF restriction enzyme	NEB	Cat# R3658S
PmeI restriction enzyme	NEB	Cat# R0560S
PshAI restriction enzyme	NEB	Cat# R0593S
XhoI restriction enzyme	NEB	Cat# R0146S
T4 DNA polymerase	NEB	Cat# M0203S
Q5 Hot Start High-Fidelity DNA polymerase	NEB	Cat# M0493L

(*Continued on next page*)

Continued

REAGENT or RESOURCE	SOURCE	IDENTIFIER
Lipofectamine 3000 Transfection Reagent	Thermo Fisher Scientific	Cat# L3000015
ViralBoost Reagent	Alstem	Cat# VB100
Lenti-X Concentrator	Takara Bio	Cat# 631232
Fixable Viability Dye eFluor780	Thermo Fisher Scientific	Cat# 65-0865-14
Streptavidin AF647	Thermo Fisher Scientific	Cat# S21374
Streptavidin AMCA	Vector Laboratories	Cat# SA-5008-1
EasySep Streptavidin RapidSpheres	StemCell Technologies	Cat# 50001
Human CXCL12 (SDF1α)	PeproTech	Cat# 300-28A
S-geranylgeranyl-L-glutathione (GGG)	Cayman Chemical; except for Figure 5E for which in house synthesis (Lu et al.[28])	Cat# 9004116
Bovine serum albumin (BSA), fraction V, fatty acid-free	Sigma-Aldrich	Cat# 126575
AMPure XP Beads	Beckman Coulter	Cat# A66380
TransIT-2020 Transfection Reagent	VWR	Cat# 10767-016
Polybrene	Sigma-Aldrich	Cat# TR-1003
Coelenterazine 400a	NanoLight	Cat# 340
Coelenterazine h	Nanolight	Cat# 301
5-fluorouracil	Sigma-Aldrich	Cat# F6627
Collagenase VIII from Clostridium histolyticum	Sigma-Aldrich	Cat# C2139
Mouse serum	Sigma-Aldrich	Cat# M5905
Doxycycline hyclate	Sigma Aldrich	Cat# D9891
Tris(2-carboxyethyl)phosphine	Fisher Scientific	Cat# AC363830010
Pierce protease inhibitor tablet	Thermo Fisher Scientific	Cat# A32963
Lauryl maltose neopentyl glycol (L-MNG)	Anatrace	Cat# NG310
ATP	Fisher Scientific	Cat# AC102800100
Cholesteryl hemisuccinate	Steraloids	Cat# C6823
Glyco-diosgenin (GDN)	Anatrace	Cat# GDN101
n-dodecyl-β-*d*-maltopyranoside (DM)	Anatrace	Cat# D310
Lambda phosphatase	NEB	Cat# P0753S
Calf intestinal phosphatase	NEB	Cat# M0290
Antarctic phosphatase	NEB	Cat# M0289S
Leukotriene C4	Cayman Chemical	Cat# 20210
S-octane-L-glutathione	In house synthesis	N/A
S-hexadecane-L-glutathione	In house synthesis	N/A
S-isoprenyl-L-glutathione	In house synthesis	N/A
S-farnesyl-L-glutathione	In house synthesis	N/A
Farnesol	Sigma-Aldrich	Cat# F203
Triphenylphosphine	Sigma-Aldrich	Cat# T84409
Tetrabromomethane (carbon tetrabromide)	Sigma-Aldrich	Cat# C11081
N-hexane	Sigma-Aldrich	Cat# 296090
L-glutathione	Sigma-Aldrich	Cat# G4251
1-bromooctane	Sigma-Aldrich	Cat# 152951
3,3-demethylallyl bromide	Sigma-Aldrich	Cat# 249904
**Critical commercial assays**		
Quick Ligation Kit	NEB	Cat# M2200S
Nucleospin Gel & PCR Clean-up Mini Kit	Macherey-Nagel	Cat# 740609.50
Gibson Assembly Master Mix	NEB	Cat# E2611S

(*Continued on next page*)

Continued

REAGENT or RESOURCE	SOURCE	IDENTIFIER
QIAquick Gel Extraction Kit	Qiagen	Cat# 28704
HiFi DNA Assembly Master Mix	NEB	Cat# E2621S
QIAprep Spin Miniprep Kit	Qiagen	Cat# 27104
HiSpeed Plasmid Midi Prep Kit	Qiagen	Cat# 12643
Quick-DNA Miniprep Plus Kit	Zymo Research	Cat# D3025
QIAEX II Gel Extraction Kit	Qiagen	Cat# 20021
Qubit dsDNA HS Quantification Assay Kit	Thermo Fisher Scientific	Cat# Q32851
Q5 Site-directed Mutagenesis Kit	NEB	Cat# E0554S
Nextera XT DNA Library Preparation Kit	Illumina	Cat# FC-131-1096
TapeStation High Sensitivity D5000 Ladder; Reagents; ScreenTape	Agilent	Cat# 5067–5594; 5067–5593; 5067-5592
RhoA G-LISA GTPase Activation Assay Kit (colorimetric)	Cytoskeleton	Cat# BK124
Total RhoA ELISA Kit	Cytoskeleton	Cat# BK150
ExpiFectamine 293 Transfection Kit	Thermo Fisher Scientific	Cat# A14525
Qubit Protein Assay Kit	Thermo Fisher Scientific	Cat# Q33211
**Deposited data**		
ESM1b human missense variant scores	Brandes et al.[6]	https://huggingface.co/spaces/ntranoslab/esm_variants
gnomAD v4.0.0	Karczewski et al.[2]	https://gnomad.broadinstitute.org/; RRID: SCR_014964
COSMIC	Tate et al.[45]	https://cancer.sanger.ac.uk/cosmic/login; RRID:SCR_002260
Raw and analyzed P2RY8 DMS data	This paper	NCBI SRA: PRJNA1179413 (RRID: SCR_004891)
GGG-bound P2RY8-miniG$_{13}$ structure coordinates	This paper	PDB: 9ECJ; EMD-47912; EMD-47914
Gα13 structure coordinates	Chen et al.[51]	PDB: 7T6B
Gβ1γ2 structure coordinates	Billesbølle et al.[52]	PDB: 8F76
GPR68 deep mutational scan fitness score	Howard et al.[23]	MAVEdb: 00001207
**Experimental models: Cell lines**		
OCI-Ly8	Cell line was previously obtained from other laboratories and further authentication was not performed	RRID:CVCL_8803
HEK293T	Cell line was previously obtained from other laboratories and further authentication was not performed	RRID:CVCL_0063
HEK293T Platinum-E (Plat-E) Retroviral Packaging Line	Gift from S.Schwab, NYU	Cell Biolabs, Cat# RV101
WEHI-231	Cell line was previously obtained from other laboratories and further authentication was not performed	RRID:CVCL_0577
Lenti-X 293T cell Line	Takara Bio	Cat# 632180
Expi293F TetR inducible cells	Thermo Fisher Scientific	Cat# A39241
Spodoptera frugiperda Sf9 insect cells	Expression Systems	Cat# 94-001F
Trichoplusia ni Hi5 insect cells	Expression Systems	Cat# 94-002F
**Experimental models: Organisms/strains**		
Mouse: C57BL/6J	The Jackson Laboratory	RRID:IMSR_JAX:000664
Mouse: CD45.1 B6: B6.SJL-Ptprc^{a} Pepc^{b}/BoyJ	The Jackson Laboratory	RRID:IMSR_JAX:002014

(*Continued on next page*)

Continued

REAGENT or RESOURCE	SOURCE	IDENTIFIER
Mouse: CD45.1 B6: B6.SJL-Ptprc^{a}Pepc^{b}/BoyCrCrl	NCI Mouse Repository	RRID:IMSR_CRL: 564
Oligonucleotides		
Variant pool, see Table S1	Twist	N/A
For cloning, PCR, etc. see Table S4	Integrated DNA Technologies	N/A
Recombinant DNA		
p_sc_eVIP	Ursu et al.[53]	RRID:Addgene_168174
pMD2.G	Gift from Didier Trono (Addgene #12259)	RRID:Addgene_12259
psPAX2	Gift from Didier Trono (Addgene #12260	RRID:Addgene_12260
MSCV-PRL-OX56-P2RY8-IRES-GFP	Lu et al.[28]	N/A
PRL-OX56-P2RY8-P2A-eGFP (POP2E)	This paper	N/A
POP2-AvrII-E	This paper	N/A
POP2E_V29W	This paper	N/A
POP2E_A34F	This paper	N/A
POP2E_S44C	This paper	N/A
POP2E_T68K	This paper	N/A
POP2E_F103L	This paper	N/A
POP2E_Y104I	This paper	N/A
POP2E_G124E	This paper	N/A
POP2E_K180I	This paper	N/A
POP2E_W181D	This paper	N/A
POP2E_W181M	This paper	N/A
POP2E_W181S	This paper	N/A
POP2E_L220Y	This paper	N/A
POP2E_G237N	This paper	N/A
POP2E_A240T	This paper	N/A
POP2E_T330N	This paper	N/A
pcDNA5/FRT/TO-GAlpha13-RLuc8	Olsen et al.[42]	RRID:Addgene_140986
pcDNA3.1-Beta3	Olsen et al.[42]	RRID:Addgene_140988
pcDNA3.1-GGamma9-GFP2	Olsen et al.[42]	RRID:Addgene_140991
β2AR-LgBiT	Barsi-Rhyne et al.[54]	N/A
β-Arr2-SmBiT	Barsi-Rhyne et al.[54]	N/A
P2RY8-LgBiT	This paper	N/A
P2RY8-L220Y-LgBiT	This paper	N/A
P2RY8-K180I-LgBiT	This paper	N/A
P2RY8-W181M-LgBiT	This paper	N/A
MSCV-P2RY8-IRES-GFP	Lu et al.[28]	N/A
MSCV-P2RY8-IRES-TagBFP	This paper	N/A
MSCV-P2RY8(K180I)-IRES-GFP	This paper	N/A
MSCV-P2RY8(W181M)-IRES-GFP	This paper	N/A
MSCV-EV-GFP	Lu et al.[28]	N/A
pVLDual-GBeta1-GGamma2	Billesbølle et al.[52]	N/A
pcDNA-Zeo-TetO	Staus et al.[55]	N/A
pcDNA-Zeo-TetO-P2RY8-miniGs	This paper	N/A
Software and algorithms		
FlowJo v10	FlowJo	https://flowjo.com
Prism v10	GraphPad	https://graphpad.com

(*Continued on next page*)

Continued

REAGENT or RESOURCE	SOURCE	IDENTIFIER
BD FACSDiva	BD Biosciences	https://www.bdbiosciences.com/en-us/products/software/instrument-software/bd-facsdiva-software
FastQC v0.12.0	Andrews, S.[56]	https://www.bioinformatics.babraham.ac.uk/projects/fastqc/
BBTools	Bushnell, B.[57]	https://archive.jgi.doe.gov/data-and-tools/software-tools/bbtools/
GATK	McKenna et al.[58]	https://gatk.broadinstitute.org
Enrich2	Rubin et al.[59]	https://enrich2.readthedocs.io/
R v4.3.0	R Project	https://www.r-project.org/
RStudio Desktop	Posit	https://posit.co/download/rstudio-desktop/
tidyverse v2.0.0	https://www.tidyverse.org/	https://www.tidyverse.org/
Python v3.1	https://python.org	https://python.org
Jupyter Notebook	Jupyter Project	https://jupyter.org
torch	https://pytorch.org	https://pytorch.org
pandas	https://pandas.pydata.org/	https://pandas.pydata.org/
numpy	https://numpy.org	https://numpy.org
scipy	https://scipy.org	https://scipy.org
sklearn	https://scikit-learn.org	https://scikit-learn.org
ZEN Microscopy Software	Zeiss	https://www.zeiss.com
Fiji	Schindelin et al.[60]	https://imagej.net/software/fiji/
cryoSPARC	Structura Biotechnology	https://cryosparc.com
ChimeraX	Goddard et al.[61]; Meng et al.[62]	https://www.cgl.ucsf.edu/chimerax/
ISOLDE	Croll, TI.[63]	https://tristanic.github.io/isolde/
Phenix v1.20	Adams et al.[64]	https://www.phenix-online.org/
eLBOW extension	Moriarty et al.[65]	https://www.phenix-online.org/documentation/reference/elbow.html
Coot v0.8.9.2	Emsley et al.[66]	https://www2.mrc-lmb.cam.ac.uk/personal/pemsley/coot/
Molprobity v4.5	Williams et al.[67]	http://molprobity.biochem.duke.edu/
EMRinger	Barad et al.[68]	https://github.com/fraser-lab/EMRinger
Inkscape	https://inkscape.org/; https://gitlab.com/inkscape/inkscape	https://inkscape.org/; https://gitlab.com/inkscape/inkscape
**Other**		
Transwell Multiple Well Plate with Permeable Polycarbonate Membrane Inserts	Fisher Scientific	Cat# 07-200-149
Sheep red blood cells (unpooled, sheep #257)	Alsevers	Cat# 38112
M1-FLAG-antibody conjugated CNBR-sepharose	In house production	N/A
HisPur Ni-NTA resin	Thermo Fisher Scientific	Cat# 88221
Mono Q 4.6/100 PE column	Cytiva	Cat# 17-5179-01
Superdex 200 Increase 10/300 GL size exclusion chromatography column	Cytiva	Cat# 28990944
Holey Gold Supports: UltraAUFoil (R 1.2/1.3) Au300 mech	Quantifoil Micro Tools	N/A
FACS Symphony A1 Cell Analyzer	BD Biosciences	N/A
FACSAria Fusion Cell Sorter	BD Biosciences	N/A
NovaSeq X Plus	Illumina	N/A
Titan Krios	Thermo Fisher Scientific	N/A
Code used in DMS analysis	This paper	https://doi.org/10.5281/zenodo.15811041

EXPERIMENTAL MODEL AND SUBJECT DETAILS

Animals

Mice used for B cell transfers were C57BL/6J bred in an internal colony and used at 8 to 12 weeks of age. For chimeras, donor bone marrow was obtained from C57BL/6J bred internally or purchased from JAX; recipients were CD45.1 B6 mice, bred internally from founders ordered from JAX or purchased from the National Cancer Institute at Charles River at age 7 to 8 weeks, with balanced distribution of experimental groups across these two backgrounds. Mice of both sexes were used. Mice were allocated to control and experimental groups randomly, and sample sizes were chosen based on previous experience and available co-caged littermates. Animals were housed in a pathogen-free environment in the Laboratory Animal Resource Center at UCSF, and all experiments adhered to ethical principles and guidelines that were approved by the Institutional Animal Care and Use Committee.

Cell lines

HEK 293T (293T), WEHI-231, and parent OCI-Ly8 (Ly8) cell lines were previously obtained from other laboratories and further authentication was not performed. The cell lines were not tested for Mycoplasma contamination. P2RY8 KO Ly8 cells were previously generated as described.[28] The culture medium for Ly8 and WEHI-231 cells was RPMI-1640, 10% FBS, 1x GlutaMax, 10 mM HEPES, 55 μM β-mercaptoethanol, and 50 IU mL^{-1} penicillin/streptomycin. Lenti-X 293T cells (Takara) were used to generate lentivirus for transduction of human cells. The culture medium for Lenti-X was DMEM, 10% FBS, 1x GlutaMax, 1 mM sodium pyruvate, 1x MEM non-essential amino acids, 10 mM HEPES, and 50 IU mL^{-1} penicillin/streptomycin. Plat-E 293T (Plat-E) cells (Cell Biolabs) were used to generate retrovirus for transduction of mouse cells. The culture medium for Plat-E and 293T cells was DMEM, 10% FBS, 1x GlutaMax, 10 mM HEPES, and 50 IU mL^{-1} penicillin/streptomycin. All cells were maintained in a 37°C humidified incubator, 5% CO_2. Several cell lines were used as expression systems to obtain components for GPCR complex for cryo-EM study. Expi293F inducible-TetR cells were obtained from Thermo Fisher Scientific and *Spodoptera frugiperda* Sf9 and *Trichoplusia ni* Hi5 insect cells were obtained from Expression Systems. Each cell line was cultured under their recommended conditions with any specific modifications noted in the purification method details.

METHOD DETAILS

Variant pool generation

The vector for library transduction was created through modification of p_sc-eVIP (Gift from Jesse Boehm, James T. Neal, and Aviv Regev; Addgene plasmid # 168174).[53] First we removed the puromycin resistance gene by digestion with XhoI, gel purification (Macherey-Nagel Nucleospin kit), blunt end formation withT4 DNA polymerase (NEB), and ligation with Quick Ligase (NEB), transformation into chemically competent bacteria, and plasmid isolation and screening. We then excised P53 and replaced it with PRL-OX56-P2RY8-P2A-GFP. Specifically, two separate PCR reactions were performed using Q5 polymerase (NEB) on MSCV-PRL-OX56-P2RY8-IRES-GFP plasmid (previously described[28]) to produce PRL-OX56-P2RY8-P2A and P2A-GFP fragments with appropriate homology arms for Gibson assembly and thereby cloned into BmtI and PmeI digested plasmid backbone using Gibson assembly kit (NEB). (See Table S4 for oligonucleotides used in PCRs.) This resulting plasmid is termed POP2E.

The variant oligo library was obtained as Site Saturation Variant Library from Twist (Table S1). The synthesized DNA consisted of the coding sequence for P2RY8 amino acids 2–358 (all codons excepting start and stop codons) along with preceding 35 bp (corresponding to OX56 tag and 5 nucleotides in preceding linker) and succeeding 35 bp (corresponding to a portion of P2A sequence). The pool was designed to include all possible missense mutations (a single codon for a given missense amino acid if multiple codons were possible), along with a synonymous codon for all amino acids for which multiple codons exist (that is, all except methionine and tryptophan). The Q5 site-directed mutagenesis kit (NEB) was used to modify POP2E by inserting a CTA between P2RY8 and P2A, producing a AvrII restriction site. This new construct was digested with PshAI and AvrII, the non-P2RY8 fragment isolated by gel cleanup (Qiagen Qiaquick Gel Extraction kit), and the oligo pool inserted using using HiFi Assembly kit (NEB): 0.0625 pmol variant oligo library and 0.25 pmol of backbone were combined with 2x master mix for 60 min at 50°C. The reaction was cooled on ice then dialyzed for 1 h on a 0.025 μm MCE membrane (Millipore) floating on ultra-distilled water. Added 2 μL of dialyzed assembly reaction to 50 μL of Endura electrocompetent cells (Biosearch Technologies), incubated on ice for 15 min, aliquoted equally to two 0.1 cm cuvettes (Bio-Rad), and electroporated with 1.8 kV, 10 μF, 600 Ω pulse. This was combined with pre-warmed SOC medium, shaken at 37°C for 1 h, and spread across two 25 cm × 25 cm LB ampicillin plate, with the exception of a small volume used in serial dilutions to determine transformation efficiency. Based on these serial dilutions, the large plates had a total of 1.67 x 10^6 transformants, for an average of 233 transformants per variant. After overnight growth, colonies were scraped from the plates, pooled, divided in six, processed using HiSpeed Plasmid Midi Prep kit (Qiagen), and repooled.

To produce lentivirus, Lenti-X cells were plated into two 10 cm plates, with transfection the following day with cells ~85% confluent. To 3 mL of Opti-MEM, added 90 μL of lipofectamine 3000; to another 3 mL Opti-MEM added 13 μg of variant library plasmid, 14 μg psPAX2 (gift from Didier Trono, Addgene #12260), 3.5 μg pMD2.G (gift from Didier Trono, Addgene #12259), and 80 μL of P3000 reagent. Combined these after 5 min room temperature incubation. After 25 min further incubation at room temperature, removed culture medium from the Lenti-X plates, then added 5 mL of complete Opti-MEM (Opti-MEM with, 5% FBS, 1x GlutaMax, 1 mM sodium pyruvate, and 1x MEM non-essential amino acids) and 3 mL of the lipofectamine-DNA mixture to each plate.

Six hours later removed media and replaced with 6 mL of complete Opti-MEM plus 50 IU mL^{-1} penicillin/streptomycin and 1:500 ViralBoost (Alstem). After 24 h, the media was collected, passed through a 0.45 μm filter, and concentrated 20x using Lenti-X concentrator (Takara) and centrifugation. Four such batches of lentivirus were produced for use in the screen.

Variant pool screening

The screen was performed using Ly8 cells in which P2RY8 knockout by CRISPR-Cas9 had previously been performed.[28] For each iteration of the screen, 7 x 10^6 cells were transduced by resuspending cells at 1 x 10^6 cells mL^{-1} in culture medium and 750 μL of lentivirus diluted in Opti-MEM, such that ~30% of cells were transduced (range 25–34%), corresponding to an average of 294 transduced cells per variant. Post-transduction, the cells was passaged as needed to maintain a cell concentration of 1.5 x 10^5 to 1.5 x 10^6 cells mL^{-1} with a total of at least 3 x 10^6 transduced (GFP^+) cells. The four replicates for each assay (surface expression, migration, and proliferation) were drawn from seven independent transductions using four batches of lentiviral library.

Sample collection for surface expression assay occurred 5 days after transduction for 3 replicates and 7 days after transduction for 1 replicate. Cells were washed with FACS buffer (1x PBS, 2% FBS, 1 mM EDTA), stained for 30 min on ice with 1:100 TruStain FcX block (Biolegend) and 1:200 biotinylated anti-OX56 (Bio X Cell) at 40 x 10^6 cells mL^{-1}, washed with FACS buffer, stained for 30 min on ice with 1:400 streptavidin-AF647 (Thermo Fisher Scientific), with e780 fixable viability dye (Thermo Fisher Scientific) added to 1:1500 for last 10 minues, washed with FACS buffer, resuspended in FACS buffer, and sorted using FACSAriaFusion. Gating was FSC-A x SSC-A, singlets by FSC-A x FSC-W, live cells by FSC-A x e780, GFP+, and four bins of OX56 (AF647) expression, containing 20%, 30%, 30%, 20% (Figure S1B). Cells were sorted into 25% FBS in PBS at 4°C. Cells were washed, pelleted, and frozen at −80°C until DNA extraction for processing and sequencing. An average of 2 x 10^6 cells per bin per sort were collected.

The collection for proliferation analysis occurred at 3, 5, and 12 days after transduction for all four replicates as well as 7 days after for two replicates and 8 days after for one replicate. (The Enrich2 algorithm can accommodate and account for these differences.) The proportion of GFP^+ cells was determined by flow cytometry of a small aliquot at the time of collection. Cell aliquots containing an average of 2.3 x 10^6 GFP^+ cells (range 1.5–3.1 x 10^6) were washed with PBS, pelleted, and frozen at −80°C until DNA extraction for processing and sequencing.

Migration assays occurred 5 days (one replicate), 7 days (two replicates), or 8 days (one replicate) after transduction. In each case, 80 x 10^6 cells were washed with migration medium (RPMI, 0.5% fatty acid-free bovine serum albumin, 1x penicillin/streptomycin), resuspended in migration medium at 1 x 10^7 cells mL^{-1} and resensitized for 15 min at 37°C. Migration medium containing 100 ng mL^{-1} recombinant human CXCL12 (PeproTech) and 100 nM GGG (Cayman Chemical) was prepared and 600 μL added to each of 80 wells in 24-well tissue culture plates. Transwell inserts (6 mm, 5 μM pore size, Corning) were placed in each well, after which 100 μL resensitized cells (1 x 10^6 cells) were added to the upper chamber. The cells were placed in 37°C, 5% CO_2 incubator for 3 h to allow migration. The transwells were then removed and the cells in the bottom wells were pooled and counted. A small aliquot was also analyzed by flow cytometry to determine the proportion of GFP^+ cells. The total number of migrated cells ranged from 10 to 21 x 10^6 cells. In addition, an aliquot containing 2-3 x 10^6 GFP^+ cells from the pre-migration input population was also separately collected for each migration. Cells were washed with PBS, pelleted, and frozen at −80°C until DNA extraction for processing and sequencing.

Sequencing library preparation

Library preparation and sequencing was performed as two batches, with two replicates for each condition per batch. Genomic DNA was isolated using Quick-DNA Miniprep Plus kit (Zymo) per its instructions. DNA was quantified using Qubit (Thermo Fisher Scientific). Initial PCR amplification reactions were set up to amplify P2RY8 coding sequence plus ~150 flanking bp on each end. Each reaction was 50 μL using Q5 Hot Start High-Fidelity Polymerase (NEB), including 0.5 μL polymerase, 10 μL 5x buffer, 10 μL high GC enhancer, 0.5 μL 20 μM forward primer, 0.5 μL 20 μM reverse primer, and 27.5 μL of DNA, up to 1500 ng of DNA per reaction. When possible, number of individual PCR reactions per sample was set to have total template equivalent to 150 transduced cells per variant (1.057 x 10^6 transduced cells total); achieved for 26 of 39 samples; variant coverage for the remaining samples was 57x, 66x, 83x, 100x (four samples), 104x, 106x, 120x, 124x, 145x, and 146x. PCR conditions were 98°C × 2 min, 19 cycles of (98°C × 10 s, 70°C × 15 s, 72°C × 45 s), and 72°C × 2 min. After the PCR was performed, all individual PCR reactions for a given template were pooled. Approximately 5–10% (depending on total number of reactions) of each pooled reaction was loaded onto agarose gel, band of desired size verified using gel electrophoresis, and band then cut out and PCR product isolated using Qiaex II kit (Qiagen). DNA was quantified using Qubit. DNA was diluted as needed and 1 ng was tagmented using Nextera XT Library Prep kit (Illumina) per its instruction, including 12 cycle PCR for adding Illumina adapter sequences. DNA clean up performed using AMPure XP beads (Beckman) using 0.75x volume (to remove fragments <300 bp). Quantification and QC was performed using Qubit and gel electrophoresis including TapeStation high-sensitivity electrophoresis (Agilent). Samples were diluted and pooled in equimolar fashion to 10 nM total. PE300 sequencing was performed on Illumina NovaSeq X Plus at UCSF CAT.

Sequencing analysis

Fastq files were obtained from UCSF CAT. There was a mean of 6.51 x 10^7 paired sequences per sample, SD 1.02 x 10^7, max 8.60 x 10^7, min 3.72 x 10^7. Overall quality was evaluated using FastQC[56] (version 0.12.0) (http://www.bioinformatics.babraham.ac.uk/projects/fastqc/). Adapter trimming then performed using BBDuk function of BBTools[57,69] (version 38.18) (https://sourceforge.net/projects/bbmap/). Paired reads were error corrected using BBMerge (also part of BBTools). Reads were then mapped to P2RY8

amplicon reference sequence using BBMap (also part of BBTools). Variants were then called in the mapped SAM file using AnalyzeSaturationMutagenesis function within GATK[58,70] (version 4.5.0.0) (https://gatk.broadinstitute.org/hc/en-us). The utilized output of this analysis was a table containing the read count for each codon for each position in the coding sequence of P2RY8. These tables were then processed using R to filter for only the codons specifically used by Twist in creating the variant oligo pool and formatted as required for analysis by Enrich2[59] (version 1.3.1) (https://github.com/FowlerLab/Enrich2/). Enrichment scores were calculated from the processed files, looking at all four replicates concurrently, by weighted least squares method for surface expression and proliferation and log ratios for migration (since only two points). For each sample, the geometric mean of the counts for the 338 synonymous variants had been calculated and was used as the sample WT count for normalization within Enrich2. The Enrich2 output was further processed in R, including determination of z-scores for expression, migration, and proliferation for all missense variants based on the standard deviation of the Enrich2 scores for the synonymous variants. Expression-adjusted migration score calculated as follows: In R, used lm function to generate best-fit line for expression by migration Enrich2 scores (not z-scores) from synonymous variant data; determined residual for each missense variant score with respect to this line; this value is the expression-adjusted migration score. For position data, the mean expression-adjusted migration score is simply the mean of the score for all missense variants at that position. Analogous method used to determine expression-adjusted proliferation scores and migration-adjusted proliferation scores. High expression-adjusted migration, high expression-adjusted proliferation score, and high migration-adjusted proliferation score positions were defined as being greater than twice the interquartile range above the median. Sequencing data from the deep mutational scan were deposited in the NCBI Sequence Read Archive under BioProject PRJNA1179413 and publicly available. Variant counts and Enrich2 scores are in Table S2. Scripts used for analysis are available at Github at https://github.com/yelabucsf/P2RY8_DMS and on Zenodo at https://doi.org/10.5281/zenodo.15811041.

ESM1b fine-tuning using P2RY8 DMS data

ESM1b takes a single amino acid sequence of length L as input and outputs a 20 x L matrix of log likelihood ratios (LLR) corresponding to the predicted effects of all single amino acid substitutions.[6,71] To improve the predictive power of ESM1b on P2RY8, here we fine-tune the model on a randomly chosen subset of the DMS data (e.g., expression z-scores of k variants per position) and use the rest of the DMS data for performance evaluation (test set). This process is repeated N = 50 times for each experimental measurement (expression, migration and proliferation) and each training set size with k = 2, 3, 5, 7, 9 and 11 variants per position.

Fine-tuning was performed by updating the parameters of the ESM1b model to minimize a masked mean-squared error loss computed between the sampled experimental values and the corresponding model predictions. Before calculating the loss, the sampled experimental values were transformed to match the mean, variance, and the sign of the P2RY8 zero-shot LLRs to enable faster convergence and avoid major changes to the language model logit distribution during training. We further restricted optimization to the language model's final layers, comprising the LM head and the last three embedding layers ($\sim$60M trainable parameters), to preserve the broader contextual knowledge acquired during self-supervised pre-training while enabling adaptation to the experimental data.

Model optimization was carried out using an AdamW optimizer (with a learning rate of 1e$-$5 and weight decay of 0.01) alongside a linear warmup schedule for a maximum of 300 optimization steps. To avoid overfitting to the sampled experimental data, we implemented an early stopping strategy. Specifically, for each training set size, we fine-tuned the model on k-1 variants per position and used the remaining one variant per position for validation. Throughout training, the validation set was used to monitor model performance (Spearman correlation between model predictions and experimental measurements), and training was stopped if the validation correlation did not improve over a predefined number of steps (patience = 30 steps).

After fine-tuning, the updated model generated revised LLR predictions for all P2RY8 variants. To further refine these predictions, we incorporated a ridge regression step that combined one-hot encoded sequence features with the fine-tuned model scores. This final regression model was trained on the same set of k experimental variants per position (including variants from both training and validation sets) without transforming the experimental values, to provide predictions on the experimentally measured scale (e.g., P2RY8 expression z-scores in the case of the expression DMS screen). We also implemented the augmented one-hot encoded ridge regression model previously described,[35] which relies solely on zero-shot model predictions (AlphaMissense and original ESM1b scores), as a baseline to evaluate the performance gains obtained by fine-tuning ESM1b using P2RY8 DMS data.

Finally, we tested whether ESM1b fine-tuning on P2RY8 DMS data can improve variant effect predictions for GPR68, a related GPCR with available DMS data. For this task, we fine-tuned ESM1b on P2RY8 as above, using all experimental DMS data (expression z-scores) for training and tested performance on GPR68 (surface expression and ph 5.5 signaling). We tested the performance of two fine-tuned models, one solely relying on the P2RY8 validation set for early stopping and a second one with a hard stop at 50 optimization steps to avoid overfitting. Overall both models generate similar variant effect scores (LLR) when tested on GPR68, with the latter providing a small increase in Spearman correlation compared to the original (zero-shot) ESM1b model.

Individual variant validation

Specific variants of interest were produced in the POP2E (WT P2RY8) vector by site-directed mutagenesis (Q5 Site-Directed Mutagenesis Kit, NEB) (See Table S4 for specific oligonucleotides used). Lentivirus was produced using Lenti-X by same approach as for lentiviral pool (described above) but scaled down to 1–2 wells in a 6-well plate. Ly8 cells were transduced by same approach as lentiviral pool, again scaled down to 1 x 10^5 cells in 100 μL in a well in a 96-well plate per transduction.

For surface expression, cells were assessed 3 days after transduction. Cells were washed with FACS buffer, stained for 30 min on ice with 1:100 TruStain FcX block (Biolegend) and 1:200 biotinylated anti-OX56 (Bio X Cell), washed twice, stained for 30 min on ice in the dark with 1:250 streptavidin-AF647 (Thermo Fisher Scientific), washed, stained for 10 min on ice in dark with 1:1500 e780 fixable viability dye (Thermo Fisher Scientific), washed, and resuspended and analyzed on a FACSymphony (BD). An aliquot of untransduced Ly8 cells were used in each experiment to set the GFP^+ gate.

For proliferation, transduced cells were maintained in wells of a 24-well tissue culture plate. The ratio of GFP^+ to GFP^- cells was tracked over time by performing flow cytometric analysis of an aliquot at 3, 5, 7, 10, 13, and 16 days after transduction. Cells were passaged at those same time points.

For migration, cells were assayed 5–7 days after transduction. Cells were washed with migration medium (RPMI, 0.5% fatty acid free BSA (Sigma-Aldrich), 10 mM HEPES, and 50 IU mL^{-1} penicillin/streptomycin), resuspended at 2 x 10^6 cells mL^{-1} and resensitized for 10 to 15 min at 37°C. Recombinant human CXCL12 (Peprotech) was diluted to 100 ng mL^{-1} in migration medium. GGG was diluted to various concentrations in the CXCL12-containing migration medium; DMSO (the vehicle for the GGG stock solution) was also added to the CXCL12 only condition at a dilution equivalent to the highest GGG concentration used in that experiment. We added 600 μL of these mixtures to a 24-well tissue culture plate. Transwell filters (6 mm insert, 5 μm pore size, Corning) were placed on top of each well, and 100 μL of resensitized cells (2 x 10^5 cells) was added to the transwell insert. The plate was placed in a 37°C, 5% CO_2 incubator, and the cells were allowed to migrate for 3 h, after which the cells in the bottom well were counted by flow cytometry. To determine the degree of migration inhibition, the number of GFP^+ cells that migrated in each well was normalized to GFP^- cells, and this ratio in turn compared to that for CXCL12 alone.

Proliferation assay with exogenous GGG

P2RY8 KO Ly8 cells were transduced as described in individual variant validation above. Starting on day 3, GGG (or DMSO as a control) was added to the culture media each day to the final desired concentration (ranging from 1 nM to 1 μM GGG, or 1:1000 DMSO). The ratio of GFP^+ to GFP^- cells was tracked over time by flow cytometry at 3, 5, 7, and 10 days after transduction. Cells were passaged at each of those time points as well.

Candidate ligand migration assay

P2RY8-expressing WEHI-231 cells were produced as previously described.[28] In brief, P2RY8 had been cloned into the murine stem cell virus (MSCV)-GFP retroviral vector. The retrovirus was produced using Plat-E cell line. WEHI-231 cells were transduced in a 6 well plate with retroviral supernatant, centrifuged at 1,340 x g for 2 h at room temperature. The supernatant was removed and standard WEHI-231 culture media replaced. This spinfection was repeated 24 h later.

Post-transduction cells were washed with migration medium (RPMI, 0.5% fatty acid free BSA (Sigma-Aldrich), 10 mM HEPES, and 50 IU mL^{-1} penicillin/streptomycin), resuspended at 2 x 10^6 cells mL^{-1} and resensitized for 10 to 15 min at 37°C. Recombinant human CXCL12 (Peprotech) was diluted to 50 ng mL^{-1} in migration medium. GGG or other candidate ligands were diluted to various concentrations in the CXCL12-containing migration medium. We added 600 μL of these mixtures to a 24-well tissue culture plate. Transwell filters (6 mm insert, 5 μm pore size, Corning) were placed on top of each well, and 100 μL of resensitized cells (2 x 10^5 cells) was added to the transwell insert. The plate was placed in a 37°C, 5% CO_2 incubator, and the cells were allowed to migrate for 3 h, after which the cells in the bottom well were counted by flow cytometry. To determine the degree of migration inhibition, the number of GFP+ cells that migrated in each well was normalized to GFP- cells, and this ratio in turn compared to that for CXCL12 alone.

Phospho-flow

P2RY8 KO Ly8 cells were transduced with variants of interest as described in individual variant validation above. Phospho-flow analyses were performed 5 to 7 days after transduction. Cells were washed in migration medium (RPMI, 0.5% fatty-acid free BSA, 10 mM HEPES, and 50 IU mL^{-1} penicillin/streptomycin). They were then resuspended in migration medium at 5 x 10^6 cells mL^{-1} and resensitized at 37°C for 12 min. At that time 100 μL (5 x 10^5) cells were diluted to 200 μL in 5 mL polystyrene round bottom tube with indicated combinations of 100 nM GGG and 100 ng mL^{-1} CXCL12 and incubated in 37°C water bath for 5 min. Afterward, 22 μL of 16% paraformaldehyde was added to each tube and cells fixed at room temperature for 10 min, centrifuged, supernatant removed, and 1 mL of cold methanol added while vortexing. The samples were placed at −20°C for 1–2 nights (consistent within given experiment). They were then washed three times with FACS buffer and divided into three equal aliquots, one of which was used for pAkt and one of which was used for pErk. They were blocked for 20 min at room temperature with 5% normal goat serum and 1:100 human Fc block (Trustain FcX, Biolegend) and stained at room temperature for 1 h with a 1:100 dilution of rabbit anti-pAkt (Cell Signaling Technology, Ser473, clone D9E) or 1:100 rabbit anti-pErk1/2 (Cell Signaling Technology, Thr202/Tyr204, clone 197G2). They were washed twice in FACS buffer, stained for 1 h at room temperature with 1:300 goat anti-rabbit-AF647 and 1:250 anti-GFP (Biolegend, clone FM2-64G), washed with FACS buffer, and analyzed.

RhoA activation assay

P2RY8 KO Ly8 cells were transduced with variants of interest as described in individual variant validation above. 5 days after transduction, cells were washed with FACS buffer, resuspended in FACS buffer, and 3 x 10^5 transduced cells per sample sorted using FACSAriaFusion. Gating was FSC-A x SSC-A, singlets by FSC-A x FSC-W, and GFP+. Cells were sorted into 25% FBS in PBS at

4°C. Cells were pelleted, resuspended in standard Ly8 media, and put back into culture in 24-well plate. Two days later, cells were expanded into 6-well plates. Two days later, overnight serum starvation by replacing with media containing 0.5% fatty-acid free BSA in place of 10% FBS (otherwise unchanged). Cells were collected ~16 h later (same within a given experiment), washed with migration medium (RPMI, 0.5% fatty-acid free BSA, 10 mM HEPES, and 50 IU mL^{-1} penicillin/streptomycin), and 1.25 x 10^6 cells incubated at 37°C for 10 min. Cells were pelleted, resuspended in 100 μL migration medium, incubated at 37°C for 5 min, then diluted to 200 μL with 100 μL of migration medium containing 200 nM GGG (so 100 nM final) and incubated at 37°C for 3 min. After 3 min, cells were placed on ice, pelleted by centrifugation at 4°C, washed with ice-cold PBS, then lysed with 105 μL ice-cold lysis buffer including protease inhibitor cocktail from RhoA G-LISA GTPase Activation Assay Kit (Cytoskeleton), mixed by pipetting. Debris pelleted by centrifugation 10,000 x *g* for 1 min at 4°C. Small aliquot set aside on ice for protein quantification and remainder snap-frozen and placed at −80°C. Protein quantified using Qubit (Thermo Fisher Scientific)

On day ELISA performed, sample thawed on ice, diluted to 2 mg mL^{-1} total protein concentration with lysis buffer based on pre-freeze quantification, and each sample assayed as technical duplicates for active RhoA using RhoA G-LISA GTPase Activation Assay Kit (Cytoskeleton) and for total RhoA using Total RhoA ELISA Kit (Cytoskeleton) per kit protocols. Colorimetric analysis via absorption at 490 nm using plate reader. In each experiment, mean absorption (across duplicates) determined, ratio of active RhoA to total RhoA absorption calculated, with normalization then performed to WT P2RY8 results (1–2 biological replicates) within that experiment.

TRUPATH BRET trimeric G protein assay

This assay was performed in 293T cells. To start, 5 x 10^5 cells were plated per well in a 6-well plate. The next day, they were transfected. Specifically, to 250 μL of Opti-MEM, added 150 ng of P2RY8 WT, P2RY8 variant, or GFP only plasmid (same POP2E original or modified as used in *Individual variant validation* above), 100 ng pcDNA5/FRT/TO-GAlpha13-RLuc8, 100 ng pcDNA3.1-Beta3, 100 ng of pcDNA3.1-GGamma9-GFP2, and 1.35 μL of *Trans*-IT 2020 reagent (Mirus) and gently mixed; after 25 min incubation at room temperature, this was added dropwise to well of the 293T cells. (pcDNA5/FRT/TO-GAlpha13-RLuc8, pcDNA3.1-Beta3 and pcDNA3.1-GGamma9-GFP2 were gifts from Bryan Roth, Addgene plasmids #140986, 140988, and 140991.[42]) The next day, 96-well white flat-bottom plates (Greiner Bio-One) were treated for 2 h with 50 μg mL^{-1} poly-D-lysine (diluted from 1 mg mL^{-1}, EMD-Millipore) in sterile ultra-distilled water, washed once with water, then dried for 1 h. Culture medium removed from the transfected cells, cells were dissociated from plate using PBS with 0.5 mM EDTA, washed, resuspended at 2.67 x 10^5 cells mL^{-1} in culture medium, and 150 μL (4 x 10^4 cells) aliquoted per well of poly-D-lysine treated plate. The following day, the plate was centrifuged at 500 x g for 1 min, culture medium removed, and 60 μL of assay medium (Hanks' balanced salt solution plus 20 mM HEPES) was added to each well. From 2.5 mM stock of coelenterazine 400a (Nanolight), a 50 μM dilution in assay buffer was made. In addition, desired dilutions of GGG in assay were prepared (at four times the ultimately desired concentration). Transported cells on ice to building with BRET-capable plate reader, plate again centrifuged 500 x *g* for 1 min, and 10 μL of coelenterazine working solution was added. For basal activation analysis, after 5 min loaded on plate reader and measured 420 ± 20 nm and 515 ± 20 nm for luciferase and GFP, respectively, and calculated luminescence to GFP ratio and determined mean across all wells of that genotype. For GGG dose response, 10 min after adding coelenterazine working solution, 30 μL of desired GGG solution was added, yielding final concentrations ranging from 100 pM to 10 μM; there was also a 1:100 DMSO (vehicle) condition. After 8 min, loaded on plate reader and measured 420 ± 20 nm and 515 ± 20 nm for luciferase and GFP, respectively. Measured 6 times, each spaced by 3 min. For analysis, calculated luminescence to GFP ratio for each time point and determined mean across the six measurements.

NanoBit β-arrestin assay

This assay was performed in 293T cells. β2AR-LgBiT and β-Arr2-SmBiT plasmids had previously been generated.[54] The β2AR-LgBiT plasmid was digested with EcoRI and BmtI and β2AR replaced with P2RY8 coding sequence, either WT or with V29W, K180I, W181M, or L220Y missense variants, using PCR on the plasmids previously generated for *Individual variant validation*. On day 0, 293T cells were plated into 6-well plate. On Day 1, with cells now ~80% confluent, cells were transfected. Specifically, to 250 μL Opti-MEM added 400 ng of desired LgBiT construct, 100 ng of β-Arr2-SmBiT, and 1.5 μL of *Trans*-IT 2020 (Mirus) and gently mixed. After 25 min incubation at room temperature, this was added dropwise to well of the 293T cells. On Day 2, culture medium was removed. Cells were dissociated from plate using PBS with 0.5 mM EDTA, washed, and counted. Cells were resuspended at 4 x 10^5 cells mL^{-1} in assay solution (Hanks' balanced salt solution with 20 mM HEPES) and 100 μL (4 x 10^4 cells) added to wells in 96-well white flat-bottom plate. To this, added 50 μL of 15 μM coelenterazine h (Nanolight). After 10 min, measured baseline luminescence. Then added 50 μL of desired GGG dilution in assay solution with 5 μM coelenterazine h to yield final concentrations ranging from 100 pM to 10 μM as well as a DMSO (vehicle) condition. After 10 min, measured luminescence again. For analysis, looked at ratio of post-GGG signal to baseline signal, normalized to DMSO condition ratio.

Bone marrow chimera generation and analysis

Mice to be used as bone marrow (BM) donors (C57BL/6J, 8–12 weeks old) were injected intraperitoneally with 3 mg 5-fluorouracil (Sigma-Aldrich). BM was collected after 4 days and cultured in DMEM containing 15% FBS, penicillin (50 IU mL^{-1}), streptomycin (50 μg mL^{-1}), and 10 mM HEPES, supplemented with 250 ng mL^{-1} IL-3, 50 ng mL^{-1} IL-6, and 100 ng mL^{-1} SCF (Peprotech). Cells were transduced with MSCV-EV-GFP, MSCV-P2RY8(WT)-IRES-GFP, MSCV-P2RY8(K180I)-IRES-GFP, or MSCV-P2RY8(W181M)-IRES-GFP) on days 1 and 2 using viral supernatant concentrate thawed, diluted to original concentration with DMEM, 10% FBS,

10 mM HEPES, and 4 μg mL^{-1} polybrene and transduction performed via 2 h centrifugation at 2400 rpm at 32°C, followed by replacement with culture media. Recipient CD45.1 B6 mice, age 7–10 weeks, were lethally irradiated with 900 rads (evenly split dose separated by 3 h) and then injected IV with relevant transduced BM cells. For every 3–3.5 recipient mice, 1 donor mouse was used. Mice were analyzed 10–12 weeks after reconstitution.

For analysis, spleen, mesenteric lymph nodes, and Peyer's patches were isolated. Mesenteric lymph nodes and Peyer's patches were digested for 20 min at 37°C, shaking, in 1 mg mL^{-1} collagenase VIII (Sigma-Aldrich) in RPMI with 10% FBS. After mashing, straining, and washing, tissues were blocked for 10 min with 1:100 Fc block (Bio-X-Cell) in FACS buffer (PBS, 2% FBS, 1 mM EDTA), then stained for 30 min on ice with anti-CD45.2 PE (Biolegend 109808), anti-CD45.1 APC (Cytek 20-0453-U100), anti-GL7 PacBlue (Biolegend 144614), anti-IgD PerCP-Cy5.5 (Biolegend 405710), anti-CD95 PE-Cy7 (Fisher 557653), anti-B220 BV785 (Biolegend 103246), and, for spleen, also anti-CD8a BV510 (Biolegend 100752), anti-CD11b BV570 (Biolegend 101233), anti-CD4 BV711 (Biolegend 100447), and anti-CD3ε AF700 (Biolegend 152316), all 1:200. Cells were washed, stained for 10 min with e780 fixable viability dye (eBiosciences), and washed again. Cells were then analyzed on FACSymphony (BD).

B cell transduction, transfer, and analysis

P2RY8 was previously cloned into the MSCV-GFP retroviral vector as described.[28] The GFP was replaced by digestion of MSCV-P2RY8-IRES-GFP with BstXI and PacI and insertion of TagBFP PCR product using HiFi DNA Assembly Kit (NEB). The WT P2RY8 MSCV was digested with BglII and NotI and K180I or W181M P2RY8 PCR products (from the constructs detailed in *Individual variant validation* above) were inserted using HiFi DNA Assembly Kit (NEB). Retrovirus encoding P2RY8-TagBFP, K180I-GFP, and W181M-GFP were produced using Plat-E packaging line as described.[27] EasySep kits were used to enrich B cells from mouse spleens by removing T cells with biotin-conjugated anti-CD3ε (Biolegend, clone 145-2C11) and streptavidin-conjugated beads (EasySep Streptavidin RapidSpheres). B cells were cultured in complete RPMI (10% FBS, 10 mM HEPES, 1x GlutaMax, 50 IU mL^{-1} penicillin/streptomycin, and 55 μM β-mercaptoethanol) along with 25 μg mL^{-1} (1:4000 dilution) anti-CD180 (BD Biosciences, clone RP/14).

Transduction was performed 24 h after activation. Plates were centrifuged and culture supernatant was saved. Retroviral supernatants were thawed from −80°C and HEPES added to 20 mM and polybrene (EMD Millipore) added to 2 μg mL^{-1}. This was used to spinfect the cells for 2 h at room temperature, centrifuging at 2500 rpm. The viral supernatant was then aspirated and half the original culture medium was replaced along with an equal volume of culture medium with freshly added anti-CD180. The spinfection was repeated a second time 24 h later.

Twenty-four hours after the second transduction, cells were collected from the plate, washed twice, and placed on ice. A small aliquot was analyzed by flow cytometry to determine the proportion of transduced (GFP^+ or $TagBFP^+$) cells. Cells were diluted and pooled (WT P2RY8 and K180I P2RY8 or WT P2RY8 and W181M P2RY8) to desired ratio and injected IV into recipient mice, who had been immunized intraperitoneally 7 days earlier with 2 x 10^8 SRBCs. Mice received 4-5 x 10^6 WT-$TagBFP^+$ cells and 7-10 x 10^6 K180I-GFP^+ or W181M-GFP^+ cells.

Mice were analyzed 24 h after transfer. Spleens were harvested, cut into 4-5 sections, and fixed for 30 min in 4% paraformaldehyde in PBS at 4°C, washed, then dehydrated overnight at 4°C in 30% sucrose solution. Spleen segments were placed in cassette with OCT medium (Sakura), flash frozen, and placed at −80°C for storage. Cryostat was used to cut 7 μm sections. These were dried at room temperature for one hour then rehydrated with PBS 0.1% fatty-acid-free (FAF) BSA for 10 min. They were stained overnight at 4°C with primary stain of PBS 0.1% FAF-BSA, 1:100 normal mouse serum, 1:100 anti-GFP AF488 (rabbit polyclonal, Invitrogen A21311), 1:100 anti-CR1 (CD35)-biotin (BD 553816, clone 8C12), 1:100 anti-IgD AF647 (Biolegend 405708, clone 11-26c2a), and 1:200 or 1:250 anti-TagFP AZ568 (NanoTag N0502-AF568). Cells were washed 3 times with PBS 0.1% FAF-BSA then secondary stain was PBS 0.1% FAF-BSA, 1:100 normal mouse serum, 1:200 streptavidin-AMCA for 1 h at room temperature. Cells were washed 2 times with PBS 0.1% FAF-BSA, once with PBS, then coverslip affixed using Fluoro-Mount, allowed to harden at 4 C for one hour. Images were acquired using Zeiss AxioObserver Z1 inverted microscope.

Initial image processing performed in ZEN (Zeiss): AMCA channel black/white rescaled from 0 to 16,034 to 1,596-16,034 for all images obtained. AF555 channel black/white rescaled from 0 to 16,034 to 1,047-9,052 for images in figures. Further analysis performed using Fiji.[60] Follicles were cropped from larger images and regions of interest containing GCs defined. Using consistent thresholds across images, GFP and BFP signal was made binary and Analyze Particles function (size 10–150 μm^2, circularity 0.3–1) used to count cells within follicles and within subsections of follicles coinciding with GCs. Analysis included at least 45 GCs from each mouse.

Expression and purification of P2RY8-miniGα13 protein

Human P2RY8 (Uniprot: Q86VZ1) was cloned into pCDNA-Zeo-TetO, a custom pcDNA3.1 vector containing a tetracycline-inducible gene expression cassette.[55] The resulting P2RY8 construct comprised an N-terminal influenza haemagglutinin signal sequence followed by an M1-FLAG (DYKDDDD) epitope tag. The P2RY8 construct was furthermore fused at the C-terminus to the miniGα_{13} protein via a linker sequence containing a human rhinovirus 3C (HRV 3C) protease cleavage site. The resulting P2RY8–miniGα_{13} construct was transiently transfected into inducible Expi293F TetR cells (unauthenticated and untested for mycoplasma contamination; Thermo Fisher Scientific) using the ExpiFectamine 293 Transfection Kit (Thermo Fisher Scientific) following the manufacturer's instructions. After 16 h, protein expression was induced with 1 μg mL^{-1} doxycycline hyclate (Sigma-Aldrich), and the culture was

placed in a shaking incubator maintained at 37°C and 8% CO_2. After 36 h, cells were harvested by centrifugation at 4,000 x g and stored at −80°C.

For receptor purification, cells were thawed and resuspended in lysis buffer comprising 20 mM HEPES pH 7.50, 10 μM S-geranylgeranyl-L-glutathione (GGG, Cayman Chemical), 100 μM tris(2-carboxyethyl)phosphine (TCEP; Fischer Scientific), and a Pierce protease inhibitor tablet (Thermo Fisher Scientific). Cells were lysed by stirring for 15 min at 4°C and harvested by centrifugation at 16,000 x g for 15 min. To increase the fraction of GGG-bound P2RY8, we also reconstituted GGG into detergent to overcome its low solubility. 25 mg of GGG and 1.5 g of lauryl maltose neopentyl glycol (L-MNG; Anatrace) was dissolved in methanol and allowed to mix for 10 min at RT. The LMNG-GGG mixture was was placed under an Argon stream evaporated to dryness followed by vaccuum dessication overnight. The dry LMNG-GGG mixture was resuspended in 20 mM HEPES pH 7.50, 100 mM NaCl by sonication. Cell pellet was dounce-homogenized in ice-cold solubilization buffer comprising 50 mM HEPES, pH 7.5, 150 mM NaCl, 1% (w/v) lauryl maltose neopentyl glycol (L-MNG; Anatrace) containing 3 mol % GGG, 0.1% (w/v) cholesteryl hemisuccinate (CHS, Steraloids), 5 mM adenosine 5′-triphosphate (ATP; Fisher Scientific), 2 mM $MgCl_2$, 100 μM TCEP, 10 μM GGG, and a Pierce protease inhibitor tablet (Thermo Scientific). The sample was stirred for 1 h at 4°C, and the detergent-solubilized fraction was clarified by centrifugation at 20,000 x g for 30 min. The detergent-solubilized sample was supplemented with 4 mM $CaCl_2$ and incubated in batch with homemade M1-FLAG-antibody conjugated CNBr-Sepharose under slow rotation for 1 h at 4°C. The P2RY8-bound resin was transferred to a glass column and washed with 20 mL of ice-cold buffer comprising 50 mM HEPES, pH 7.5, 150 mM NaCl, 0.1% (w/v) L-MNG containing 3 mol % GGG, 0.01% (w/v) CHS, 5 mM ATP, 2 mM $CaCl_2$, 2 mM $MgCl_2$, 100 μM TCEP. This was followed by 10 column volumes of ice-cold 50 mM HEPES, pH 7.5, 150 mM NaCl, 0.0075% (w/v) L-MNG containing 3 mol % GGG, 0.0025% (w/v) glyco-diosgenin (GDN; Anatrace), 0.001% (w/v) CHS, 2 mM CaCl2, 100 μM TCEP, and 10 μM GGG. Receptor-containing fractions were eluted with ice-cold 20 mM HEPES, pH 7.5, 150 mM NaCl, 0.0075% (w/v) L-MNG containing 3 mol % GGG, 0.0025% (w/v) GDN, 0.001% (w/v) CHS, 5 mM EDTA, 0.2 mg mL^{-1} FLAG peptide, 100 μM TCEP, and 10 μM GGG. Fractions containing P2RY8–miniGα_{13} were concentrated in a 50-kDa MWCO spin filter (Amicon) and purified over a Superdex 200 Increase 10/300 GL size-exclusion chromatography (SEC) column (Cytiva), which was equilibrated with 20 mM HEPES, pH 7.5, 150 mM NaCl, 0.0075% (w/v) L-MNG containing 3 mol % GGG, 0.0025% (w/v) GDN, 0.001% (w/v) CHS, 100 μM TCEP, and 10 μM GGG. Fractions containing monodisperse P2RY8–miniGα_{13} were combined and concentrated in a 50-kDa MWCO spin filter before complexing with G$\beta_1\gamma_2$.

Expression and purification of Gβ1γ2

Purified G$\beta_1\gamma_2$ was generated as described previously.[52] Briefly, a baculovirus was generated in *Spodoptera frugiperda* Sf9 insect cells (unauthenticated and untested for mycoplasma contamination, Expression Systems) using the pVLDual expression vector encoding both human Gβ $_1$ subunit with an HRV 3C cleavable N-terminal 6xHis-tag and the untagged human Gγ_2 subunit. G$\beta_1\gamma_2$ was expressed in *Trichoplusia ni* Hi5 insect cells (unauthenticated and untested for mycoplasma contamination, Expression Systems) by infection with G$\beta_1\gamma_2$-baculovirus at a density of 3.0×10^6 cells ml^{-1} and grown for 48 h at 27°C with 130 r.p.m. shaking. Harvested cells were resuspended in lysis buffer comprised of 20 mM HEPES, pH 8.0, 5 mM β-mercaptoethanol (β-ME), 20 μg mL^{-1} leupeptin, and 160 μg mL^{-1} benzamidine. Lysed cells were pelleted at 20,000 x g for 15 min, and solubilized with 20 mM HEPES, pH 8, 100 mM sodium chloride, 1% (w/v) sodium cholate (Sigma-Aldrich), 0.05% (w/v) n-dodecyl-β-*d*-maltopyranoside (DM; Anatrace) and 5 mM β-ME. Detergent-solubilized G$\beta_1\gamma_2$ was clarified by centrifugation at 20,000 x g for 30 min and was then incubated with HisPur Ni-NTA resin (Thermo Fisher Scientific) under slow rotation for 1.5 h at 4°C. G$\beta_1\gamma_2$-bound resin was transferred to a glass column and washed extensively. Detergent was exchanged on-column to 0.1% (w/v) L-MNG and 0.01% (w/v) CHS, and G$\beta_1\gamma_2$ was eluted with 20 mM HEPES pH 7.50, 100 mM NaCl, 0.1% (w/v) L-MNG, 0.01% (w/v) CHS, 300 mM imidazole, 1 mM DL-dithiothreitol (DTT), 20 μg mL^{-1} leupeptin and 160 μg mL^{-1} benzamidine. G$\beta_1\gamma_2$-containing fractions were pooled and supplemented with homemade 3C protease before overnight dialysis in buffer comprised of 20 mM HEPES, pH 7.50, 100 mM NaCl, 0.02% (w/v) L-MNG, 0.002% (w/v) CHS, 1 mM DTT and 10 mM imidazole. Cleaved G$\beta_1\gamma_2$ was isolated by reverse Ni-NTA, and dephosphorylated by treatment with lambda phosphatase (NEB), calf intestinal phosphatase (NEB) and antarctic phosphatase (NEB) for 1 h at 4°C. The geranylgeranylated G$\beta_1\gamma_2$ heterodimer was isolated by anion exchange chromatography using a Mono Q 4.6/100 PE (Cytiva) column, before overnight dialysis in 20 mM HEPES, pH 7.5, 100 mM NaCl, 0.02% (w/v) L-MNG and 100 μM TCEP. The final sample was concentrated on a 3-kDa MWCO spin filter (Amicon), and 20% (v/v) glycerol was added before flash freezing in liquid N_2 for storage at −80°C.

Preparation of the active-state of P2RY8-G13 complex

To prepare the P2RY8–G$_{13}$ complex, a 2-fold molar excess of purified G$\beta_1\gamma_2$ was added to SEC-purified P2RY8–miniGα_{13} followed by overnight incubation on ice. The sample was purified on a Superdex 200 Increase 10/300 GL SEC column, equilibrated with 20 mM HEPES, pH 7.5, 150 mM NaCl, 0.0075% (w/v) L-MNG containing 3 mol % GGG, 0.0025% (w/v) GDN, 0.001% (w/v) CHS and, 30 μM GGG. Fractions containing the monomeric P2RY8–G$_{13}$ heterotrimeric complex were concentrated on a 50-kDa MWCO spin filter (Amicon) immediately before cryo-EM grid preparation.

Cryo-EM vitrification, data collection, and processing

2.75 μL of the purified P2RY8–G$_{13}$ complex was applied at 1.4 mg mL^{-1} to glow-discharged 300 mesh R1.2/1.3 UltrAuFoil Holey gold grids (Quantifoil). Grids were plunge-frozen in liquid ethane using a Vitrobot Mark IV (Thermo Fisher) with a 10-s hold period, blot force

of 0, and blotting time varying between 1.5 and 3.0 s while maintaining 100% humidity and 4°C. Vitrified grids were clipped with Autogrid sample carrier assemblies (Thermo Fisher Scientific) immediately before imaging. Movies of P2RY8–G_{13} embedded in ice were recorded on a 300 kV FEI Titan Krios microscope equipped with a Falcon 4i camera (Thermo Fisher Scientific, located at the HHMI Janelia Research Campus). A nominal magnification of ×130,000 was used in resolution mode with a physical pixel size of 0.94 Å per pixel, and movies were recorded with a total exposure of 50 e− $Å^{-2}$. Movies (n = 12,053) were imported into cryoSPARC (Structura Biotechnology) with 80 EER fractions, motion-corrected micrographs were generated with the patch motion correct tool beofre calculation of patch contrast transfer functions (patch CTFs). A threshold of CTF fit resolution of more than 6 Å was used to exclude low-quality micrographs. Particles were template picked using a 20 Å low-pass-filtered model that was generated ab initio from data collected during earlier screening sessions on a 200-kV Glacios microscope located at UCSF. Particles (n = 10,117,438) were extracted with a box size of 288 pixels binned to 72 pixels and sorted by two rounds of 2D classification. The resulting 2,352,975 particles were sorted by ab initio 3D reconstruction with two classes, re-extracted with a box size of 288 pixels binned to 144 pixels, and sorted by additional two rounds of ab initio 3D construction. Particles (n = 799,516) were extracted with an unbinned box size of 288 pixels and were subjected to non-uniform refinement followed by local refinement using a mask covering only the 7TM domain of P2RY8. Particles were further sorted by two rounds of 3D classification using 4 classes and a filter resolution of 3.00 Å and 2.75 Å respectively. The resulting 90,243 particles were subjected to non-uniform refinement followed by local refinement using an inclusion mask covering the 7TM domain, using poses/shift Gaussian priors with standard deviation of rotational and shift magnitudes limited to 1° and 1 Å, respectively.

Model building and refinement

Model building and refinement were carried out using a starting model based on the AlphaFold2 predicted structure of P2RY8 (Uniprot: Q86VZ1), the deposited structure of $G\alpha_{13}$ (PDB code: 7T6B),[51] and the deposited structure of $G\beta_1\gamma_2$ (PDB code: 8F76)[52] which were fitted into the P2RY8–G_{13} map using UCSF ChimeraX.[61,62] A draft model was generated using ISOLDE[63] and was further refined by iterations of real-space refinement in Phenix v1.20[64] and manual refinement in Coot v0.8.9.2.[66] The GGG model and rotamer library were generated with the eLBOW extension[65] in Phenix and docked using Coot. The resulting model was extensively refined in Phenix and map-model validations were carried out using Molprobity v4.5[67] and EMRinger.[68] Coordinates for the P2RY8-$G\alpha_{13}$ complex were deposited in the RCSB Protein DataBank, PDB: 9ECJ. EM density map for the P2RY8-$G\alpha_{13}$ complex was deposited in the Electron Microscopy DataBank under accession codes EMD-47912 (full map) and EMD-47914 (7TM map).

Chemical synthesis

GGG used in WEHI migration assay was synthesized in house as previously described.[28] GGG for all other assays was purchased from Cayman Chemical and resuspended in DMSO for 1 mM stock solution. Leukotriene C_4 was also purchased from Cayman Chemical.

Synthesis of other glutathione conjugates followed similar approach to GGG.[28] Compound 1 refers to S-octane-L-glutathione, compound 2 refers to S-hexadecane-L-glutathione, compound 3 refers to S-isoprenyl-L-glutathione, and compound 4 refers to S-farnesyl-L-glutathione. Unless otherwise stated, all reagents were purchased from MilliporeSigma. Briefly, 20 mg (1 eq.) of farnesol (for compound 4) was stirred in 1 mL of dry DCM under an atmosphere of nitrogen at room temperature. Triphenylphospine (1.3 eq.) was added, followed by carbon tetrabromide (1.3 eq.), and the reaction was stirred for a further 2–4 h at room temperature. After concentrating the crude reaction under reduced pressure, a small volume of n-hexane was added and the resulting precipitate removed by filtration. Concentration, precipitation and filtration was repeated once again and the concentrated filtrate used in the next step without further purification.

Glutathione (1.1 eq) was dissolved in the minimal volume of 2 M NaOH, and ethanol added until the solution started to become cloudy. Either the compound generated in the first step (for compound 4), or 1-bromooctane, 1-bromohexadecane, or 3,3-Dimethylallyl bromide (for compounds 1, 2, and 3) (1 eq.) was added dropwise and the reaction stirred at room temperature overnight. The pH of the reaction mixture was reduced to 2 by addition of 1 M HCl, and the reaction cooled in an ice bath until a precipitate formed. This precipitate was collected by filtration, washed with a small volume of ice-cold ethanol and then ice-cold water, and dried to yield the final glutathione conjugate.

Previously available data

Germline variants from gnomAD[2] v4.0.0 at https://gnomad.broadinstitute.org. Non-hematologic cancer variants and some DLBCL/Burkitt lymphoma variants from COSMIC[45] at https://cancer.sanger.ac.uk/cosmic, accessed Feb. 1, 2024. GPR68 DMS results[23] at MAVEdb: 00001207.

QUANTIFICATION AND STATISTICAL ANALYSIS

Statistical analysis and plot creation using either R (version 4.3.0 in RStudio; package tidyverse 2.0.0) or Prism (GraphPad). Method of generation for specific figure panel may be found at https://doi.org/10.5281/zenodo.15811041 (subsection 7_DMS_Analysis&Figures.R). Details of statistical analyses are in the figure legends or methods description for each experiment.

Cell Genomics, Volume 5

Supplemental information

Phenotypic pleiotropy of missense variants

in human B cell confinement receptor P2RY8

Taylor N. LaFlam, Christian B. Billesbølle, Tuan Dinh, Finn D. Wolfreys, Erick Lu, Tomas Matteson, Jinping An, Ying Xu, Arushi Singhal, Nadav Brandes, Vasilis Ntranos, Aashish Manglik, Jason G. Cyster, and Chun Jimmie Ye

Figure S1

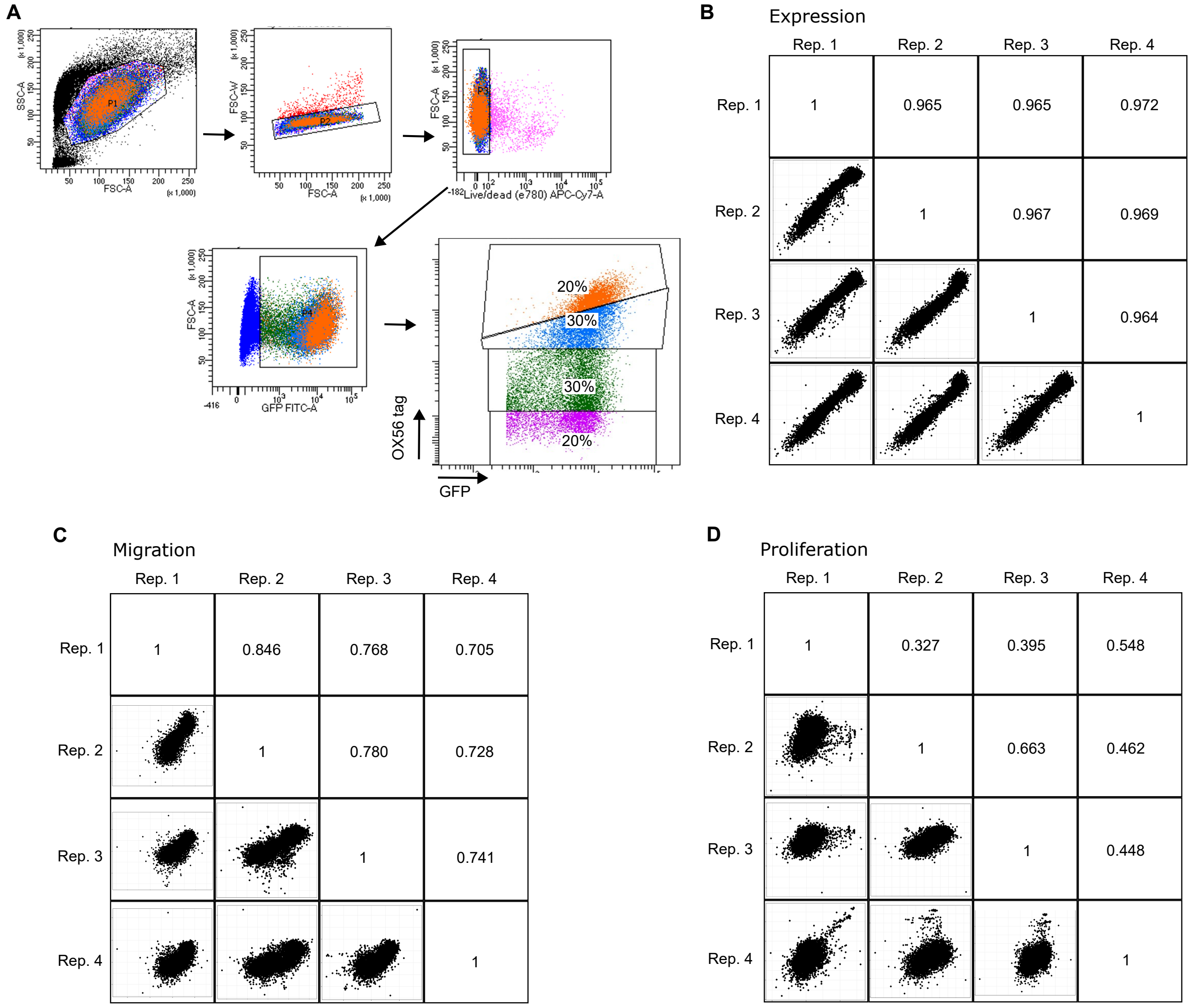

Figure S1: Comparison of DMS replicates, related to Figure 1. (A) Representative flow-assisted cell sorting gating used for surface expression portion of screen. **(B-D)** Dot plots and Pearson correlations of Enrich2 variant scores for each combination of the four replicates for **(B)** surface expression, **(C)** migration, and **(D)** proliferation.

Figure S2

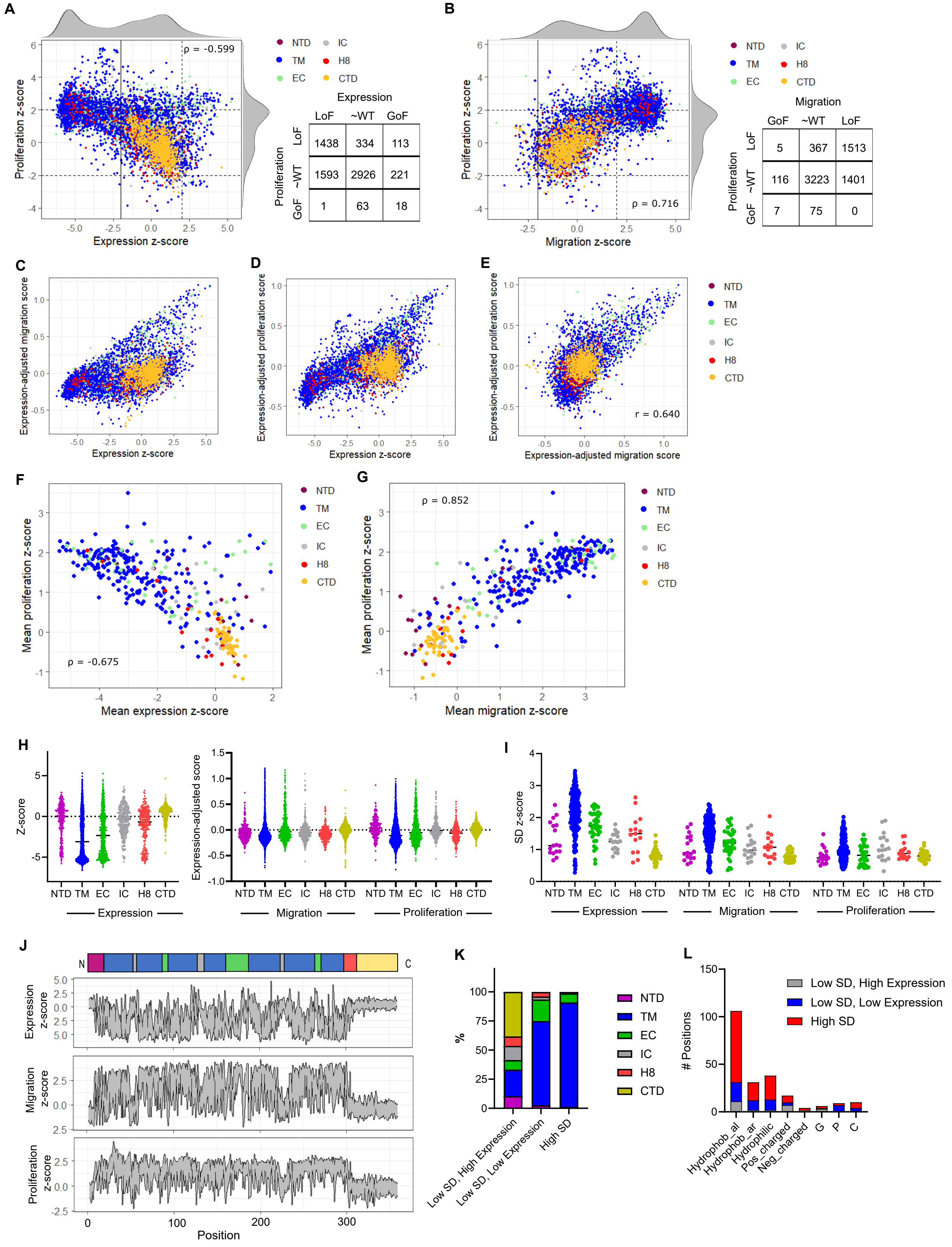
A
ρ = -0.599
Proliferation z-score
Expression z-score
NTD
TM
EC
IC
H8
CTD
Expression
LoF ~WT GoF
Proliferation
LoF 1438 334 113
~WT 1593 2926 221
GoF 1 63 18
B
ρ = 0.716
Proliferation z-score
Migration z-score
Migration
GoF ~WT LoF
Proliferation
LoF 5 367 1513
~WT 116 3223 1401
GoF 7 75 0
C
Expression-adjusted migration score
Expression z-score
D
Expression-adjusted proliferation score
Expression z-score
E
Expression-adjusted proliferation score
Expression-adjusted migration score
r = 0.640
F
Mean proliferation z-score
Mean expression z-score
ρ = -0.675
G
ρ = 0.852
Mean proliferation z-score
Mean migration z-score
H
Z-score
Expression-adjusted score
NTD TM EC IC H8 CTD
Expression
Migration
Proliferation
I
SD z-score
Expression
Migration
Proliferation
J
N
C
Expression z-score
Migration z-score
Proliferation z-score
Position
K
%
Low SD, High Expression
Low SD, Low Expression
High SD
L
Positions
Low SD, High Expression
Low SD, Low Expression
High SD
Hydrophob_al
Hydrophob_ar
Hydrophilic
Pos_charged
Neg_charged
G
P
C

Figure S2: Heterogeneity of P2RY8 DMS results, related to Figure 1. (A) Plot comparing expression and proliferation z-scores of missense variants, colored by protein domain, with table showing variant count by section, boundaries at z-scores of 2 and -2. **(B)** Plot comparing migration and proliferation z-scores of missense variants, colored by protein domain, with table showing variant count by section, boundaries at z-scores of 2 and -2. **(C)** Plot comparing expression z-scores and expression-adjusted migration scores of missense variants, colored by protein domain**. (D)** Plot comparing expression z-scores and expression-adjusted proliferation scores of missense variants, colored by protein domain**. (E)** Plot comparing expression-adjusted migration and expression-adjusted proliferation scores of missense variants, colored by protein domain**. (F)** Plot comparing mean expression and mean proliferation z-scores for each position (averaged across all missense variants at given position), colored by protein domain. **(G)** Plot comparing mean migration z-scores and mean proliferation z-scores for position (averaged across all missense variants at given position), colored by protein domain. **(H)** Variant expression z-scores, expression-adjusted migration scores, and expression-adjusted proliferation scores, partitioned by protein domain; lines mark medians; negative scores are LoF for expression, GoF for migration and proliferation. **(I)** Standard deviation of variant expression, migration, and proliferation z-scores within each position, partitioned by protein domain; lines mark medians. **(J)** Line plots depicting maximum and minimum variant z-scores at each position for expression, migration, and proliferation. **(K)** Distribution across protein domains of positions partitioned by missense variant expression mean and SD; threshold between low and high SD being SD of 2; threshold between low and high expression being mean expression z-score of -2. **(L)** Graph of number of TM positions with each expression score pattern, partitioned by WT amino acid; threshold between low and high SD being SD of 2; threshold between low and high expression being mean expression z-score of -2. Hydrophob_al = A, I, L, M, or V; Hydrophob_ar = F, W, or Y; Hydrophilic = N, S, T, or Y; Pos-charged = H, K, or R; Neg_charged = D or E; CTD, C-terminal domain; EC, extracellular loop; GoF, gain-of-function; H8, helix 8; IC, intracellular loop; LoF, loss-of-function; N, N-terminus; NTD, N-terminal domain; r, Pearson correlation coefficient; TM, transmembrane helices; ρ, Spearman correlation coefficient.

Figure S3

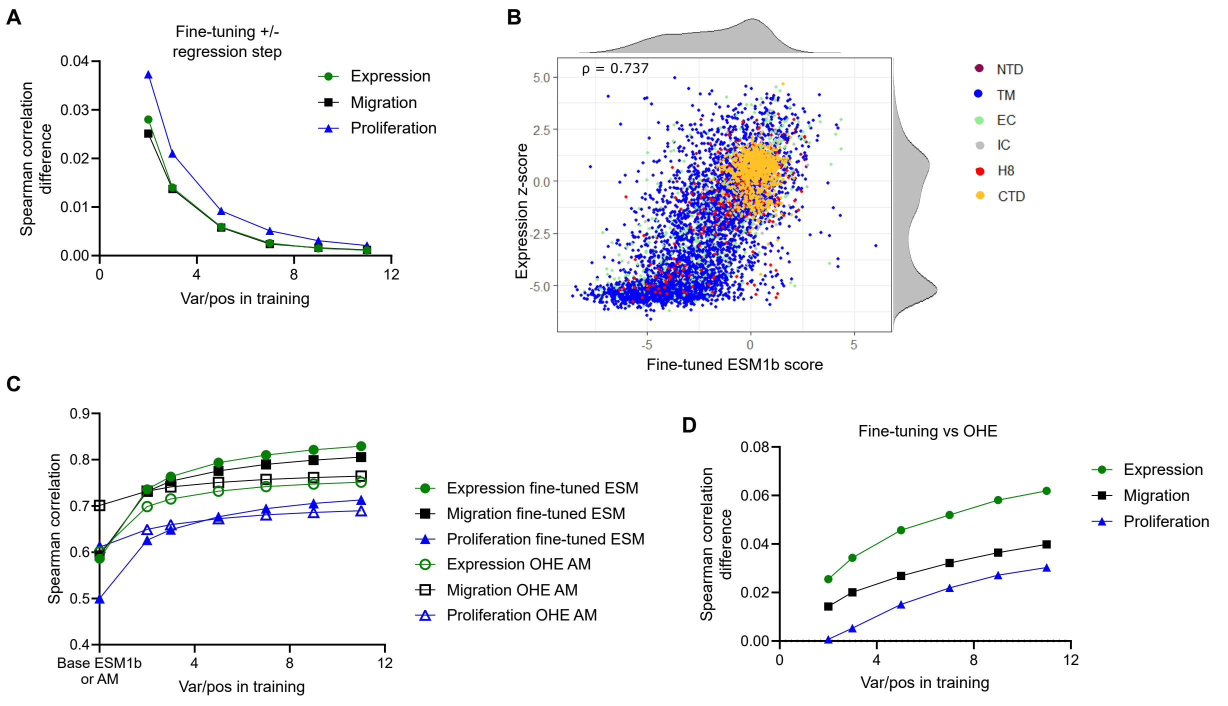

Figure S3: Supplemental ESM1b fine-tuning analyses, related to Figure 2. (A) Plot comparing mean Spearman correlations between DMS effect sizes and fine-tuned ESM1b with various training set sizes, with or without final ridge regression step (value > 0 indicates higher correlation with ridge regression). **(B)** Plot comparing representative 2 variant/position fine-tuned ESM1b and DMS expression z-score, variants colored by protein domain. **(C)** Plot comparing Spearman correlation between DMS z-scores and fine-tuned ESM1b or OHE AM scores as training set size varies. Shows mean and SD (k = 50). **(D)** Plot comparing difference in mean Spearman correlation at given training set size between DMS z-scores and fine-tuned ESM1b or OHE ESM1b (values > 0 indicate higher correlation with fine-tuning). CTD, C-terminal domain; EC, extracellular loop; GoF, gain-of-function; H8, helix 8; IC, intracellular loop; LoF, loss-of-function; NTD, N-terminal domain; OHE, one-hot encoded regression; TM, transmembrane helices; ρ, Spearman correlation coefficient.

Figure S4

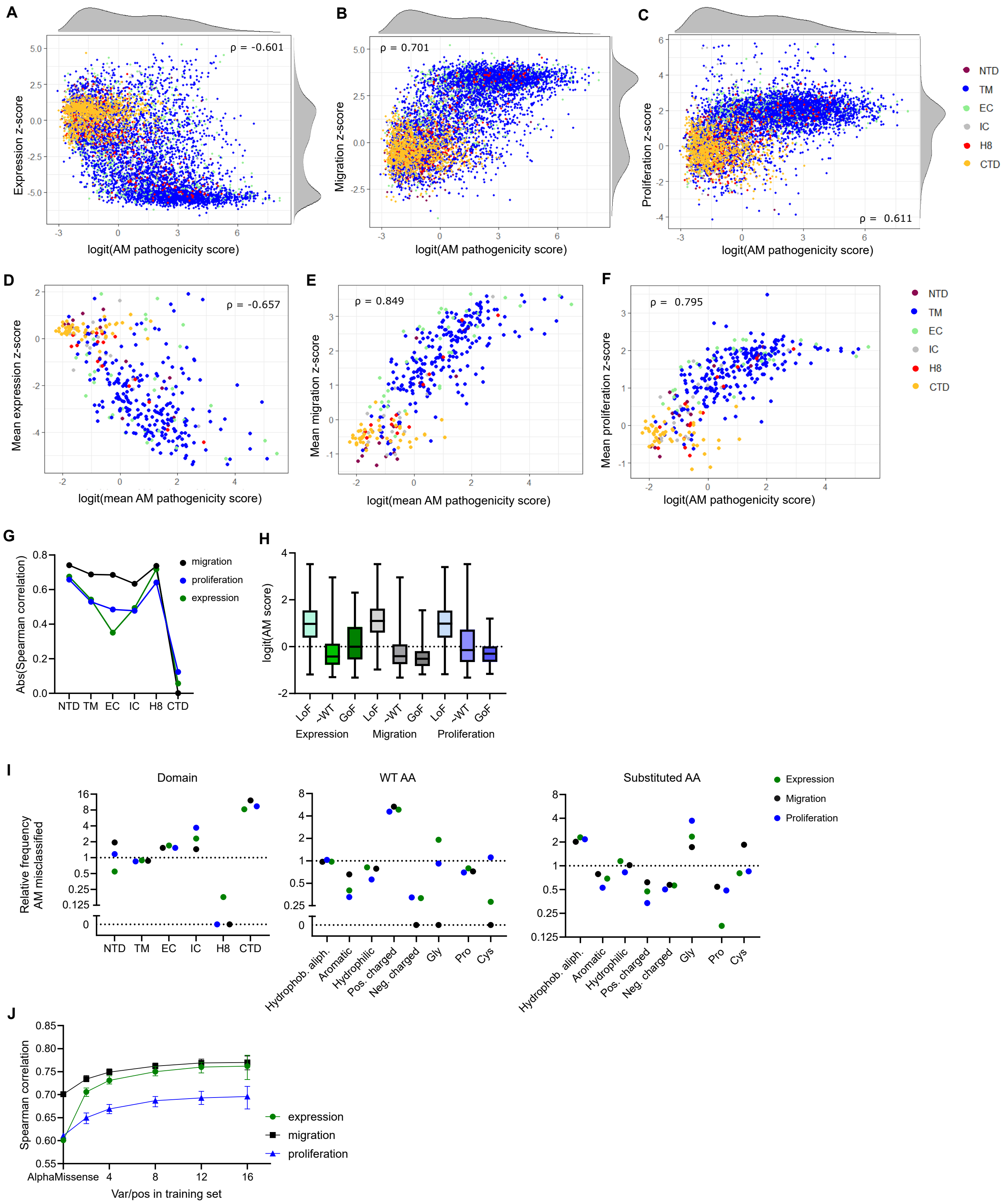

A
B
C
D
E
F
G
H
I
J
ρ = -0.601
ρ = 0.701
ρ = 0.611
ρ = -0.657
ρ = 0.849
ρ = 0.795
Expression z-score
Migration z-score
Proliferation z-score
logit(AM pathogenicity score)
Mean expression z-score
Mean migration z-score
Mean proliferation z-score
logit(mean AM pathogenicity score)
NTD
TM
EC
IC
H8
CTD
Abs(Spearman correlation)
migration
proliferation
expression
logit(AM score)
LoF
~WT
GoF
Expression
Migration
Proliferation
Domain
WT AA
Substituted AA
Relative frequency AM misclassified
Hydrophob. aliph.
Aromatic
Hydrophilic
Pos. charged
Neg. charged
Gly
Pro
Cys
Spearman correlation
AlphaMissense
Var/pos in training set

Figure S4: Improving AlphaMissense variant effect prediction using limited experimental data, related to Figure 2. (A-C) Plots comparing AM pathogenicity score and **(A)** expression z-score, **(B)** migration z-score, or **(C)** proliferation z-score for each variant, colored by protein domain. **(D-F)** Plots comparing mean AM pathogenicity score and **(D)** expression mean z-score, **(E)** migration mean z-score, or **(F)** proliferation mean z-score for each position, colored by protein domain. **(G)** Plot of Spearman correlation between logit-transformed AM pathogenicity score and variant expression, migration, and proliferation z-scores, variants partitioned by protein domain. **(H)** Plot comparing distribution of AM pathogenicity scores for each variant, partitioned by DMS phenotypes. Whiskers extend to maximum and minimum, boxes shows 25th percentile, median, and 75th percentile. **(I)** Plots showing proportion of deleterious variants (z-score < -2 for expression, > 2 for migration and proliferation) with benign AM pathogenicity scores (< 0.34) relative to proportion of deleterious variants with non-benign AM scores, as partitioned by protein domain (left), WT amino acid class (center), and substituted (variant) amino acid class (right). Hydrophobic aliphatic residues are A, I, L, M, and V; aromatic residues are F, W, and Y; hydrophilic residues are N, Q, S, and T; positively charged residues are H, K, and R; negatively charged residues are D and E. (**J)** Plot of Spearman correlation between OHE AM scores and DMS z-scores as training set size varies. Shows mean and SD (k = 50). AM, AlphaMissense; CTD, C-terminal domain; EC, extracellular loop; GoF, GoF, gain-of-function, z-score > 2 for expression, < -2 for migration, proliferation; LoF, loss-of-function, z-score < -2 for expression, > 2 for migration, proliferation; H8, helix 8; IC, intracellular loop; NTD, N-terminal domain; OHE, one-hot encoded regression, ρ, Spearman correlation coefficient.

Figure S5

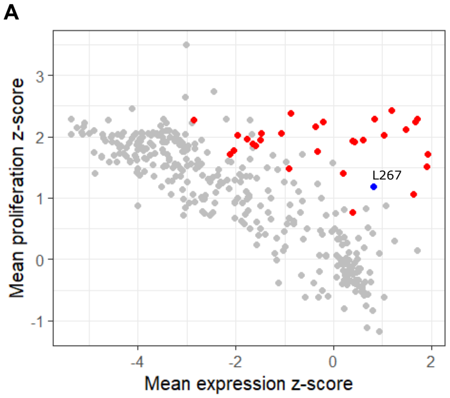

Figure S5: Proliferation phenotype of high expression-adjusted migration score positions, related to Figure 3. (A) Plot comparing expression and proliferation mean z-scores for each position, with those with high expression-adjusted migration scores (Fig. 3a) colored red; additional position with high expression-adjusted proliferation but not also expression-adjusted migration score colored blue.

Figure S6

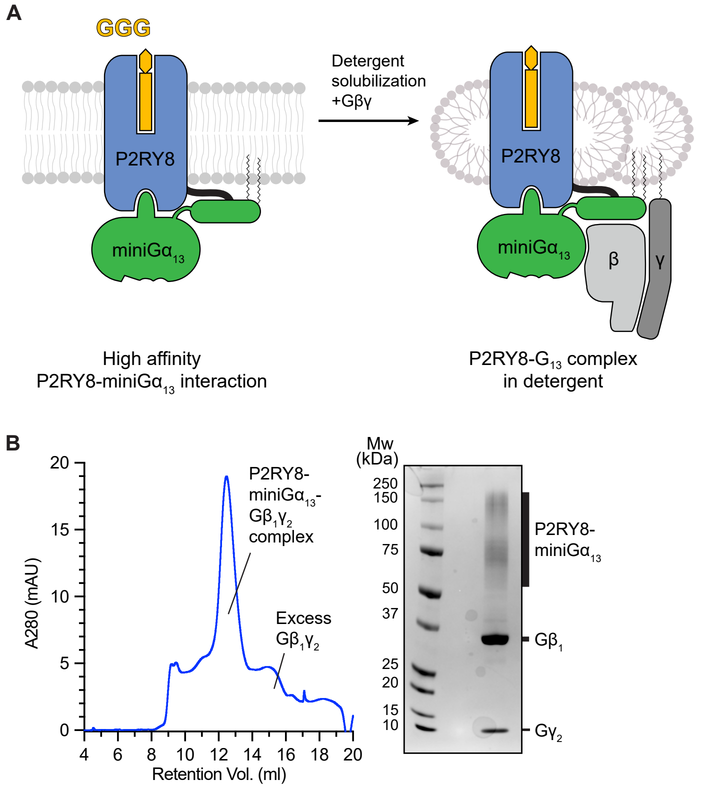

Figure S6: Biochemical preparation of P2RY8-G13 complex bound to GGG, related to Figure 4. (A) Schematic outlining strategy for stabilization and purification of P2RY8 bound to G_{13} and GGG. This diagram is modified from one previously published [S1]. **(B)** Size-exclusion chromatogram of purified P2RY8-G_{13} complex used for structure determination together with representative SDS-PAGE gel analysis of the collected fraction containing the P2RY8-G_{13} complex.

Figure S7

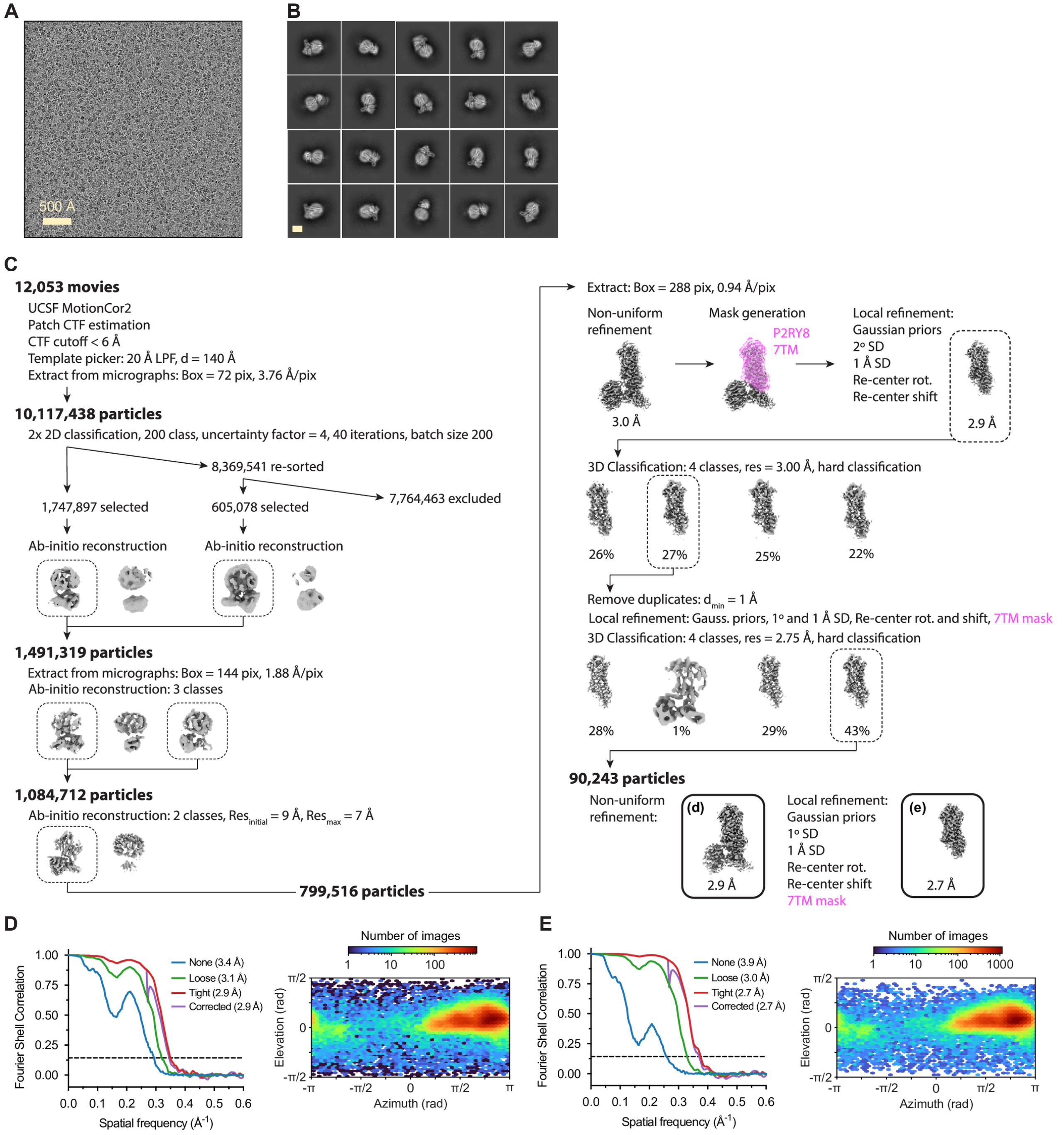

Figure S7: Cryo-EM data processing for P2RY8-G13, related to Figure 4. (A) Representative cryo-electron micrograph from the curated P2RY8-G13 data set (n = 12,053 obtained from a Titan Krios microscope). **(B)** A subset of highly populated, reference-free 2D-class averages are shown. Scale bar is 50 Å. **(C)** Schematic showing the image processing workflow for P2RY8-G13. Initial processing performed using UCSF MotionCor2 and cryoSPARC, where particles were sorted using a combination of 2D classification, ab-inition reconstruction, and 3D classification. Finally, EM maps were obtained in cryoSPARC using the non-uniform and local refinement tools. Dashed boxes indicated selected classes, and 3D volumes of classes and refinements are shown along with the global gold-standard Fourier shell correlation (GSFSC) resolutions. **(D,E)** Map validation for the P2RY8-G13, **(D)** globally refined, and **(E)** locally refined cryo-EM maps. GSFSC curves are calculated in cryoSPARC. Euler angle distributions calculated in cryoSPARC are also provided for each map.

Figure S8

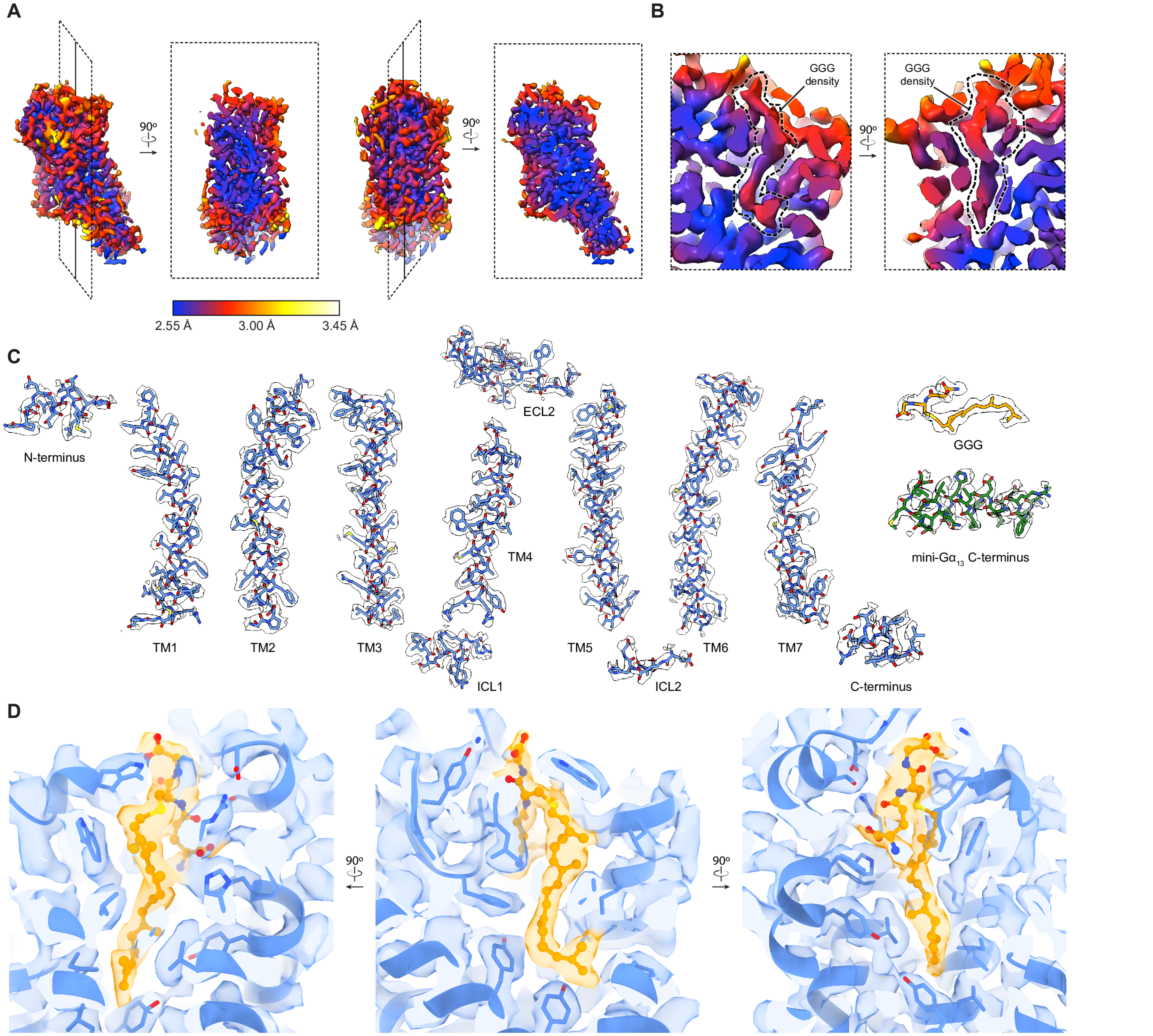

Figure S8: Cryo-EM density and atomic model, related to Figures 4 and 5. (A) Orthogonal views of local resolution for the locally refined map covering the 7TM domain of the P2RY8-G13 complex, calculated with the local resolution estimation tool in cryoSPARC. **(B)** Close-up view showing the local resolution of the GGG binding site. **(C)** Representative cryo-EM densities from the 3D reconstruction of P2RY8 from a sharpened, locally refined map of P2RY8-G13 at a map threshold of 1.02. Shown are the transmembrane helices and loop regions of P2RY8, the C-terminal helix of mini-Gα13, as well as GGG. **(D)** Close-up views of cryo-EM density supporting GGG binding pose (orange sticks and density) using a sharpened, locally refined map of P2RY8-G13 at a map threshold of 1.02.

Figure S9

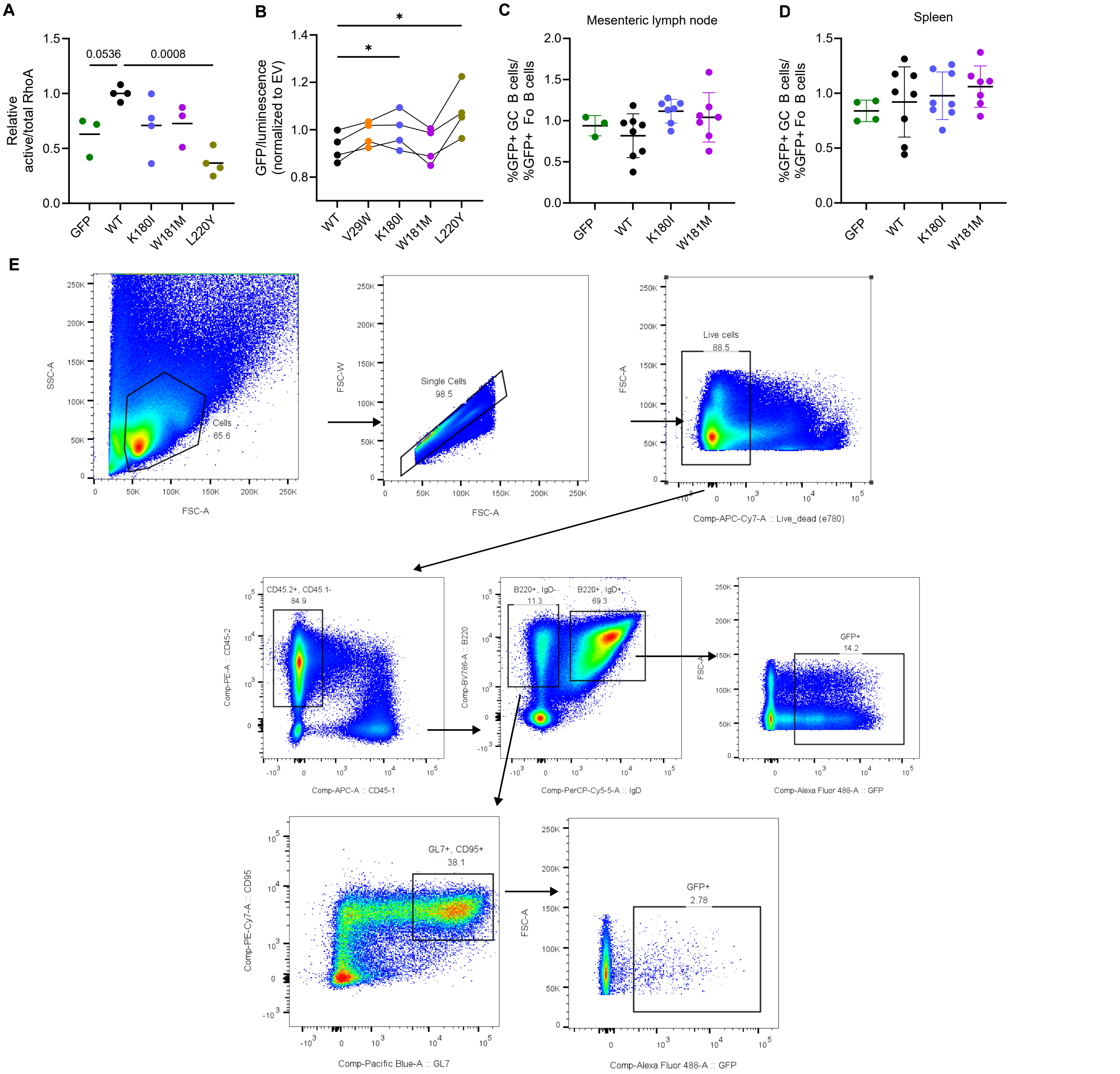

Figure S9: In vivo variant analysis in spleen and mesenteric lymph node, related to Figure 6. (A) Transduced Ly8 cells treated with GGG, lysed, and aliquots assayed for total RhoA and active (GTP-bound) RhoA by ELISA, plot depicts ratio of active to total RhoA signal, normalized within each experiment to WT P2RY8. Pooled results from 3 experiments, each point is a biological replicate. One-way ANOVA with Dunnett's multiple comparison's test with comparisons to WT. **(B)** BRET assay: 293T cells, co-transfected with P2RY8 variant, RLuc-Gα13 fusion, Gβ, and Gγ-GFP fusion, baseline ratio of luminescence and GFP fluorescence determined, ratio normalized to GFP only (EV). Each point is a biological replicate. Results from 4 experiments with line connecting variant results from same experiment. RM one-way ANOVA withnGeisser-Greenhouse correction, comparison to WT with Dunnett's multiple comparisons adjustment. * adjusted p-value of 0.01-0.05. **(C,D)** Irradiated CD45.1 mice were reconstituted with bone marrow transduced with EV-GFP, WT-P2RY8-GFP, K180I-GFP, or W181M-GFP. After reconstitution, mesenteric lymph nodes **(C)** and spleen **(D)** were analyzed for the frequency of GFP+ cells among GC and follicular B cells and the ratio plotted. Pooled from 3 experiments, each point is a mouse. Graphs show means and SDs. **(E)** Representative flow cytometry gating; this depicts this depicts a Peyer's patch sample. GC B cells identified as singlet live cells, B220+, IgD-, GL7+, CD95+; follicular B cells identified as singlet live cells, B220+, IgD+.

Figure S10

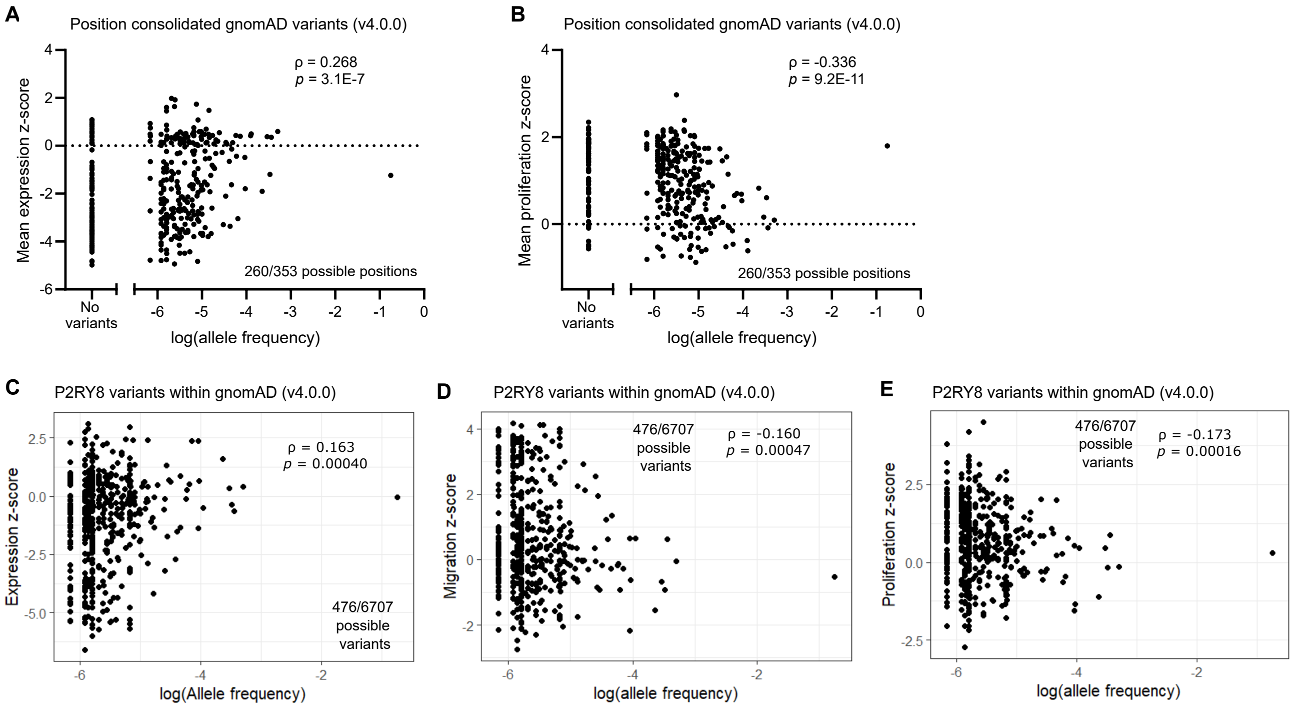

F

Expression

	gnomAD	DLBCL/BL	Non-hem COSMIC
All var	D=0.2039 p=2.22E-16	D=0.3223 p=1.51E-4	D=0.2222 p=5.81E-7
Non-hem COSMIC	D=0.153 p=0.0081	D=0.534 p=6.69E-10	
DLBCL/BL	D=0.5205 p=2.76E-10		

Migration

	gnomAD	DLBCL/BL	Non-hem COSMIC
All var	D=0.2027 p=4.44E-16	D=0.3795 p=3.87E-6	D=0.2316 p=1.59E-7
Non-hem COSMIC	D=0.1963 p=2.39E-4	D=0.5839 p=7.31E-12	
DLBCL/BL	D=0.5724 p=2.37E-12		

Proliferation

	gnomAD	DLBCL/BL	Non-hem COSMIC
All var	D=0.2141 p=2.2E-16	D=0.2971 p=6.29E-4	D=0.1688 p=3.36E-4
Non-hem COSMIC	D=0.0642 p=0.719	D=0.4596 p=2.42E-7	
DLBCL/BL	D=0.5023 p=1.31E-9		

Figure S10: Additional germline and lymphoma-associated P2RY8 variant analysis, related to Figure 7. **(A,B)** Plot comparing summed allele frequency for all missense variants within each position in gnomAD (v4.0.0) to **(A)** mean expression z-score or **(B)** mean proliferation z-score for each position. **(C-E),** Plot comparing missense variant allele frequency in gnomAD (v4.0.0) to the **(C)** expression, **(D)** migration, or **(E)** proliferation z-scores for those variants. **(F)** Tables of Kolmogorov-Smirnov test results comparing expression, migration, and proliferation score distributions for all missense variants, variants present in gnomAD (v4.0.0), non-hematologic cancer variants from COSMIC, and set of DLBCL and Burkitt lymphoma variants. Asymptotic two-sample Kolmogorov-Smirnov test performed; p-values not adjusted for multiple comparison. **(A-E)** ρ, Spearman correlation; p-value calculated using algorithm AS 89 with Edgeworth series approximation.

Table S3. Cryo-EM data collection, refinement, and validation statistics, related to Figure 4

	**GGG-bound P2RY8-G_{13}**
EMDB: Full map	EMD-47912
EMDB: 7TM map	EMD-47914
RCSB PDB: Model	9ECJ
**Data collection**	
Microscope	Thermo Scientific Krios G3i
Detector	Thermo Scientific Falcon 4i with Selectris X energy filter
Voltage (kV)	300
Magnification	130,000
Defocus range (µm)	-0.8 to -2.1
Pixel size, physical (Å)	0.94
Total exposure ($e^-/Å^2$)	50
Images, number of	12,053
EER fractions	80
Initial particles, number of	10,117,438
Final particles, number of	90,243
Symmetry imposed	C1
Map sharpening, *B* factor ($Å^2$)	
Full map	-79.5
7TM map	-83.0
Map resolution, masked (Å)	
Full map	2.9
7TM map	2.7
FSC threshold	0.143
**Refinement**	
Initial model used (AlphaFold code)	Q86VZ1
Model resolution (Å)	3.4
FSC threshold	0.5
Model composition	
Chains	4
Non-hydrogen atoms	6,323
Protein residues	790
Ligands	1
B factors ($Å^2$)	
Protein	35.24
Ligand	34.49
R.m.s. deviations	
Bond length (Å)	0.013
Bond angles (°)	2.006
Validation	
MolProbity score	1.67
Clash score	6.34
Rotamer outliers (%)	0.29
Ramachandran plot	
Favored (%)	95.38
Allowed (%)	4.62
Disallowed (%)	0.00

Supplemental References:

S1. Rasmussen, S.G.F., DeVree, B.T., Zou, Y., Kruse, A.C., Chung, K.Y., Kobilka, T.S., Thian, F.S., Chae, P.S., Pardon, E., Calinski, D., et al. (2011). Crystal structure of the β2 adrenergic receptor-Gs protein complex. Nature *477*, 549–555. https://doi.org/10.1038/nature10361.
